# HSPB2 facilitates neural regeneration through autophagy for sensorimotor recovery after traumatic brain injury

**DOI:** 10.1172/jci.insight.168919

**Published:** 2023-08-22

**Authors:** Yichen Huang, Shan Meng, Biwu Wu, Hong Shi, Yana Wang, Jiakun Xiang, Jiaying Li, Ziyu Shi, Gang Wu, Yanchen Lyu, Xu Jia, Jin Hu, Zhi-Xiang Xu, Yanqin Gao

**Affiliations:** 1 State Key Laboratory of Medical Neurobiology, MOE Frontiers Center for Brain Science; Institutes of Brain Science; and; 2 Department of Neurosurgery, Huashan Hospital, Fudan University, Shanghai, China.; 3 Department of Anesthesiology, Shanghai Pulmonary Hospital, School of Medicine, Tongji University, Shanghai, China.

**Keywords:** Neuroscience, Autophagy, Behavior, Neurological disorders

## Abstract

Autophagy is a promising target for promoting neural regeneration, which is essential for sensorimotor recovery following traumatic brain injury (TBI). Whether neuronal heat shock protein B2 (HSPB2), a small molecular heat shock protein, reduces injury and promotes recovery following TBI remains unclear. In this study, we demonstrated that HSPB2 was significantly increased in the neurons of a TBI mouse model, patients, and primary neuron cultures subjected to oxygen/glucose deprivation and reperfusion treatment. Upon creating a tamoxifen-induced neuron-specific HSPB2 overexpression transgenic mouse model, we found that elevated HSPB2 levels promoted long-term sensorimotor recovery and alleviated tissue loss after TBI. We also demonstrated that HSPB2 enhanced white matter structural and functional integrity, promoted central nervous system (CNS) plasticity, and accelerated long-term neural remodeling. Moreover, we found that autophagy occurred around injured brain tissues in patients, and the pro-regenerative effects of HSPB2 relied on its autophagy-promoting function. Mechanistically, HSPB2 may regulate autophagy possibly by forming the HSPB2/BCL2-associated athanogene 3/sequestosome-1 complex to facilitate the clearance of erroneously accumulated proteins in the axons. Treatment with the autophagy inhibitor chloroquine during the acute stage or delayed induction of HSPB2 remarkably impeded HSPB2’s long-term reparative function, indicating the importance of acute-stage autophagy in long-term neuro-regeneration. Our findings highlight the beneficial role of HSPB2 in neuro-regeneration and functional recovery following acute CNS injury, thereby emphasizing the therapeutic potential of autophagy regulation for enhancing neuro-regeneration.

## Graphical abstract

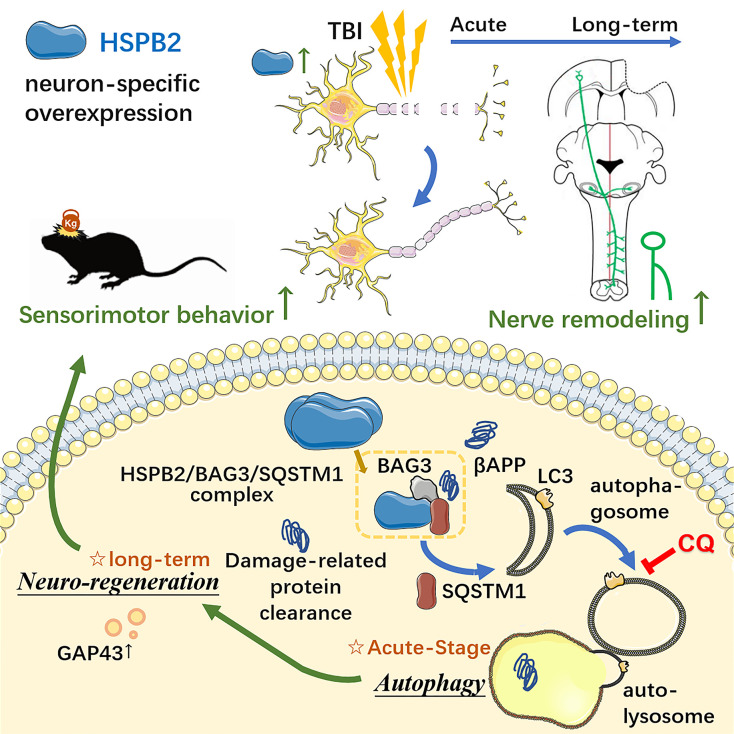

## Introduction

Traumatic brain injury (TBI) is a prevalent neurosurgical disease of the central nervous system (CNS) resulting in a significant number of disabilities and mortality worldwide, thereby imposing a substantial burden on both the family and society (1, 2). TBI pathogenesis is long-term and complex. Among its various aspects, severe white matter injury (WMI) associated with long-term sensorimotor and cognitive impairment has gained increasing attention (1, 3). In WMI, impaired neuronal axoplasmic transport, which is reflected by β-amyloid precursor protein (βAPP) deposition (4), causes secondary axonal degeneration. Moreover, TBI may cause aberrant protein aggregation, such as the accumulation of β-amyloid (Aβ) — the subsequent hydrolysate of βAPP — which is likely to trigger long-term neuronal death and cognitive impairment and contribute to the transition of TBI to Alzheimer disease (AD) (5). Besides, white matter repair is closely associated with the prognosis of TBI (6); thus, promoting white matter repair is crucial for the recovery of neural function. In spinal cord injury, the mTOR signaling pathway is closely associated with cell growth and axon regeneration (7). Furthermore, both neural plasticity and remodeling are involved in CNS repair (8). The contralateral or uninjured cerebral hemisphere can control injured limbs via axonal sprouting (9, 10). Therefore, alleviating WMI and promoting endogenous repair following TBI are promising therapeutic strategies for improving the prognosis of TBI.

Autophagy plays a vital role in clearing aberrant proteins and damaged organelles, as well as maintaining cellular homeostasis (11). Increasing evidence indicates that the level of neuronal autophagic proteins increases while autophagic flux decreases in the acute stage following TBI (12, 13), and both excessive and inhibited autophagy can exacerbate TBI damage (14, 15). Therefore, selective autophagy, which is triggered by autophagy substrates, has gained increasing attention. Selective autophagy is an autophagic process that employs the core machinery of macroautophagy but selectively targets specific cytosolic components for degradation (16). A novel target of selective autophagy is the co-chaperone BCL2-associated athanogene 3–mediated (BAG3-mediated) pathway, which leverages the specificity of molecular chaperones for misfolded proteins for mediating the degradation of misfolded or aberrantly aggregated proteins through macroautophagy (17, 18). Numerous studies have revealed the crosstalk between autophagy, apoptosis, and inflammation (19, 20). Several studies have demonstrated that autophagy regulation can protect neurons from death and reduce inflammatory reactions, thereby improving TBI prognosis (13, 21). However, little attention has been given to the association between autophagy and long-term neural regeneration. Axonal regeneration is a highly energy-demanding process (22). Autophagy supplies energy and materials for cellular renovation (23), thus supporting axonal regeneration. Thus, autophagy warrants further investigation as a potential mechanism of axonal regeneration. However, the role of autophagy in WMI and its repair following TBI warrants further investigation.

Heat shock protein B2 (HSPB2) is an important small heat shock protein (sHSP) (24). Cells exposed to various stressors, including high temperature, ischemia, hypoxia, and oxidative stress, can quickly synthesize a variety of HSPs (25). Numerous studies have demonstrated sHSPs to be involved in regulating the pathological processes of CNS diseases and that HSPs participate in the autophagy process (25). As molecular chaperones, sHSPs aid in proper protein folding and the degradation of misfolded protein (26). Our previous study demonstrated endothelial HSPB1 (HSP27) to inhibit actin polymerization in ischemic brain injury (27). HSPB8 participates in macroautophagy, acts as a chaperone with BAG3, and guides the degradation of misfolded proteins and mitochondria (17, 28). HSPB2 also reportedly binds to BAG3 (29). These findings suggest that HSPB2 improves TBI outcomes by promoting neuronal autophagy. In addition, the cell survival–promoting function of sHSPs is presumably related to their direct effect on kinase Akt (30), which is involved in various cell survival mechanisms and activates downstream mTOR for autophagy regulation (31).

However, whether neuronal HSPB2 reduces injury and promotes recovery following TBI remains unclear. In this study, we found that HSPB2 potentially participates in autophagy possibly through the HSPB2/BAG3/sequestosome-1 (SQSTM1) complex, guiding the clearance of erroneously accumulated axonal proteins, such as βAPP, to facilitate neural regeneration following TBI, thereby revealing the importance of acute-stage autophagy in long-term neuro-repair. This study provides valuable information concerning neuronal HSPB2’s neuroprotective and pro-recovery effects on TBI and aims to develop a potentially novel therapeutic target for acute CNS injuries.

## Results

### HSPB2 is upregulated in neurons following TBI in the mouse model and human patients.

First, we assessed the expression pattern of HSPB2 in the brain under physiological conditions. Immunofluorescence staining detected low levels of HSPB2 expression in the neurons of the cortex and hippocampus (Supplemental Figure 1, A and B; supplemental material available online with this article; https://doi.org/10.1172/jci.insight.168919DS1), which was consistent with a previous study in rats (32).

**Figure 1:**
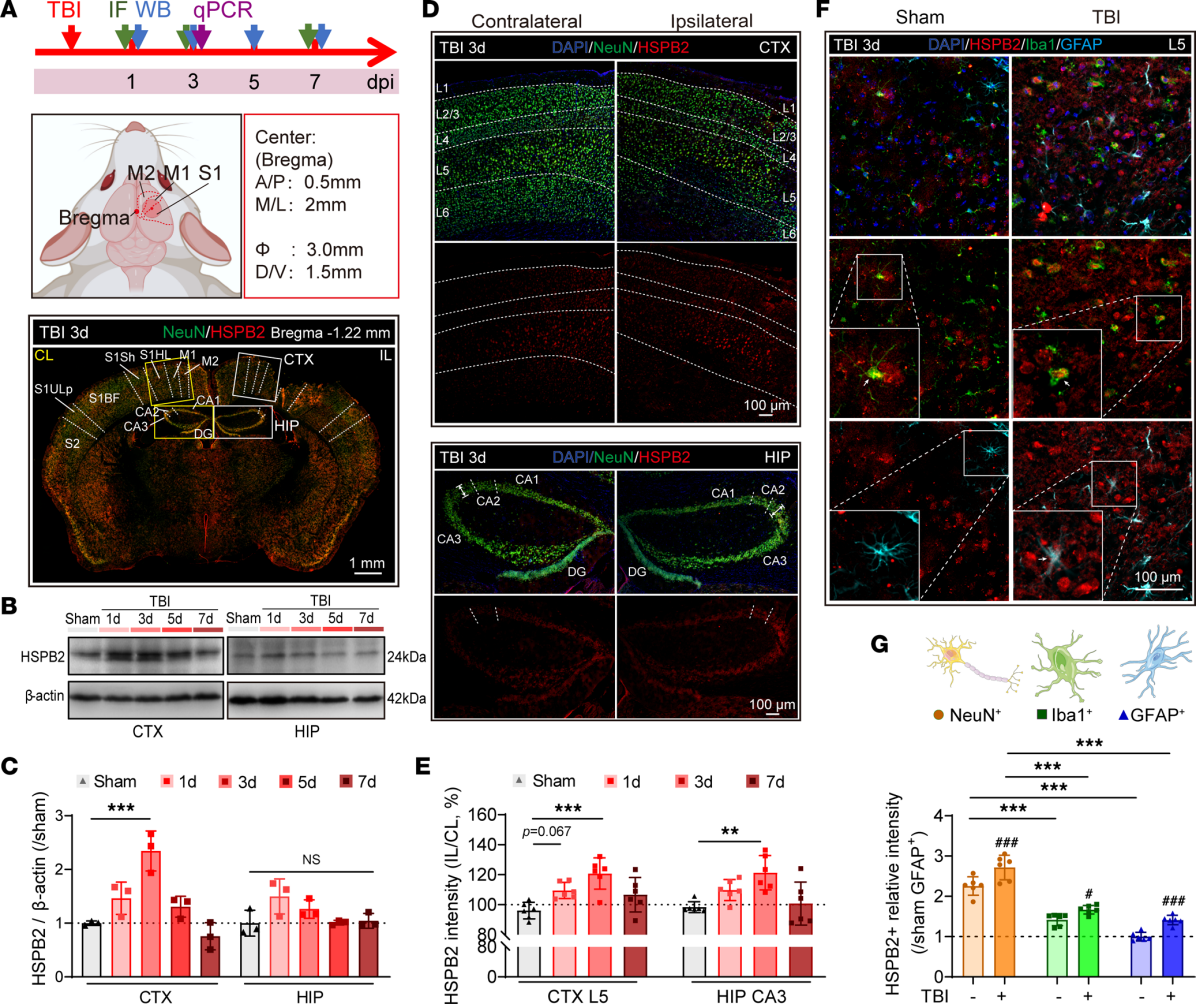
Upregulation of HSPB2 in neurons following TBI in mice. (**A**) Experimental design for HSPB2 expression tests in mice, CCI, and regions of CTX and HIP. Scale bar = 1 mm. (**B** and **C**) Representative Western blot and quantitative analysis of HSPB2 relative expression over time. *n* =3, analyzed by 1-way ANOVA and post hoc Bonferroni’s test. (**D**) Representative HSPB2 (red)/NeuN (green)/DAPI (blue) triple staining 3 days after CCI in CTX and HIP; scale bar = 100 μm; arrowed line indicates neuron layer width. (**E**) Quantitative analysis of HSPB2 expression over time in CTX neuron layer 5 (L5) and HIP CA3 region. *n* = 6, analyzed by 1-way ANOVA and post hoc Bonferroni’s test. (**F**) Representative HSPB2 (red)/Iba1 (green)/GFAP (cyan)/DAPI (blue) quadruple staining at 3 days after CCI in CTX L5. Box indicates magnified region, arrow indicates colabeled cells, scale bar = 100 μm, and insets = 2-fold original magnification. (**G**) Quantitative analysis of HSPB2 expression in different cell types. *n* = 6, analyzed by 2-way ANOVA and post hoc Bonferroni’s test. **: *P* < 0.01, ***: *P <* 0.001, as indicated. ^#^: *P* < 0.05, ^###^: *P <* 0.001. ^#^TBI versus sham. dpi, days postinjury; IL, ipsilateral; CL, contralateral; S1HL, primary somatosensory cortex, hind limb region; S1Sh, primary somatosensory cortex, shoulder region; S1BF, primary somatosensory cortex, barrel field; S1ULp, primary somatosensory cortex, upper lip region; DG, dentate gyrus; CTX, cortex; HIP, hippocampus; CA3, Cornu Ammonis 3 of hippocampus; Iba1, ionized calcium-binding adaptor molecule 1; GFAP, glial fibrillary acidic protein.

We subsequently examined the dynamic changes in HSPB2 following TBI. After controlled cortical impact (CCI) (Figure 1A), Western blotting revealed that the HSPB2 expression levels in the cortex surged approximately 2.5 times 3 days post-TBI but returned to normal levels within 7 days (Figure 1, B and C). However, the HSPB2 expression levels were not significantly altered in the full scale of the hippocampus following TBI (Figure 1, B and C), possibly owing to less damage to the hippocampus in this CCI model. HSPB2 expression was confirmed in the peri-injury area of the cortex using immunostaining, which revealed HSPB2 was upregulated on day 1 (*P* = 0.067) and significantly increased 3 days post-TBI in the neurons of the cortical layer 5 (L5), accompanied by a decrease in neuronal density (Figure 1, D and E, and Supplemental Figure 1, C and D). Meanwhile, in the CA3 region of the anterior hippocampus, even though there was no change in the width of the neuronal layer, a similar elevation in HSPB2 levels was observed 3 days post-TBI (Figure 1, D and E, and Supplemental Figure 1, E and F). Similarly, the elevated HSPB2 levels in L5 and CA3 did not persist beyond 7 days (Figure 1, D and E, and Supplemental Figure 1, C–F). The *Hspb2* mRNA levels were tested to verify that the elevation of HSPB2 was induced by expression rather than by degradation failure. *Hspb2* expression was significantly upregulated 3 days post-TBI, which verified that the increased HSPB2 levels were related to the upregulated expression (Supplemental Figure 1G). Changes in *Hspb1*, -*3*, and -*5* were also detected following TBI; however, none of them was significantly upregulated at 3 days post-TBI (Supplemental Figure 1G).

Next, we assessed the HSPB2 levels in the glial cells. Although the HSPB2 levels increased in the microglia and astrocytes 3 days post-TBI, the neurons revealed a much higher HSPB2 level than glial cells, indicating that neurons were the major contributors to the surge in the HSPB2 levels (Figure 1, F and G, and Supplemental Figure 1C). As anticipated, the transient expression pattern of HSPB2 during the acute stage of TBI was consistent with its properties as a stress protein (25).

**Figure 2:**
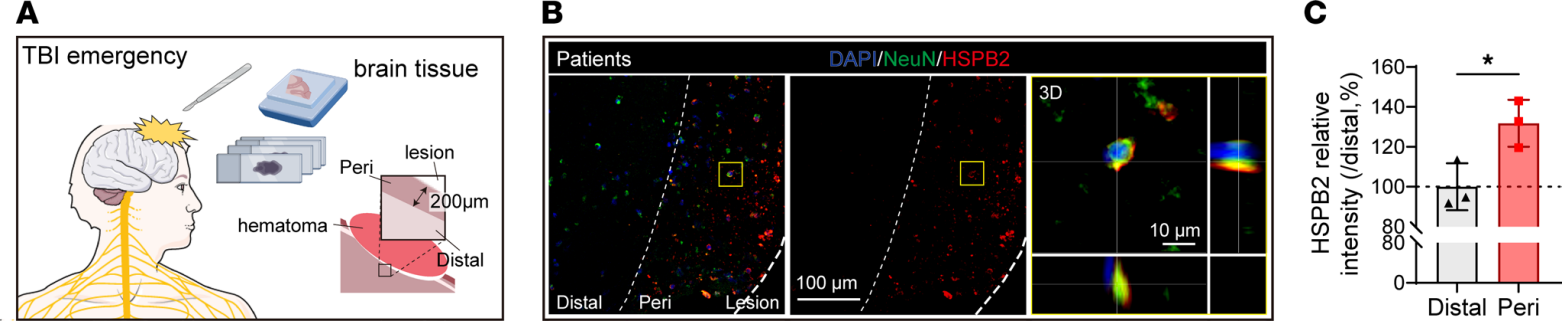
Upregulation of HSPB2 in neurons following TBI in patients. (**A**) Illustration of human patient specimen collection and the region of interest. (**B** and **C**) Representative images and quantitative analysis of neuronal HSPB2 in patients’ damaged tissues. Thick dotted line indicates lesion area (Lesion), thin dotted line indicates peripheral area (Peri, about 200 μm surrounding lesion area) and distal area (Distal, over 200 μm from lesion area), yellow box indicates enlarged 3D-scanned area, and scale bar = 100 μm & 10 μm. *n* = 3, analyzed by paired 2-tailed Student’s *t* test. *: *P* < 0.05.

Furthermore, we collected damaged brain tissues that were surgically removed from patients with brain contusion and laceration who underwent emergency neurosurgery. Importantly, we also found that HSPB2 was significantly upregulated in the neurons of the damaged tissue specimens of patients with acute brain contusion (Figure 2, A–C). Taken together, these results demonstrate that the expression levels of HSPB2 were dramatically increased in the neurons around the peri-injury site in both humans and mice, only in the acute stage, and not in the subacute stage following acute brain injury.

An in vitro primary neuron culture model verified alterations in neuronal HSPB2 expression in response to acute injury (Supplemental Figure 1H). Neurons were subjected to oxygen and glucose deprivation followed by reperfusion (OGD/r) to mimic the hypoxic environment following TBI (21). We verified an increase in the HSPB2 levels in the in vitro model (Supplemental Figure 1, I and J). The OGD/r model is also commonly used to simulate ischemic neuronal injury in stroke (33), suggesting that elevation of HSPB2 level is a universal phenomenon in acute neuronal injury.

### HSPB2 overexpression improves the sensorimotor outcomes and reduces tissue loss following TBI.

The observed surge in HSPB2 levels suggests HSPB2 plays a crucial role in TBI. Thus, we generated a conditional HSPB2 overexpression allele at the Rosa26 locus, which expresses hemagglutinin-tagged (HA-tagged) HSPB2 in a Cre-dependent manner. We initially overexpressed HSPB2 in the neurons by crossing mice with *Map2-CreERT2* mice, selectively expressing Cre in neurons, to generate *R26-e(CAG-LSL-Hspb2) Map2-CreERT2*, N-HSPB2, termed TG (Supplemental Figure 2, A and B). HSPB2 overexpression was confirmed by immunofluorescence staining and Western blotting using antibodies specifically against HA or HSPB2 (Supplemental Figure 2, E–H), with HSPB2 levels in TG mice elevated to over 10 times those in wild-type (WT) mice (Supplemental Figure 2H). Additionally, quantitative polymerase chain reaction verified the overexpression of *Hspb2* at the mRNA level (Supplemental Figure 2C). The expression of *Hspb1*, -*3*, and -*5* was unaffected by HSPB2 overexpression (Supplemental Figure 2D). A previous study suggested that HSPB2 activity is negatively regulated by HSPB3 interactions (29). In our study, *Hspb3* was not upregulated in a compensatory manner; thus HSPB3 did not regulate the activity of overexpressed HSPB2.

**Figure 3:**
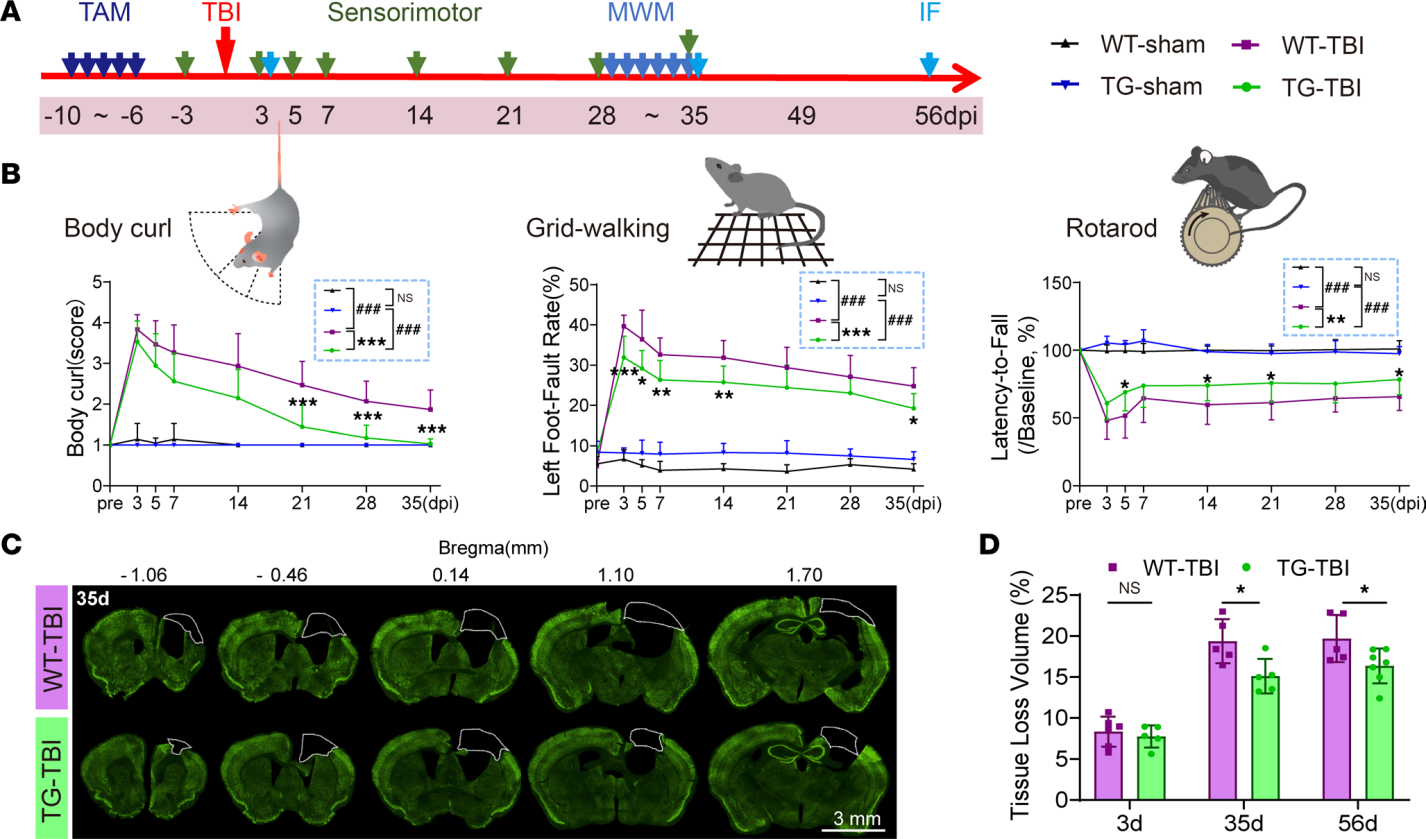
HSPB2 improves sensorimotor outcomes and reduces tissue loss following TBI. (**A**) Experimental design for neurofunction and tissue loss assessment. (**B**) Quantitative analysis of behavioral tests. *n* = 7 WT-sham, 7 WT-TBI, 15 TG-sham, and 17 TG-TBI, analyzed by 2-way ANOVA and post hoc Bonferroni’s test. (**C**) Illustration of tissue loss. Dotted boxes: loss area; green: NeuN; scale bar = 3 mm. (**D**) Quantitative analysis of tissue loss volume. *n* = 5–7, analyzed by 2-way ANOVA and post hoc Bonferroni’s test. *TG versus WT, ^#^TBI versus sham. *: *P* < 0.05, **: *P* < 0.01, ***/^###^: *P <* 0.001.

The long-term effects of HSPB2 on neurofunction were assessed using a series of behavioral tests 3 to 35 days following TBI (Figure 3A). No significant differences in the body weight were observed between the 2 TBI groups (Supplemental Figure 3A). The unilateral CCI model induced asymmetrical and sensorimotor functional deficits (34). The body curl test demonstrated that HSPB2 mitigated the degree of body asymmetry attributed to TBI (Figure 3B). However, no significant improvement in asymmetry was observed in the TG-TBI group in the cylinder test (Supplemental Figure 3B). Moreover, the TG-TBI mice exhibited enhanced motor abilities in the rotarod test (Figure 3B). The grid-walking test suggested that the fine motor regulation ability of the injured limb in the TG mice was significantly improved (Figure 3B). Taken together, these behavioral results demonstrated that neuron-specific HSPB2 overexpression improved the long-term sensorimotor outcomes in mice following TBI.

No significant differences in the swim velocity were observed between the groups in the Morris water maze (MWM) test (Supplemental Figure 3, C and D). The MWM test results also demonstrated that the spatial memory of the WT-TBI mice was significantly lower than that of the WT-sham group. In contrast, the difference between the TG-TBI and TG-sham groups was less pronounced, indicating that HSPB2 overexpression mitigated spatial learning and memory impairment following TBI (Supplemental Figure 3D).

Numerous preclinical studies have failed to demonstrate clinical translation because they used only male animals. Although men have a higher incidence of TBI, women experience equivalent consequences (2). Sex dimorphisms have been reported in the brain structure, metabolism, function, and response to TBI (35–37). Therefore, it is important to test candidate neurotherapeutics in TBI models of both sexes. We examined the behavior of female mice and found that HSPB2 overexpression significantly improved long-term sensorimotor behavior in female mice (Supplemental Figure 3E).

We also evaluated whether HSPB2 protected against brain tissue loss. In the acute stage (3 days after TBI), no significant differences were found between the genotypes (Supplemental Figure 4, A–C, and Figure 3, C and D). However, HSPB2 overexpression significantly reduced tissue loss and brain atrophy at 35 and 56 days post TBI (Figure 3, C and D, and Supplemental Figure 4, B and C). These results suggested that HSPB2 may not directly reduce primary cell loss, but rather, it may primarily reduce secondary neuronal death or promote tissue repair.

### HSPB2 enhances the structural and functional integrity of white matter following TBI.

**Figure 4:**
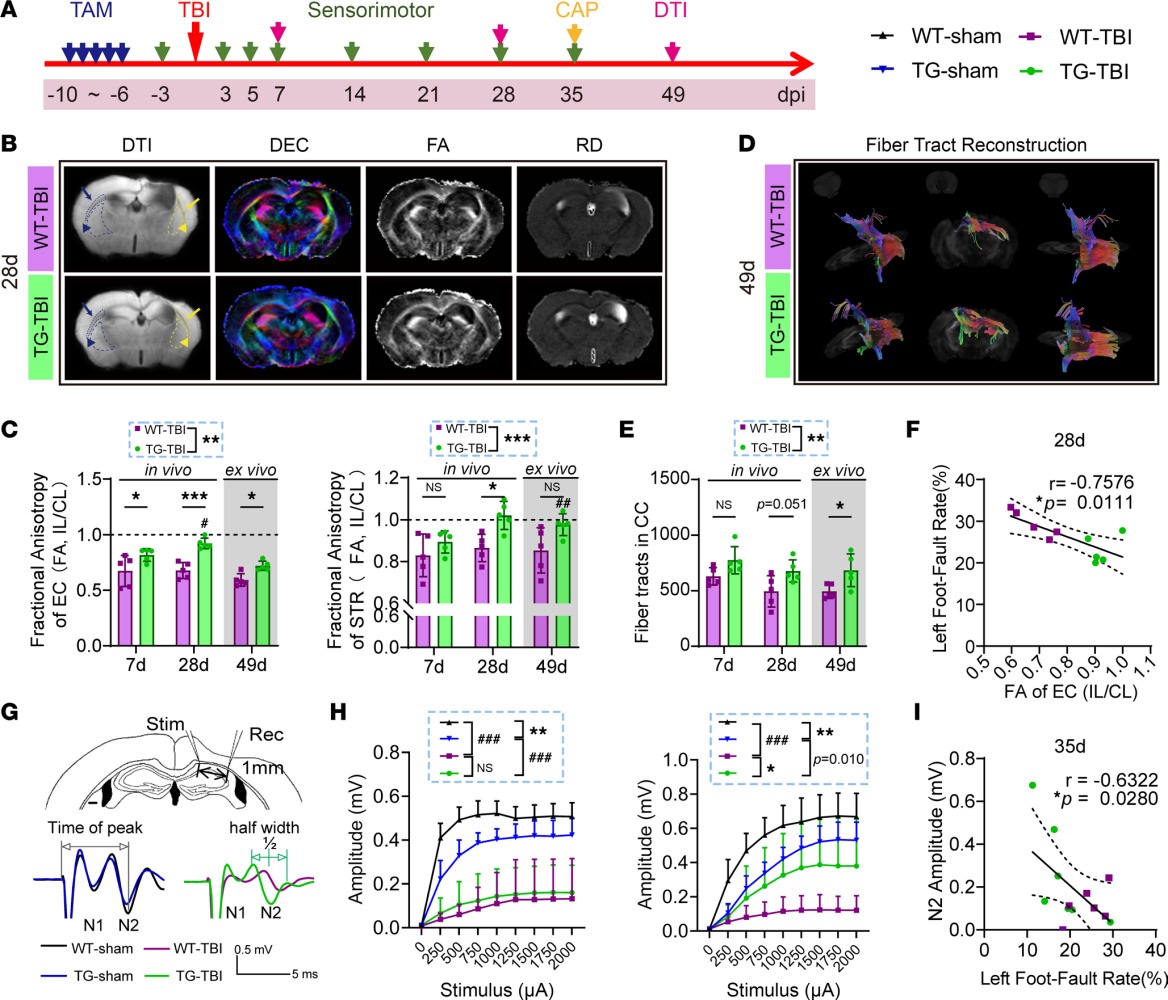
HSPB2 enhances white matter integrity following TBI. (**A**) Experimental design for white matter integrity assessment. (**B**) DTI-MRI and visualized parameters, including direction encoding color (DEC), fractional anisotropy (FA), and radial diffusivity (RD). Triangle-arrowed dotted boxes indicate region of interest (ROI) in the striatum (STR). Arrowed dotted boxes indicate ROI in the external capsule (EC). Yellow represents ipsilateral; blue represents contralateral. (**C**) Quantitative analysis of relative FA in the EC and STR at 7 and 28 days (in vivo) and 49 days (ex vivo) post-TBI. *n* = 5, *TG-TBI versus WT-TBI, ^#^indicated versus 7 days (7d). (**D**) Illustration of 3D reconstruction of fiber tracts. Color represents direction, from left to right: front side view, front view, and top view of fiber tracts across middle of CC. (**E**) Quantitative analysis of the number of fiber tracts across the middle of CC. *n* = 5, *TG-TBI versus WT-TBI, ^#^versus 7d. (**F**) Correlation analysis between the left forelimb fault rate in grid-walking test and FA of the EC at 28 days postinjury. *n* = 5, analyzed by Spearman’s correlation test. (**G**) Illustration of compound action potential (CAP). Stim, stimulating electrode; Rec, recoding electrode. (**H**) Stimulus-amplitude curve of N1 and N2, *n* = 6–7. *TG-TBI versus WT-TBI, ^#^TBI versus sham, or as indicated. (**I**) Correlation analysis between left forelimb fault rate in grid-walking test and N2 amplitude at 1,000 μA stimulus at 35 days postinjury, *n* = 6&7, analyzed by Spearman’s correlation test; **C**, **E**, and **H** by 2-way ANOVA and post hoc Bonferroni’s test. */^#^: *P* < 0.05, **/^##^: *P* < 0.01, ***/^###^: *P <* 0.001.

We investigated the structural integrity of the white matter using diffusion tensor imaging–MRI (DTI-MRI) in vivo on days 7 and 28 and ex vivo on day 49 (Figure 4A and Supplemental Figure 5, A–E). Fractional anisotropy (FA), which reflects the fine structure of white matter (38), showed a significant improvement in the external capsule (EC) of the TG-TBI group compared with that of WT-TBI group at each time point, with FA in the striatum (STR) also improving on day 28 (Figure 4, B and C). Radial diffusivity (RD), an indicator of demyelination (38), decreased in the EC of the TG-TBI group on day 49 (Supplemental Figure 5E). Notably, FA in the EC recovered on day 28 compared with day 7, and FA in the striatum significantly increased on day 49 in the TG group (Figure 4, B and C). Moreover, the RD of the striatum significantly decreased on days 28 and 49 compared with day 7 in the TG group (Supplemental Figure 5E). These results suggested a pro-repair effect of HSPB2 on WMI. We further reconstructed the white matter fiber tract projections (Figure 4D and Supplemental Figure 5, C and D). We examined the projections of the fiber tracts across the middle of the corpus callosum (CC). A significant increase in the number of fiber tracts in the TG-TBI group was observed at 49 days post-TBI, with a trend observed on day 28 (Figure 4, D and E, and Supplemental Video 1), further supporting the notion that HSPB2 improves white matter integrity by promoting the repair of white matter fibers. Additionally, the correlation matrix revealed a strong relationship between DTI and behavioral parameters at each time point, particularly between the FA of the EC and grid-walking on day 28 (Figure 4F, Supplemental Figure 6, and Supplemental Table 1). Collectively, these results suggest a close association between white matter structure repair and behavioral improvement.

To assess the functional integrity of the white matter, we performed electrophysiological recordings by detecting the compound action potentials (CAPs) in the CC 35 days following TBI (Figure 4, A and G) (39). CAP recordings reflect the combined conduction of both unmyelinated and myelinated fibers in the CC. The amplitudes of the faster action potential N1 and slower action potential N2 represent the electrical conduction of the myelinated and unmyelinated fibers, respectively. We found that HSPB2 overexpression significantly prevented the TBI-induced decrease in N2 amplitude but not in N1 amplitude (Figure 4H). Meanwhile, the amplitude of N2 negatively correlated with the foot fault rate, suggesting that the enhancement of white matter functional integrity supports behavioral improvement (Figure 4I). The peak time of the N1/N2 potential represents the conduction velocity, whereas the potential’s half-width represents the synchronization of conduction. HSPB2 overexpression also prevented delay in the N2 peak time and extension of the N2 half-width (Supplemental Figure 5F). Correlation analysis further suggested that the N2 peak time and half-width were associated with the foot fault rate on day 35 (Supplemental Figure 5G), indicating that the speed and synchronization of the axon action potential were closely related to behavioral performance. Taken together, the callosal CAP results suggest that HSPB2 confers partial protection against the loss of white matter integrity by improving the electrical conduction of unmyelinated axonal fibers.

Additionally, we assessed the synaptic density in the peri-injury cortex. The density of synaptophysin, a presynaptic protein, significantly decreased following TBI. However, HSPB2 overexpression partially reversed this reduction in TG mice (Supplemental Figure 5, H and I). In conclusion, these data demonstrate that HSPB2 enhances the long-term structural and functional integrity of white matter.

### HSPB2 enhances CNS plasticity and facilitates neural remodeling through axon regeneration.

**Figure 5:**
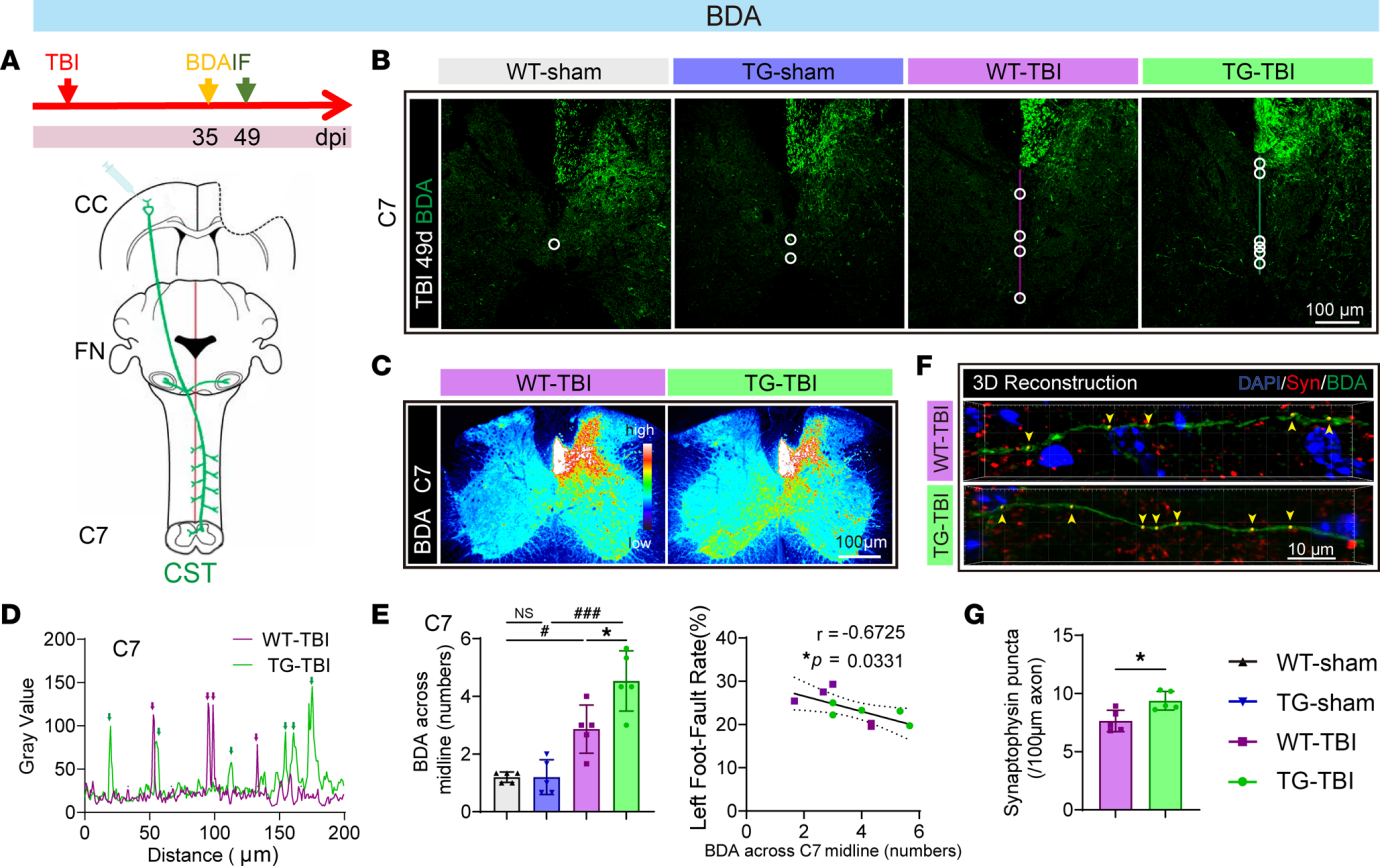
HSPB2 facilitates axon sprouting in the long term after TBI. (**A**) Experimental design and sketch of anterograde tracing of CST. (**B**) Illustration of BDA-labeled axons crossing the midline of C7 at 49 days postinjury; circle indicates the intersection of axon and midline, and line represents the midline for plot profile analysis. (**C**) Heatmap of BDA fluorescence intensity. (**D**) Plot profile of the C7 midline in **B**; arrow indicates the BDA intersection. (**E**) Quantitative analysis of BDA^+^ axons crossing the midline in C7 and correlation between BDA^+^ axons across the midline in C7 and total foot fault rate at day 49. *n* = 5, analyzed by 1-way ANOVA and post hoc Bonferroni’s test and Spearman’s correlation test. (**F**) Representative 3D reconstruction of BDA^+^ axons with synaptophysin^+^ vesicles in the contralateral C7. Blue, DAPI; red, synaptophysin. (**G**) Quantitative analysis of synaptophysin^+^ vesicles in BDA^+^ axons (*n* = 5), analyzed by unpaired 2-tailed Student’s *t* test. *TG versus WT, ^#^TBI versus sham. *: *P* < 0.05, ^###^: *P <* 0.001. BDA, biotin dextran amine; C7, cervical spinal cord segment 7; FN, facial nucleus layer.

Neural regeneration and remodeling reportedly contribute to neurological recovery. The recruitment of spared circuits in the contralateral cortex shows promise for neuro-recovery, as reflected by compensatory axon sprouting and cortical remapping. We used anterograde tracing to explore compensatory axon sprouting in the uninjured (contralateral) corticospinal tract (CST) (Figure 5A and Supplemental Figure 7A). BDA is anterogradely transported along the pyramidal tracts and crosses the contralateral (ipsilesional) spinal cord before C7 (9). The number of BDA^+^ fibers crossing the midline was used to quantify CST crossing and compensatory axon sprouting. Our results demonstrated that the natural cross-connection between the CC in the cerebrum and medulla oblongata FN was reduced 49 days post-TBI, whereas HSPB2 recovered the decrease in the connection at the FN (Supplemental Figure 7, B and C). On day 49, compensatory axon sprouting at C7 was robust (Figure 5, B and E), which was consistent with the results of previous studies (9). As anticipated, HSPB2 significantly facilitated compensatory axon sprouting on day 49 (Figure 5, B–E). Moreover, the number of BDA^+^ fibers crossing the C7 midline negatively correlated with the foot fault rate, suggesting that HSPB2 may improve sensorimotor behaviors through neural remodeling (Figure 5E). We also found that the number of synaptophysin^+^ (a synaptic marker) vesicles transported along the BDA-labeled axons increased on the compensatory side of C7 in TG-TBI mice (Figure 5, F and G, and Supplemental Figure 7D). Synaptic vesicle proteins, such as synaptophysin, play a crucial role in axon growth (40), thereby supporting the notion that HSPB2 facilitates axonal regeneration.

**Figure 6:**
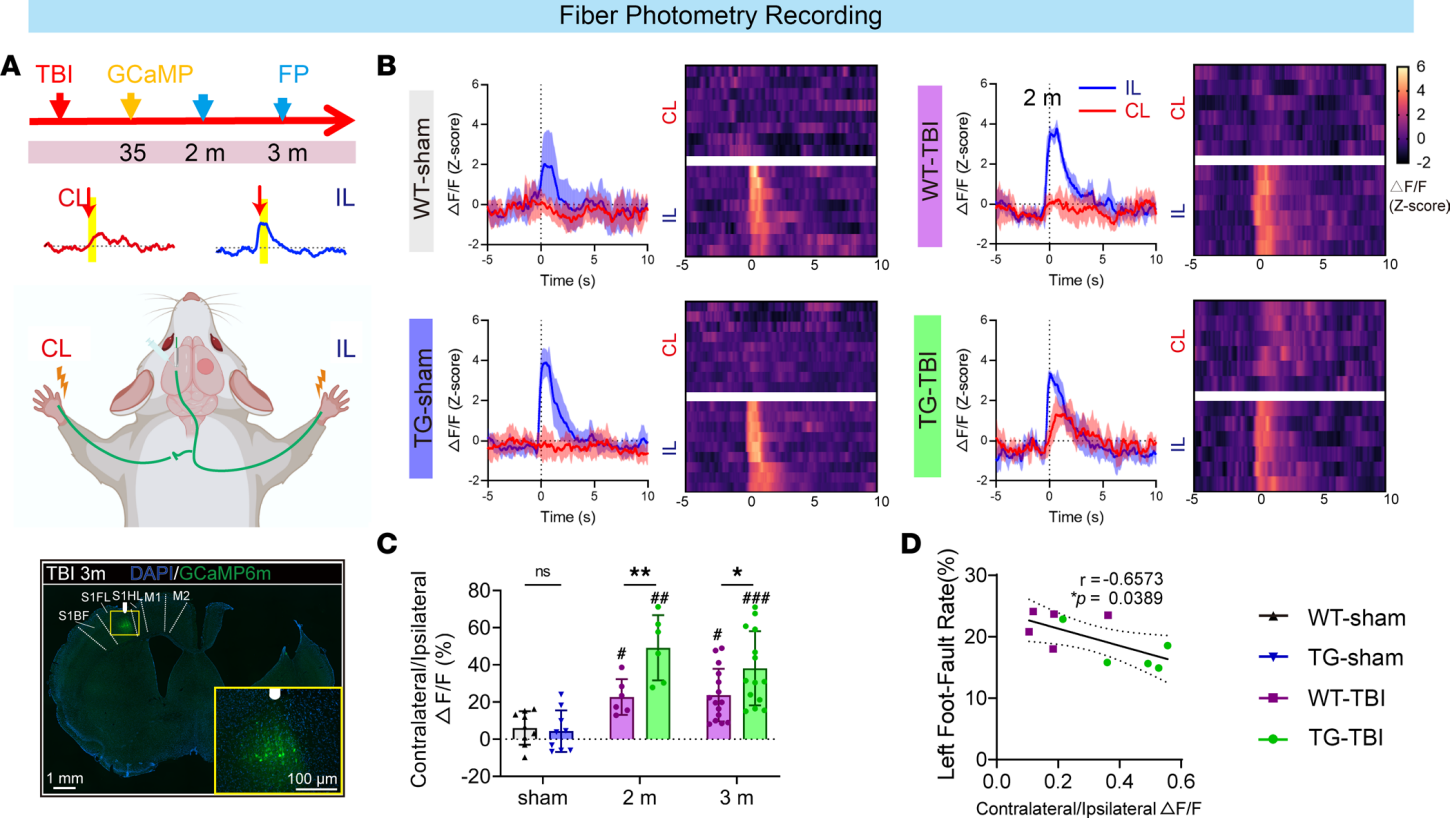
HSPB2 enhances cortical remapping in the long term after TBI. (**A**) Experimental design, sketch of contralateral sensory neuron calcium activity recording, and illustration of the AAV-GCaMP injection area; yellow box indicates magnified area; white column represents optical fiber location; and blue, DAPI. (**B**) Line plots and heatmaps of S1FL cortex fluorescence responses for each forepaw at 2 months; blue: ipsilateral, red: contralateral. (**C**) Quantitative analysis of contralateral/ipsilateral forepaw ΔF/F, *n* = 3 × 3 trials (sham), 2 × 3 trials (2 months), 5 × 3 trials (3 months), analyzed by 2-way ANOVA and post hoc Bonferroni’s test. (**D**) Spearman’s correlation between contralateral/ipsilateral forepaw ΔF/F and total foot fault rate at 3 months, analyzed by Spearman’s correlation test. *TG versus WT, ^#^TBI versus sham. */^#^: *P* < 0.05, **/^##^: *P* < 0.01, ^###^: *P <* 0.001. IL, ipsilateral; CL: contralateral.

To investigate cortical remapping and assess the function of the compensatory CST in vivo, we recorded the neuronal activity in the contralateral S1FL in response to tactile stimulation applied to each forepaw, following stereotactic injection of AAV-hSyn-GCaMP6m at the S1FL and optical fiber implantation (Figure 6A). At 2 and 3 months post-TBI, we observed that contralateral cortical neuronal activity significantly increased in response to the ipsilateral (injured) forepaw stimulus compared with that in the sham group, indicating cortical remapping (Figure 6, B and C, and Supplemental Figure 7, E and F). The response of the contralateral (uninjured) forepaw remained unchanged (Figure 6B and Supplemental Figure 7G). Additionally, HSPB2 enhanced the response amplitude 2 months following TBI (Figure 6B and Supplemental Figure 7F). At 2 and 3 months following TBI, the ratio of the response amplitudes for the contralateral to ipsilateral forepaw significantly increased, indicating that HSPB2 overexpression facilitated cortical remapping (Figure 6, B and C, and Supplemental Figure 7E). At 3 months, the amplitude of the contralateral S1FL response to the injured forepaw correlated with fine sensorimotor behavior, suggesting that cortical remapping aids the sensorimotor function recovery (Figure 6D).

In summary, these results suggest that neuronal HSPB2 promotes CNS plasticity and facilitates the neural remodeling processes, including axon sprouting and cortical remapping, which contribute to the sensorimotor function recovery.

### HSPB2 elevates autophagy of bilateral cortical neurons at the acute stage.

**Figure 7:**
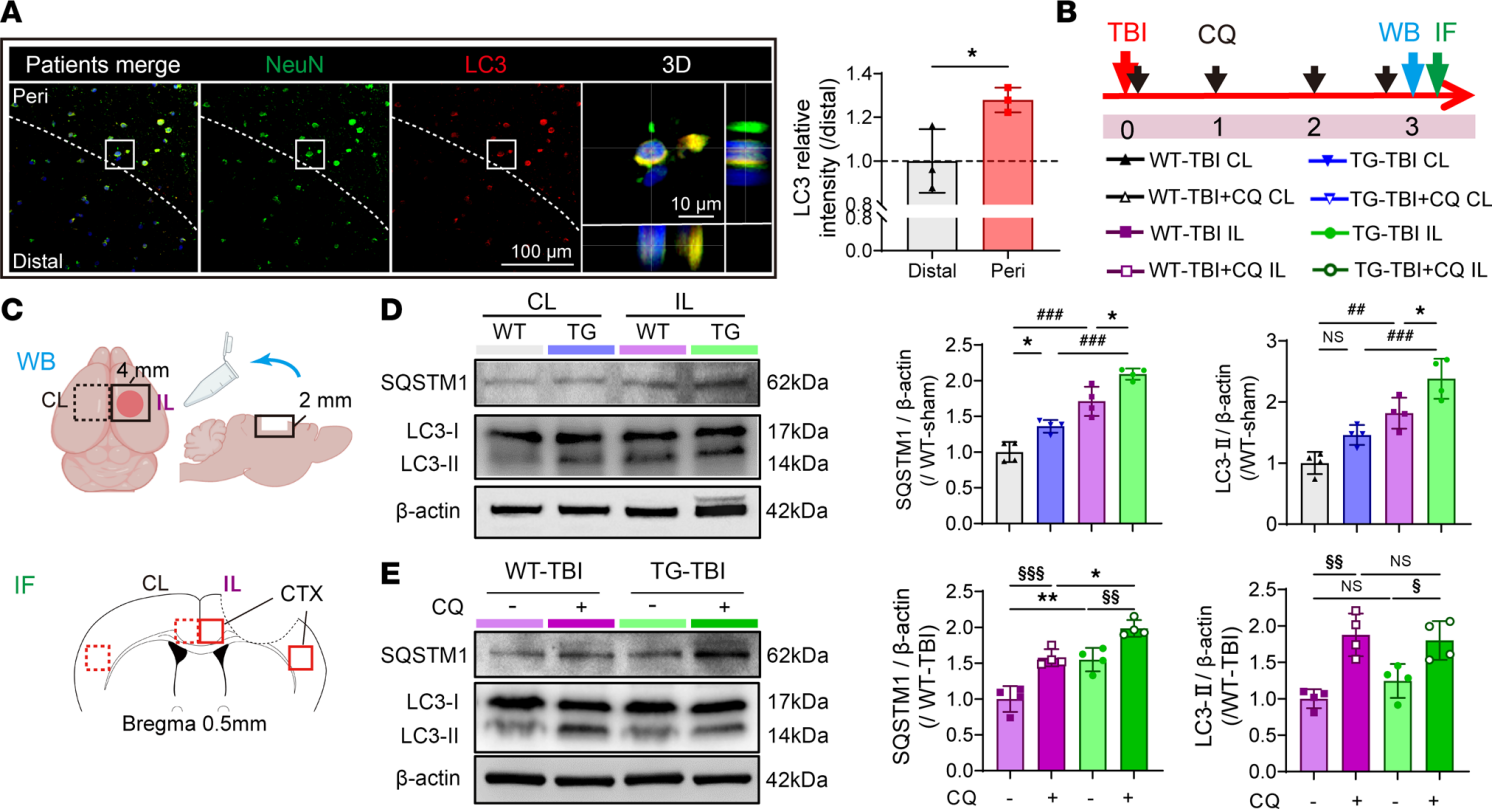
HSPB2 significantly promotes neuronal autophagy at the acute stage following TBI. (**A**) Illustration and quantitative analysis of LC3 in patients, dotted line indicates peripheral area (Peri) and distal area (Distal). Blue: DAPI. Scale bar = 100 μm. *n* = 3, analyzed using paired 2-tailed Student’s *t* test. (**B**) Experimental design for mouse model. (**C**) Tissue location used for Western blot, and the ROI of IF. (**D**) Illustration and quantitative analysis of SQSTM1 and LC3-I&II in CTX at 3 days postinjury. The 4 groups are WT-TBI CL, TG-TBI CL, WT-TBI IL, and TG-TBI IL (left to right). *n* = 4, analyzed using 1-way ANOVA and post hoc Bonferroni’s test. (**E**) Illustration and quantitative analysis of SQSTM1 and LC3-I&II in CTX at day 3 with/without administration of CQ. The 4 groups are WT-TBI IL, WT-TBI+CQ IL, TG-TBI IL, and TG-TBI+CQ IL (left to right). *n* = 4, analyzed using 1-way ANOVA and post hoc Bonferroni’s test. *TG versus WT, ^#^IL versus CL, ^§^CQ versus non-CQ, or as indicated. *: *P* < 0.05, ^##^/^§§^: *P* < 0.01, ^###^/^§§§^: *P <* 0.001. IL, ipsilateral; CL: contralateral.

Previous studies have demonstrated that neuronal autophagy peaks at 3 days post-TBI (13), which is consistent with our data on HSPB2 expression pattern following TBI (Figure 1C). In the injured brain tissue of patients, the microtubule-associated protein 1A/1B-light chain 3 (LC3) levels increased on the peri-injury side (Figure 7A), indicating acute activation of autophagy in the neurons. We investigated whether HSPB2 overexpression affected neuronal autophagy 3 days post-TBI in the peri-injury cortex (Figure 7, B and C). SQSTM1, a ubiquitin-binding autophagy receptor that transports autophagy substrates, was significantly increased 3 days post-TBI in both WT and TG mice in the ipsilateral cortex (Figure 7D), suggesting a stress response following injury and the accumulation of autophagic substrates (41). LC3-II, another crucial autophagosome marker, was also significantly increased as anticipated, thereby verifying autophagy activation post-TBI (Figure 7D). The LC3 II/I ratio, which indicates autophagic LC3 transformation, increased similarly (Supplemental Figure 8A). Notably, SQSTM1 and LC3-II further increased in the ipsilateral cortex of the TG-TBI group, suggesting that the TG-TBI mice had increased levels of autophagic substrates and autophagy (Figure 7, D and E). The immunofluorescence results for LC3 and SQSTM1 were consistent with those of Western blotting, with a significant increase in the proportion of cortical LC3- and SQSTM1-positive neurons in the TG-TBI group in the ipsilateral cortex (Supplemental Figure 8, C and D). Overall, HSPB2 overexpression substantially enhanced postinjury autophagy. The contralateral cortex of TG mice also exhibited higher SQSTM1 levels than the contralateral cortex of WT mice in Western blotting and immunofluorescence (Figure 7D and Supplemental Figure 8, C and D). The LC3-II levels in the contralateral cortex of TG mice showed increased immunofluorescence (*P* = 0.071, Supplemental Figure 8, C and D). These results demonstrated that HSPB2 also increases autophagy in contralateral cortical neurons at the acute stage.

Elevated SQSTM1and LC3-II protein levels may also result from blockage of autophagic flux (42). TBI can cause lysosomal membrane damage, leading to the inhibition of autophagy (43). To address this, chloroquine (CQ), an autophagic inhibitor that neutralizes lysosomal pH to hinder autophagosome degradation, was administered to block the autophagic flux and determine whether HSPB2 impaired it. We found that CQ further accumulated SQSTM1 and LC3-II in the TG group, as CQ impeded their degradation by autolysosomes, indicating that HSPB2 did not impair autophagic flux (Figure 7E and Supplemental Figure 8B).

**Figure 8:**
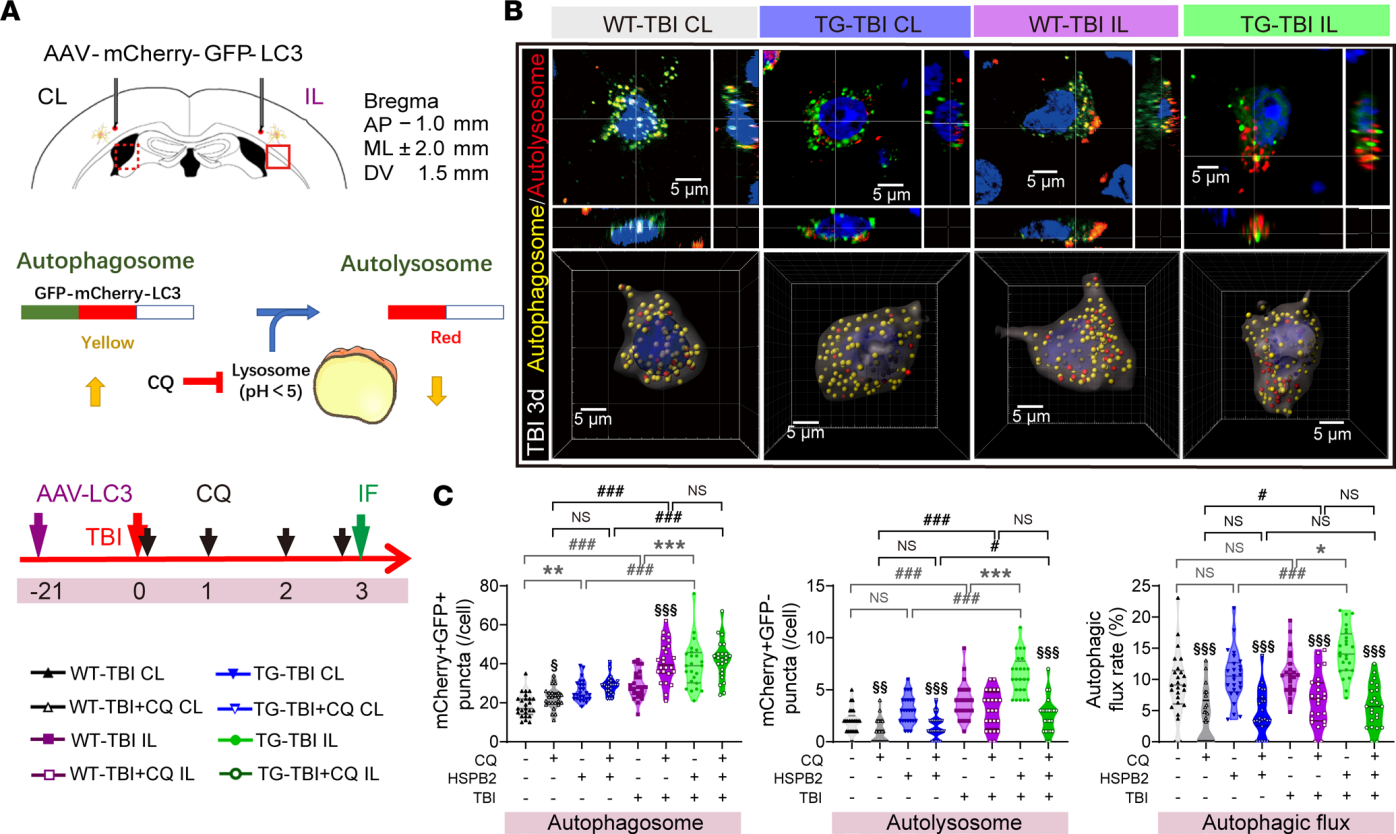
HSPB2 significantly promotes autophagic flux following TBI. (**A**) Illustration of intracerebral injection of AAV2/9-CMV-mCherry-EGFP-LC3, the function of AAV and CQ, and the experimental design; autophagosome membrane LC3 is labeled with both mCherry and GFP. Upon fusion with a lysosome to form autolysosome, the acidic environment quenches GFP, so mCherry alone represents autolysosome. Meanwhile CQ inhibits the acidic environment of the lysosome, blocking autolysosome formation, leading to a decrease in mCherry^+^ autolysosome and an increase of mCherry^+^GFP^+^ autophagosome. (**B**) 3D confocal images (top) and 3D reconstruction (bottom) of AAV2/9-CMV-mCherry-EGFP-LC3–labeled neuron in CTX. Blue: DAPI; red (mCherry^+^GFP^–^): autolysosome vesicles; yellow (mCherry^+^GFP^+^): autophagosome vesicles. Scale bar: 5 μm. (**C**) Quantitative analysis of autophagosome, autolysosome, and autophagic flux rate (autolysosome/autophagosome) displayed as violin plots with means and quartiles. The 8 groups are WT-TBI CL, WT-TBI+CQ CL, TG-TBI CL, TG-TBI+CQ CL, WT-TBI IL, WT-TBI+CQ IL, TG-TBI IL, and TG-TBI+CQ IL (left to right). *n* = 5 × 4–5 cells/hemisphere, analyzed using 1-way ANOVA and post hoc Bonferroni’s test. *TG versus WT, ^#^IL versus CL, ^§^CQ versus non-CQ, or as indicated. */^#^/^§^: *P* < 0.05, **/^§§^: *P* < 0.01, ***/^###^/^§§§^: *P <* 0.001. IL, ipsilateral; CL: contralateral; HSPB2-/+, WT-TBI/TG-TBI group; TBI-/+, contralateral/ipsilateral.

To better validate HSPB2’s role in enhancing autophagy without harming autophagic flux, we injected AAV-CMV-mCherry-EGFP-LC3 virus to label and differentiate the autophagosomes and autolysosomes based on the instability of enhanced green fluorescent protein (EGFP) in the acidic lysosomal environment and quantified the autophagic flux (42, 43) (Figure 8A). Three-dimensional reconstruction revealed an increase in the number of yellow-fluorescent-labeled autophagosomes and red-fluorescent-labeled autolysosomes following TBI (Figure 8B). CQ administration reduced the number of autolysosomes and accumulated autophagosomes attributed to lysosomal dysfunction (Figure 8B and Supplemental Figure 8E). The TG-TBI group had a significantly higher number of autophagosomes than the WT-TBI group in both the ipsilateral and contralateral regions, whereas the number of autolysosomes increased in the TG-TBI group in the ipsilateral region (Figure 8C and Supplemental Figure 8E). No significant differences were observed between the 2 groups following CQ administration (Figure 8C and Supplemental Figure 8E). The autophagic flux rate (the ratio of autolysosomes to autophagosomes) represents the autophagic flux, which significantly increased in the TG-TBI group in the ipsilateral region but decreased significantly after CQ administration (Figure 8C and Supplemental Figure 8E). These results suggested that HSPB2 promotes autophagic flux.

### The effects of HSPB2 on promoting neuronal autophagy are independent of the Akt/mTOR signaling pathway.

**Figure 9:**
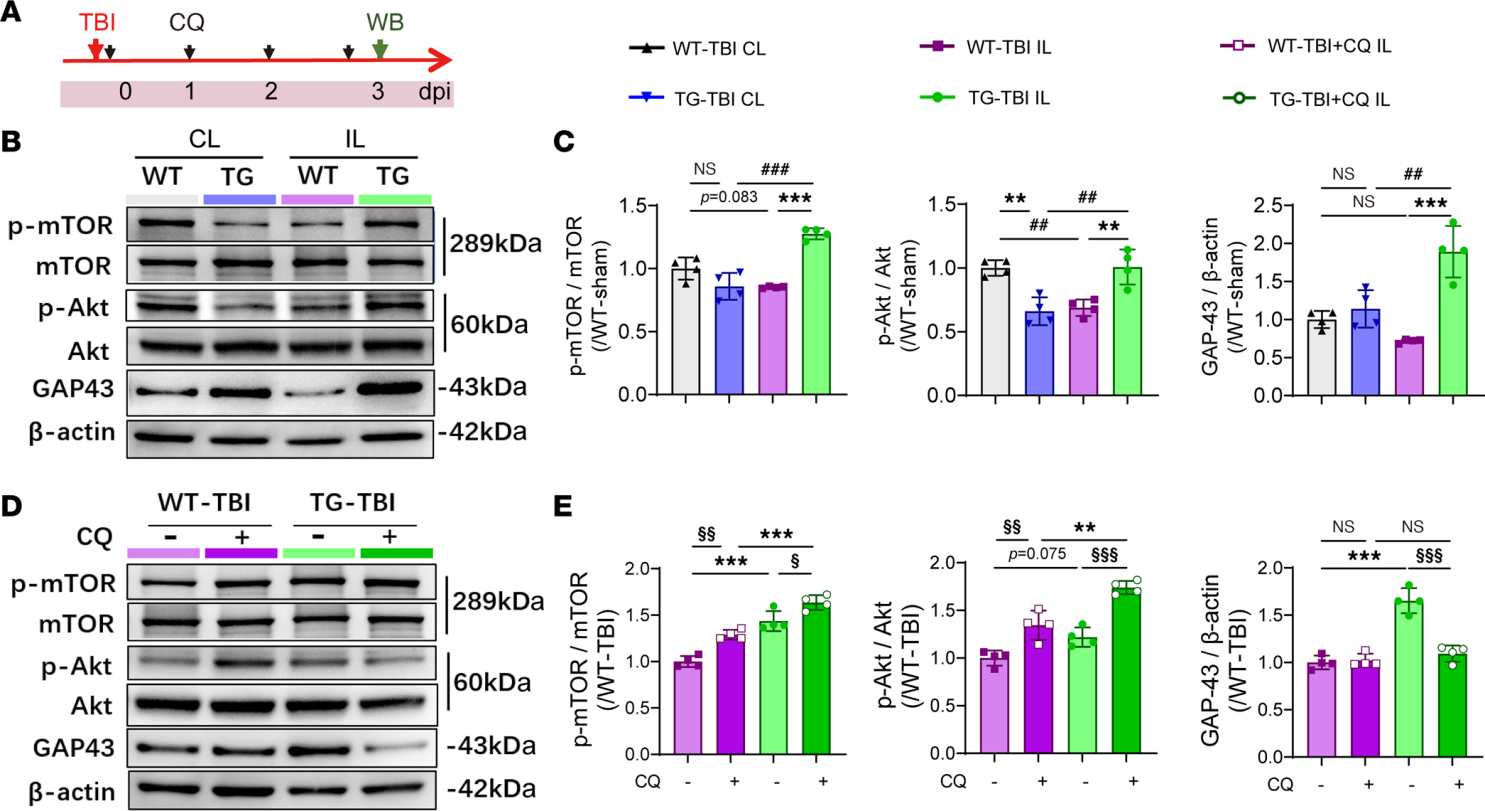
Changes in Akt, mTOR, and GAP43 following TBI with HSPB2 overexpression and CQ treatment. (**A**) Experimental design related to signaling pathway. (**B** and **C**) Illustration and quantitative analysis of p-mTOR, p-Akt, and GAP43 in CTX at 3 days postinjury. *n* = 4, analyzed using 1-way ANOVA and post hoc Bonferroni’s test. (**D** and **E**) Illustration and quantitative analysis of p-mTOR, p-Akt and GAP43 in CTX at 3 days postinjury with CQ administration, *n* = 4, analyzed using 1-way ANOVA and post hoc Bonferroni’s test. *TG versus WT, ^#^IL versus CL, ^§^CQ versus non-CQ, or as indicated. ^§^: *P* < 0.05, **/^##^/^§§^: *P* < 0.01, ***/^###^/^§§§^: *P* < 0.001. GAP43, growth associated protein-43; p-, phosphorylated.

We attempted to determine the signaling pathways involved in the regulation of autophagy by HSPB2. HSPB1 interacts directly with Akt (30), which activates downstream mTOR for autophagy regulation (31). Moreover, the mTOR signaling pathway has been reported to be closely associated with axon regeneration (7). Therefore, we initially assessed the modulation of the Akt/mTOR signaling pathway by HSPB2 (Figure 9A). However, HSPB2 overexpression significantly enhanced Akt/mTOR signaling activation, whereas Akt and mTOR activation tended to decrease following TBI (Figure 9, B and C). This result contradicts the previous finding that HSPB2 increased autophagy. We also observed a significant increase in the axon growth marker GAP43 on day 3 in the TG-TBI group (Figure 9, B and C), indicating that HSPB2 and Akt/mTOR signaling promoted neuro-regeneration. However, the pro-autophagic effect of HSPB2 may be independent of Akt/mTOR. Following CQ administration, Akt and mTOR activation were further enhanced in both the WT and TG groups (Figure 9, D and E), likely due to the compensatory mTOR-activating effect to balance SQSTM1 accumulation (44). Interestingly, Akt/mTOR activation was more robust in the TG group than the WT group during CQ administration (Figure 9, D and E). These findings suggest that the pro-autophagic effects of HSPB2 are independent of the Akt/mTOR pathway. However, the increase in GAP43 expression owing to HSPB2 overexpression was reversed following CQ administration (Figure 9, D and E), implying that autophagy inhibition attenuated HSPB2’s pro-regenerative effects. Furthermore, this result indicated that the effects of HSPB2 on neuro-recovery were not primarily achieved through the Akt/mTOR signaling pathway but primarily through autophagy promotion.

### HSPB2 participates in autophagy by forming the HSPB2/BAG3/SQSTM1 complex.

**Figure 10:**
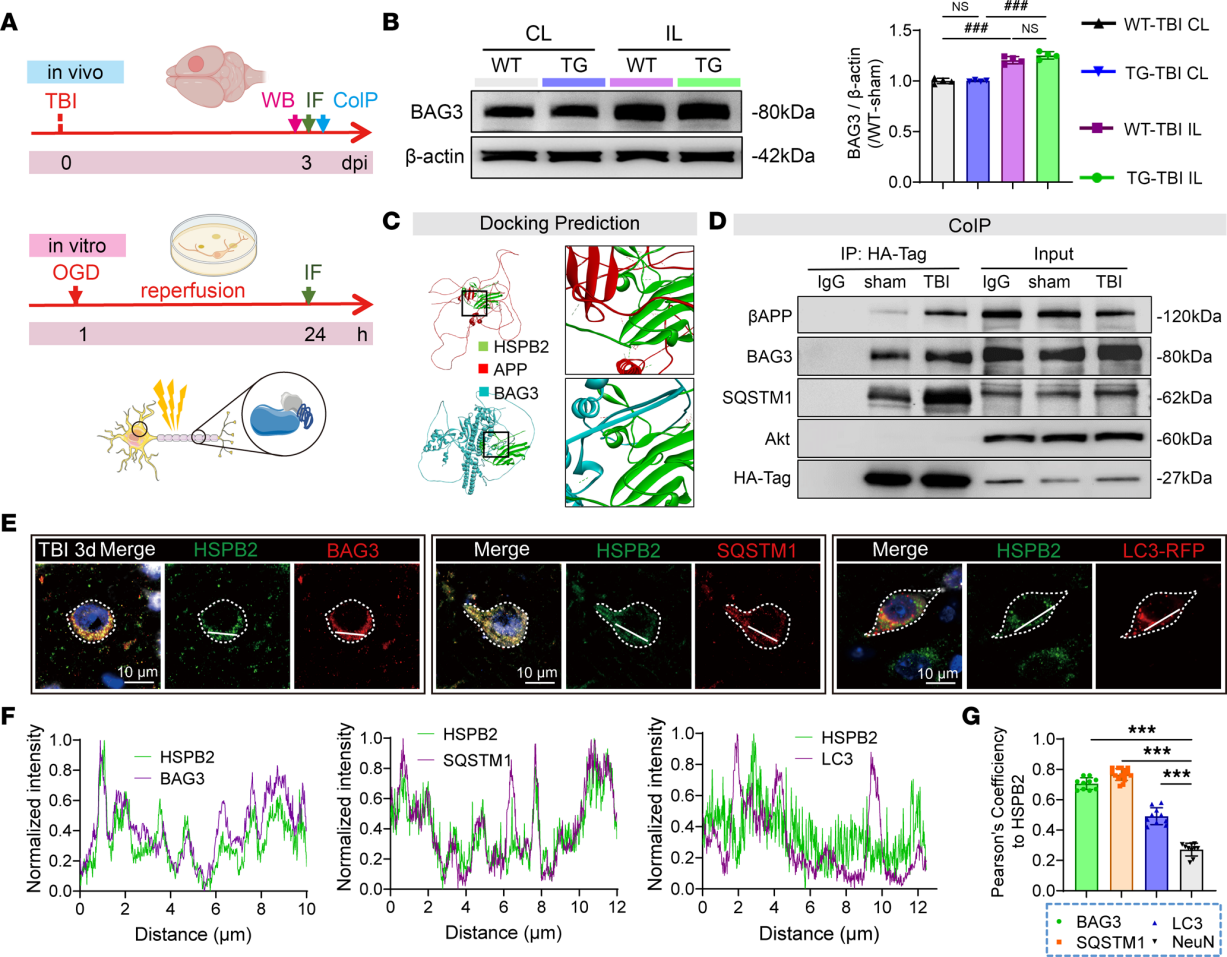
HSPB2 participates in forming an HSPB2/BAG3/SQSTM1 complex. (**A**) Experimental design to assess HSPB2’s interaction. (**B**) Illustration and quantitative analysis of BAG3 in CTX at 3 days postinjury. *n* = 4, analyzed using 1-way ANOVA and post hoc Bonferroni’s test. (**C**) Illustration of predicted protein docking between HSPB2, βAPP, and BAG3; box indicates zoomed area, and dotted line represents intermolecular hydrogen bond. (**D**) Illustration of co-immunoprecipitation of anti–HA-Tag. IgG group represents mouse IgG negative control. (**E**) Illustration of cell body colocalization of HSPB2 with BAG3, SQSTM1, and LC3 at 3 days after TBI. Blue: DAPI; gray: NeuN. Line indicates region of plot profile; scale bar = 10 μm. (**F**) Plot profile of fluorescence intensities relative to HSPB2 and BAG3/SQSTM1/LC3, demonstrating cytosolic HSPB2 and BAG3/SQSTM1/LC3 colocalization; fluorescence intensities of each protein were normalized as 0 to 1. (**G**) Quantitative analysis of Pearson’s correlation coefficient for HSPB2 with BAG3, SQSTM1, LC3 and NeuN. *n* = 10–15, analyzed using 1-way ANOVA and post hoc Bonferroni’s test. *TG versus WT, ^#^IL versus CL. ***/^###^: *P* < 0.001.

Accumulating evidence indicates that chaperones remove misfolded or aggregated proteins via macroautophagy in cooperation with co-chaperones (45). To further investigate the molecular mechanism by which HSPB2 regulates autophagy (Figure 10A), we initially searched human proteomics databases to identify its interacting proteins. HSPB2 reportedly binds to BAG3, an important co-chaperone involved in selective autophagy (29). We found BAG3 protein levels to be upregulated following TBI (Figure 10B); however, HSPB2 did not further upregulate BAG3 (Figure 10B). Nevertheless, interactome data suggest that HSPB2 protein interacts with selective autophagy-related BAG3 and βAPP (46). We subsequently performed docking prediction using the protein structures predicted by AlphaFold. The docking prediction revealed the following: 1) HSPB2 might form structures with BAG3 and βAPP through several hydrogen bonds, 2) the interface areas between the binding partners are extensive, and 3) the free energies of binding between the binding partners are strong (Figure 10C and Supplemental Table 2). These results support the biochemical basis of these interactions in the CNS. We subsequently examined HSPB2’s interaction with BAG3, βAPP, and other autophagy mediators (Figure 10A). Co-immunoprecipitation verified that HSPB2 interacted with BAG3, SQSTM1, and βAPP, but not with Akt (Figure 10D). In addition, the interaction between HSPB2 and βAPP increased following TBI (Figure 10D), which may be attributed to the aberrant accumulation and misfolding of βAPP.

Next, we assessed the interaction of HSPB2 with BAG3 and SQSTM1 by in vivo immunostaining. We found that HSPB2 colocalized with BAG3, the cargo protein SQSTM1, and the autophagosome membrane protein LC3 in AAV-LC3–injected WT mice (Figure 10E). Plot profiles demonstrated significant colocalization of HSPB2 with BAG3 and SQSTM1 and weaker colocalization with LC3 in the neuronal cell bodies (Figure 10F). Pearson’s colocalization analysis revealed that HSPB2 significantly colocalized with BAG3, SQSTM1, and LC3, displaying a greater coefficient than the neuronal marker NeuN (Figure 10G). Moreover, the colocalization of HA-Tag–tagged HSPB2 demonstrated the interactions of HSPB2 with BAG3 and autophagic structures in TG mice (Supplemental Figure 9, A–C).

**Figure 11:**
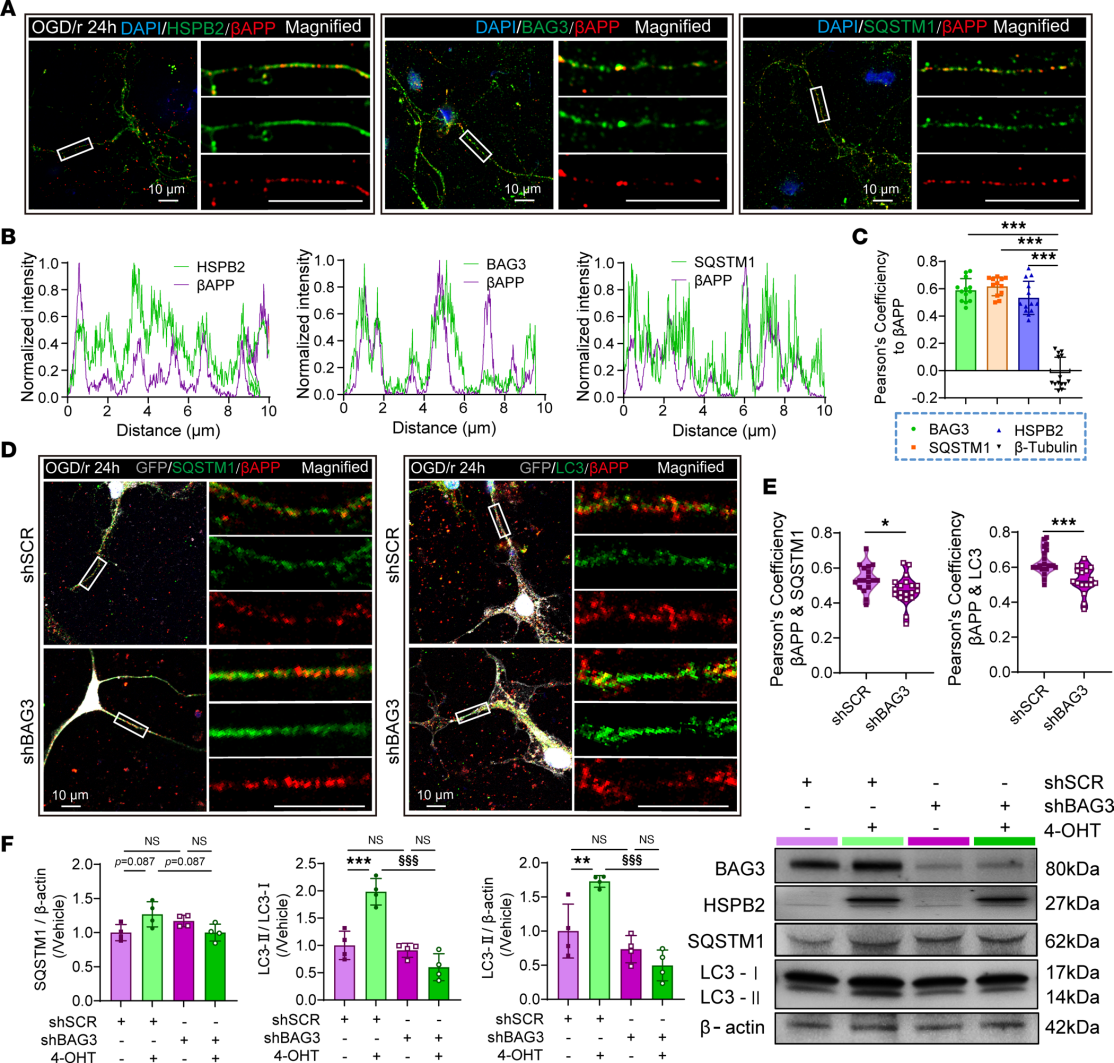
HSPB2/BAG3/SQSTM1 complex might participate in the autophagic clearance of axonal βAPP. (**A**) Illustration of axonal colocalization of βAPP with HSPB2, BAG3, and SQSTM1 in primary neuron culture after OGD. Box indicates magnified area; scale bar = 10 μm. (**B**) Plot profile of fluorescence intensities relative to βAPP and HSPB2/BAG3/SQSTM1, demonstrating axonal βAPP and HSPB2/BAG3/SQSTM1 colocalization. (**C**) Quantitative analysis of Pearson’s correlation coefficient for βAPP with HSPB2, BAG3, SQSTM1, and β-tubulin. *n* = 10–15, analyzed using 1-way ANOVA and post hoc Bonferroni’s test. (**D**) Illustration of axonal colocalization of βAPP with SQSTM1 and LC3 in primary neuron culture after OGD with or without BAG3 silencing, box indicates magnified area; scale bar = 10 μm. (**E**) Quantitative analysis of Pearson’s correlation coefficient for βAPP with SQSTM1 and LC3 with or without BAG3 silencing. *n* = 17–22, analyzed using unpaired 2-tailed Student’s *t* test. (**F**) Quantitative analysis and illustration of BAG3, HSPB2, SQSTM1, and LC3 protein 24 hours after OGD with or without BAG3 silencing or HSPB2 overexpression. *n* = 4, analyzed using 1-way ANOVA and post hoc Bonferroni’s test. 4-OHT, 4-hydroxytamoxifen. Light purple: shSCR; dark purple: shBAG3; light green: shSCR+4-OHT; dark green: shBAG3+4-OHT. ^§^shBAG3 versus shSCR, or as indicated. *: *P* < 0.05, **: *P* < 0.01, ***/^§§§^: *P <* 0.001.

To better observe and locate axonal βAPP, we used a primary neuronal culture in the OGD/r model to induce axonal injury and βAPP accumulation (Figure 10A, in vitro). In the in vitro model, HSPB2 colocalized with BAG3, SQSTM1, and LC3 not only in the cell body but also in the axons (Supplemental Figure 9, D–F). Regarding βAPP, we observed that its density significantly increased in the axons, tending to accumulate at the absent sites of β-tubulin^+^ neurofilament following OGD/r, potentially due to axonal transport malfunction (Supplemental Figure 10, A–D). Moreover, axonal βAPP colocalized with HSPB2, BAG3, and the autophagic cargo protein SQSTM1(Figure 11, A–C), suggesting that HSPB2 links βAPP and selective autophagy.

To verify the role of HSPB2/BAG3/SQSTM1 complex in inducing autophagy, we designed further in vitro experiments using the RNA-silencing strategy targeting BAG3 (Supplemental Figure 10E). BAG3 silencing using lentivirus vector–mediated shRNA (LV-shBAG3) significantly decreased BAG3 protein expression by approximately 85% compared with the nontargeted control (scrambled shRNA, shSCR) (Figure 11F and Supplemental Figure 10, F and H). βAPP’s density among the axons of primary neurons transfected with shBAG3 increased remarkably, possibly due to clearance failure (Supplemental Figure 10G). Moreover, shBAG3 significantly weakened the axonal colocalization of βAPP with SQSTM1 and LC3 without substantially affecting its colocalization with HSPB2 (Figure 11, D and E, and Supplemental Figure 10G), indicating that BAG3 plays an important role in inducing βAPP’s autophagy. Furthermore, 4-OHT was administered to induce HSPB2 overexpression in vitro (Supplemental Figure 10E), which reproduced the autophagy-promoting effects of HSPB2 (Figure 11F and Supplemental Figure 10H). More importantly, BAG3 silencing reversed the increase in LC3-II and SQSTM1 induced by HSPB2 overexpression, suggesting that HSPB2’s autophagy-promoting effects relied on BAG3 (Figure 11F).

Taken together, our findings revealed that HSPB2 forms an HSPB2/BAG3/SQSTM1 complex to facilitate autophagy, which may participate in the selective autophagy of aberrantly accumulated βAPP.

### HSPB2 reduces axonal βAPP and Aβ aggregations partially through autophagy in the acute stage.

**Figure 12:**
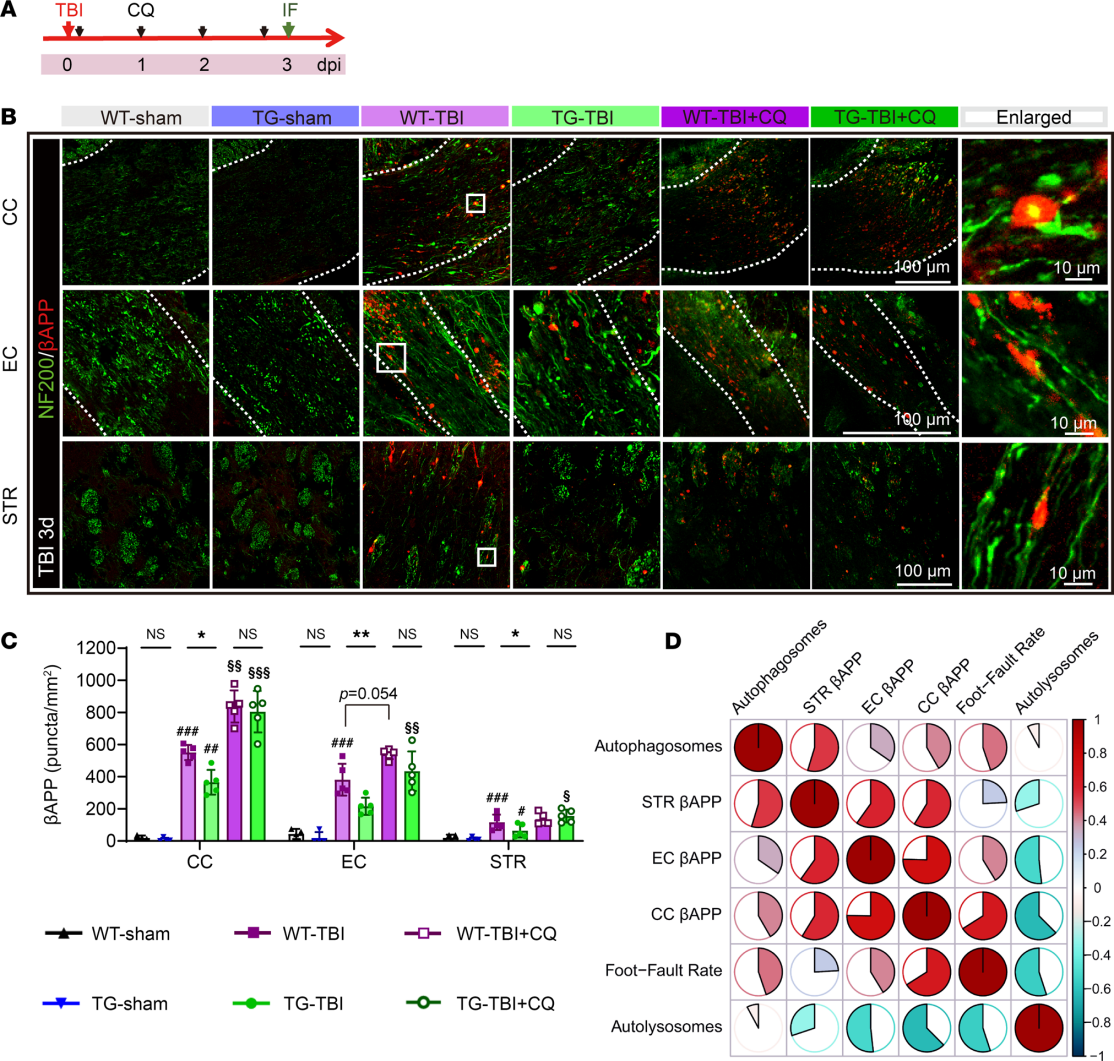
HSPB2 reduces axonal βAPP aggregation partially through autophagy at the acute stage. (**A**) Experimental design for aggregated protein assessment. (**B**) Illustration of βAPP deposition and axonal degeneration/retraction ball formation in CC, EC, and STR with or without CQ at 3 days after injury. Dotted regions indicate ROI; boxes indicate the enlarged areas. (**C**) Quantitative analysis of βAPP plaque density. *n* = 4–6, analyzed using 1-way ANOVA and post hoc Bonferroni’s test. (**D**) Correlation matrix among the number of autophagosomes, autolysosomes, βAPP plaque density in CC, EC, and STR and foot fault rate in the grid-walking test at 3 days following TBI. Color depth and fan size represent the Spearman’s *r* value. *n* = 5, analyzed using Spearman’s correlation test. *TG versus WT, ^#^TBI versus sham, ^§^CQ versus non-CQ, or as indicated. */^#^/^§^: *P* < 0.05, **/^##^/^§§^: *P* < 0.01, ^###^/^§§§^: *P* < 0.001.

Next, we examined whether HSPB2 could reduce βAPP deposition through autophagy during the acute stage (Figure 12A and Supplemental Figure 11A). In the acute stage of TBI, βAPP deposition occurs at the fractured axon tips owing to axonal transport malfunction, which is a sensitive marker of diffuse axon injury (47). Spherical βAPP plaques were observed at the NF200 (neurofilament marker) fracture sites, indicating axonal degeneration and retraction ball formation (Figure 12B). In white matter–rich areas such as the CC, EC, and STR, βAPP deposition density significantly decreased with HSPB2 overexpression 3 days post-TBI (Figure 12, B and C).

Previous studies suggest that βAPP deposition following TBI may transform into Aβ aggregation, potentially causing long-term neuronal death, cognitive impairment, and the development of TBI into AD (5, 48). We subsequently investigated aberrant Aβ aggregation following TBI. Three days post-TBI, Aβ mainly accumulated in the neuronal cytoplasm at the CTX and HIP, and to a lesser extent in the white matter–rich areas CC, CTX, and STR, where Aβ was scattered outside the cell bodies or enwrapped in non-neuronal cells (Supplemental Figure 11B). Interestingly, HSPB2 significantly decreased Aβ aggregation in the CTX, CC, and EC; however, no significant differences were observed in the HIP and STR (Supplemental Figure 11, B and C). These results suggested that HSPB2 may be involved in clearing aberrantly aggregated βAPP and reducing its proteolytic cleavage product Aβ.

When autophagy was blocked by CQ, βAPP accumulation worsened, and no significant difference was observed between the TG and WT groups (Figure 12, B and C). A similar trend was observed in Aβ deposition in the CTX, CC, EC, and STR (Supplemental Figure 11, B and C), suggesting that autolysosome dysfunction might induce Aβ aggregation, consistent with previous findings (49).

Correlation analysis revealed that βAPP density negatively correlated with the AAV-LC3–labeled autolysosomes and positively correlated with the foot fault rate on day 3 (Figure 12D and Supplemental Table 3). Thus, HSPB2 promoted the clearance of axonal damage–related protein βAPP through autophagy in the acute stage, potentially improving neurofunction.

Collectively, our data demonstrate that HSPB2 reduced axonal βAPP and Aβ aggregations at the acute stage, at least partially through autophagy, which may be achieved by guiding aberrantly accumulated βAPP degradation through selective autophagy.

### HSPB2 promotes long-term neuro-recovery through acute-stage neuronal autophagy.

**Figure 13:**
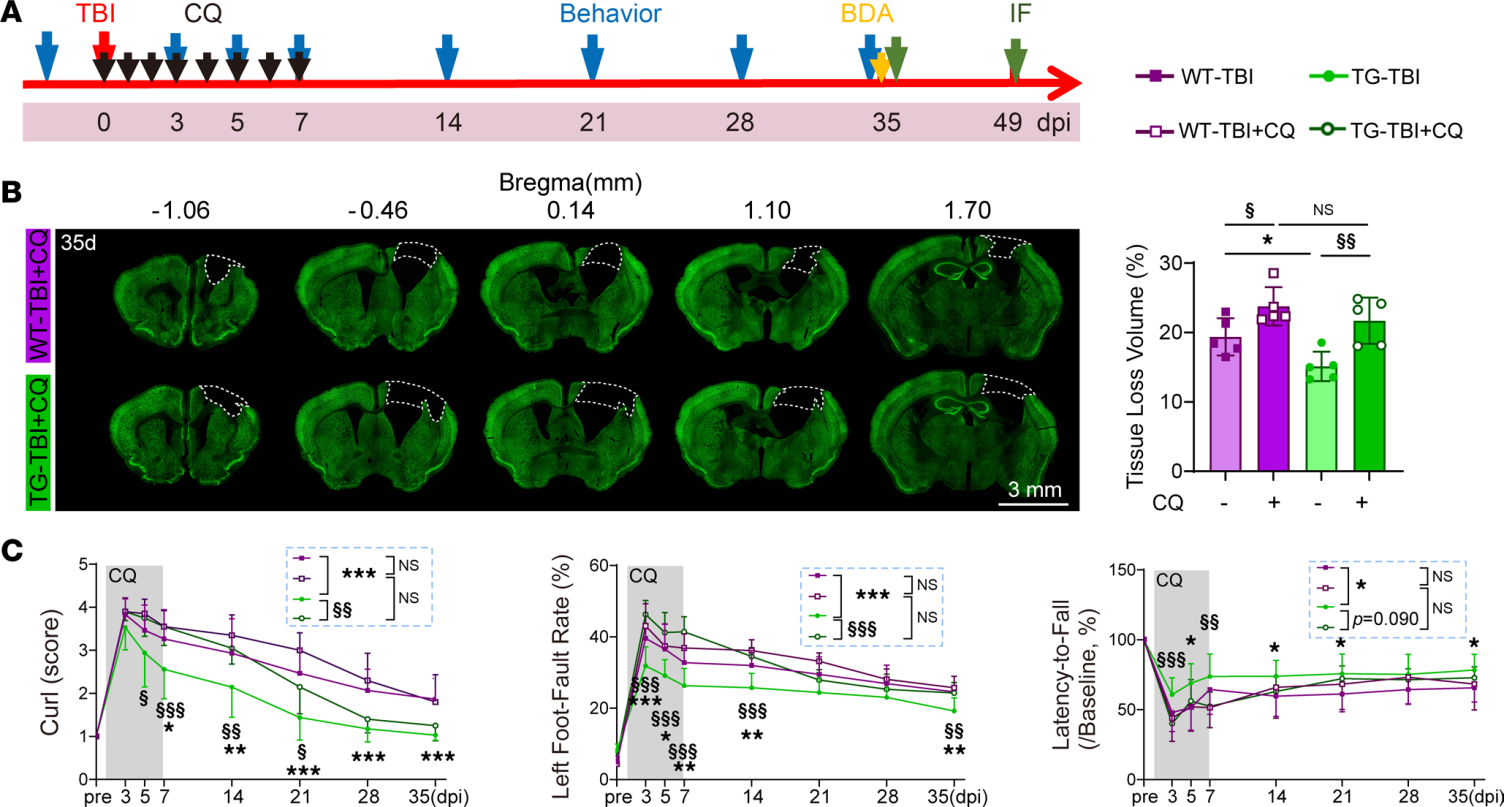
HSPB2 promotes long-term neuro-recovery through acute-stage neuronal autophagy. (**A**) Experimental design for autophagy inhibition. (**B**) Illustration and quantitative analysis of tissue loss volume at 35 days postinjury with CQ treatment. Dotted boxes indicate the loss area; scale bar = 3 mm; *n* = 5, analyzed using 1-way ANOVA and post hoc Bonferroni’s test. (**C**) Quantitative analysis of body curl, grid-walking test, and rotarod test from 3 to 35 days postinjury with CQ treatment. *n* = 15 WT-TBI, 10 WT-TBI+CQ, 17 TG-TBI, and 10 TG-TBI+CQ, analyzed using 2-way ANOVA and post hoc Bonferroni’s test. *TG versus WT, ^§^CQ versus non-CQ, or as indicated. */^§^: *P* < 0.05, **/^§§^: *P* < 0.01, ***/^§§§^: *P* < 0.001.

To investigate whether the pro-recovery effects of HSPB2 resulted from its autophagy-inducing function, we blocked acute-stage autophagy via CQ administration and evaluated long-term functional recovery and axon sprouting (Figure 13A). The duration of CQ administration was extended to 7 days, ensuring its effectiveness in inhibiting acute-stage autophagy, as demonstrated earlier (Figure 7E). Notably, acute-stage CQ inhibition significantly reversed HSPB2’s effects on improving long-term tissue repair (Figure 13B). Additionally, sensorimotor behavior recovery promoted by HSPB2 was reversed without affecting body weight (Figure 13C and Supplemental Figure 12A), indicating acute-stage autophagy plays a vital role in long-term neuro-repair. To exclude the possibility that CQ and HSPB2 also influenced long-term autophagic levels, we assessed the autophagic levels on day 35 with or without CQ and with or without HSPB2 overexpression. On day 35, no difference was observed in the proportion of LC3^+^ neurons between the TBI and sham groups and between those with and without CQ (Supplemental Figure 12B), which was consistent with a previous study indicating autophagy returned to normal within 7 days (50). These results suggested that HSPB2’s long-term beneficial effects were mediated by acute-stage autophagy.

**Figure 14:**
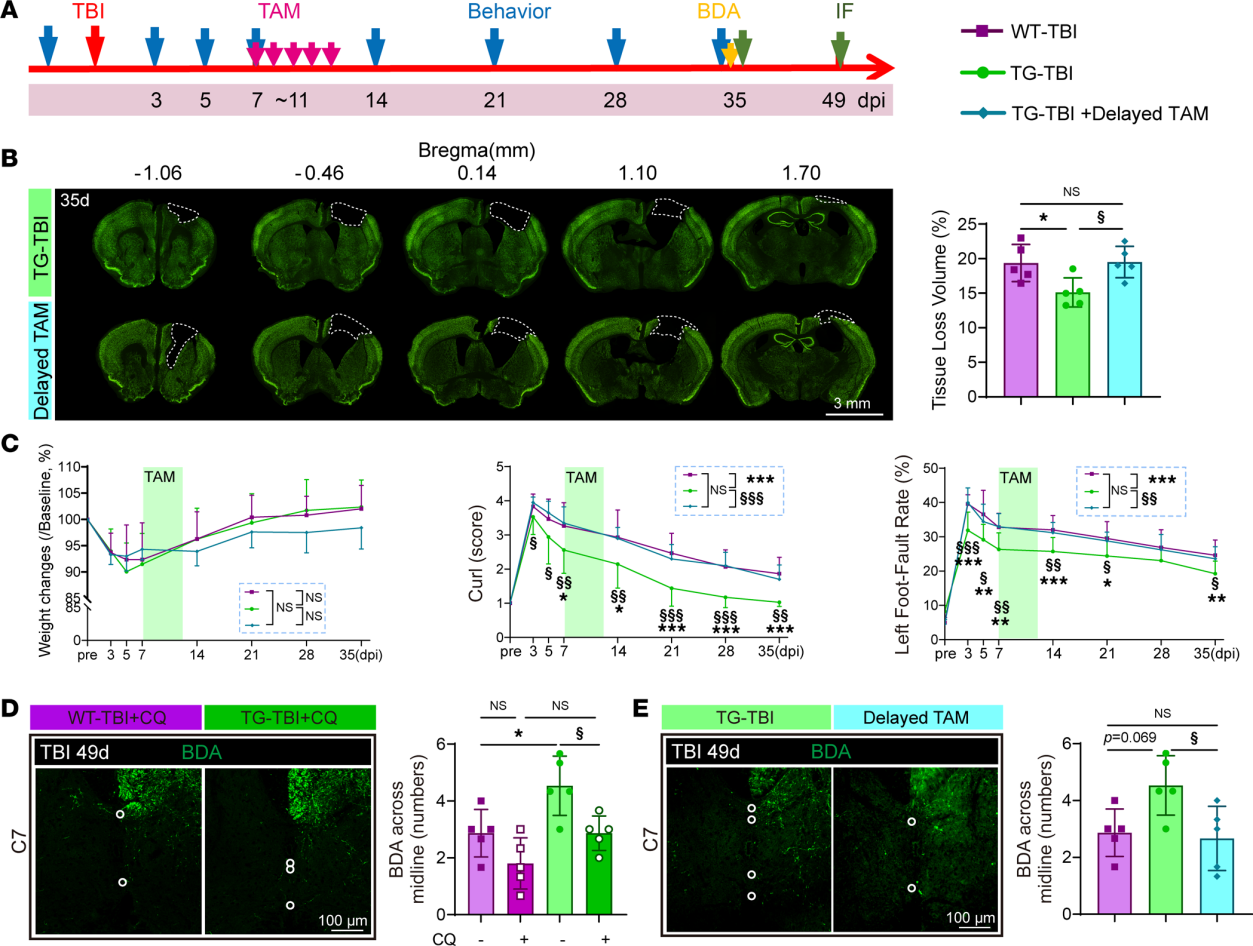
Delayed HSPB2 induction does not exhibit neuro-recovery functions. (**A**) Experimental design for delayed tamoxifen induction. (**B**) Illustration and quantitative analysis of tissue loss volume and area at 35 days postinjury with delayed tamoxifen induction (delayed TAM); dotted boxes indicate the loss area; scale bar = 3 mm. *n* = 5, analyzed using 1-way ANOVA and post hoc Bonferroni’s test. (**C**) Quantitative analysis of weight change, curl test, and grid-walking test from 3 to 35 days postinjury with delayed TAM. *n* = 15 WT-TBI, 17 TG-TBI, and 10 TG-TBI+Delayed TAM, analyzed using 2-way ANOVA and post hoc Bonferroni’s test. (**D** and **E**) Illustration and quantification of BDA^+^ fiber crossing the midline in CC and FN at 49 days postinjury with CQ (**D**) or delayed TAM (**E**), scale bar = 100 μm. *n* = 5, analyzed using 1-way ANOVA and post hoc Bonferroni’s test. *TG vs. WT, ^§^delayed vs. normal TAM, or as indicated. */^§^: *P* < 0.05, **/^§§^: *P* < 0.01, ***/^§§§^: *P* < 0.001.

Furthermore, to exclude other potential roles of HSPB2 in supporting long-term neuro-repair, HSPB2 induction was delayed to 7 days postinjury (Figure 14A). Delayed HSPB2 induction (TG-TBI + delayed TAM, or abbreviated as delayed TAM) failed to exhibit beneficial effects in alleviating tissue loss in the TG-TBI group (Figure 14B). The recovery of long-term behaviors was also impaired by delayed HSPB2 induction (Figure 14C). Furthermore, the compensatory axon sprouting of the cortical neurons in C7 on day 49 was diminished by acute-stage autophagic inhibition and delayed HSPB2 induction (Figure 14, D and E, and Supplemental Figure 12, C and D), highlighting the importance of acute-stage autophagy in long-term neural remodeling. Consequently, HSPB2’s beneficial effects of improving neuro-recovery and neural remodeling were associated with acute-stage activation of neuronal autophagy.

In summary, our study demonstrated that HSPB2 participates in autophagy during the acute stage, promoting neural regeneration and rescuing long-term sensorimotor functions. It also suggests the potential of HSPB2 in forming the HSPB2/BAG3/SQSTM1 complex to guide the clearance of erroneously accumulated axonal proteins, such as βAPP, for degradation through selective autophagy.

## Discussion

To date, studies on HSPB2 in the CNS are limited. Consistent with previous studies, our findings verified the low expression of HSPB2 in neurons within the CNS and further revealed its upregulation in response to brain injury. The increase in HSPB2 expression in primary neuron cultures subjected to OGD/r suggested its involvement in acute neuronal injuries beyond TBI, including stroke and other acute CNS injuries. Additionally, HSPB2 elevation has also been observed in aging and dementia (51), which is considered a resistance mechanism against protein aggregation (52). Notably, the increased HSPB2 level was significant in cortical neuron layer 5, which contains pyramidal neurons with long projections, implying a vital role of HSPB2 in axonal injury and regeneration.

Our study supports the hypothesis that HSPB2 improves the autophagic flux by binding BAG3, similar to HSPB8 (29). A previous study indicated that HSPB3 negatively regulates the association of HSPB2 with BAG3 (29). However, under the HSPB2 overexpression conditions, the HSPB3 level did not upregulate in a compensatory manner, ensuring HSPB2’s combination with BAG3. HSPB2 is also critical for mTOR hyperactivity (53). In our study, HSPB2 showed no direct interaction with Akt, unlike its family member HSPB1, which interacts directly with Akt via chaperoning (54). Nevertheless, augmented activation of Akt/mTOR was observed under HSPB2 overexpression conditions, suggesting that involvement of HSPB2 in mechanisms promoting Akt/mTOR, such as the compensatory effect of balancing autophagy intensity. Notably, Akt/mTOR signaling participates in neuronal survival, axon regeneration, and synapse formation (55). Our findings demonstrated an increase in GAP43 expression in the TG-TBI mice, suggesting that HSPB2 may facilitate axon growth partly through Akt/mTOR signaling.

Autophagy plays a crucial role in CNS diseases. Although controversies have arisen regarding the double-edged effects of autophagy in neurotrauma, most studies have focused on acute stages (56, 57). Some controversies stem from neglecting the detection of the autophagic flux, which obscure the actual condition of autophagy. Research has also shown that TBI causes lysosomal membrane damage, leading to autophagy inhibition, which may result in the accumulation of both LC3-II and SQSTM1 (43). To comprehensively reveal the autophagic flux, we employed a GFP-RFP–tagged LC3 approach combined with a CQ inhibitor strategy to validate HSPB2’s promotion effects on the autophagic flux. Our results validate that HSPB2 enhances autophagic flux without impairing lysosomal functions. However, the lysosomal function following TBI remains to be assessed. TBI-induced lysosomal membrane damage leads to autophagy inhibition (43), and restoration of lysosomal function is a potential strategy for improving autophagy following TBI. Further investigations are warranted to determine whether HSPB2 restores lysosomal function. Current research suggests that autophagy exhibits regeneration and remodeling potential following injury (58, 59). Our study reveals that acute-stage autophagy is critical for long-term neuro-regeneration and remodeling. However, the mechanism by which acute-stage autophagy influences long-term regeneration remains uncleared. Based on our knowledge, the association between acute-stage autophagy and long-term regeneration can be inferred in 2 ways: autophagy may recycle more energy and materials for neurons and axons for regeneration, or it may facilitate the clearance of injury-related aberrantly accumulated organelles and proteins, thereby supporting the spontaneous repair of neurons and axons.

BAG3 is considered crucial for selective autophagy, with significance in CNS diseases, particularly misfolded protein–related diseases such as AD and Parkinson disease (60, 61). BAG3 responds to the cleavage product of the Aβ protein precursor sAβPPα (61). Our study suggests a potential link between HSPB2, βAPP, and co-chaperone BAG3 in selective autophagy. In addition, in vitro RNA silencing revealed that BAG3 plays an important role in HSPB2-mediated autophagy and βAPP clearance. BAG3 silencing did not affect HSPB2’s colocalization with βAPP. Nevertheless, it diminished βAPP’s colocalization with autophagic proteins, which suggests that BAG3 is not required for HSPB2’s combining with βAPP, but it may be crucial for coordinating HSPB2/βAPP complex with autophagy to facilitate βAPP’s autophagic clearance. However, the current study did not establish the causative effect of HSPB2 in promoting βAPP degradation via BAG3-mediated selective autophagy. Future research should explore the underlying mechanisms by which HSPB2 regulates βAPP levels, potentially involving 2 pathways: facilitating βAPP degradation or attenuating axonal damage to reduce βAPP accumulation. Given the dual effects of autophagy on neuronal injury, strategies targeting selective autophagy hold promise in terms of both efficacy and safety. Aβ is known to induce long-term neuronal death and cognitive impairment following TBI (5), and a recent study has attributed Aβ accumulation to defective autolysosomal acidification (49). In the present study, improved autophagic flux resulted in a reduction in βAPP and Aβ accumulation, highlighting the benefits of HSPB2 and autophagy. Additionally, HSPB2’s involvement in selective autophagy may extend beyond βAPP. HSPB8 reportedly induces another form of selective autophagy, mitophagy, targeting mitochondria following OGD/r injury (28), suggesting that HSPB2 may also induce mitophagy, given its shared co-chaperone BAG3.

Our findings indicate that endogenous HSPB2 is mainly expressed in neurons following TBI, with notable upregulation in the astrocytes and microglia. The autophagy of astrocytes and microglia has been demonstrated to play a crucial role in CNS injury (20, 62). Extensive evidence highlights the significant contribution of the astrocytes and microglia to neuroinflammation, which potentially affects neuro-regeneration and axonal regeneration (6, 63). Further investigation is warranted to determine whether HSPB2 is involved in the regulation of autophagy in the astrocytes and microglia. Additionally, exploring the interplay between autophagy, neuroinflammation, and neuro-regeneration warrants continued attention.

HSPB2’s capacity to induce autophagy ensures increased vitality of spared circuits in compensatory remodeling. However, it has been inadvertently suggested that HSPB2 impairs nerve electrical conduction under normal conditions, as evidenced by a decrease of the N1/N2 amplitude. Recent studies have suggested that excessive or imbalanced autophagy can lead to synaptic deficits (64). This observation serves as a reminder of the dual effects of autophagy on CNS diseases and emphasizes the importance of balanced autophagy in maintaining homeostasis. However, further studies are warranted to elucidate the role of autophagy in neuronal homeostasis.

In conclusion, our study identified the beneficial effects of HSPB2 on long-term recovery and neuro-regeneration following TBI via acute-stage autophagy. As a sHSP, rational drug design involving HSPB2 could pave the way for future therapies addressing a range of CNS diseases, including TBI.

## Methods

### Statistics.

Data were analyzed using SPSS Statistics 20 and GraphPad Prism 8. All values are presented as mean ± SD. Normality of data was assessed using the Kolmogorov-Smirnov test. Multiple comparisons were analyzed using 1-way ANOVA and post hoc Bonferroni’s test. Repeated measures data were analyzed using 1-way ANOVA with repeated measures and multivariate analysis of variance. When comparing multivariate data, 2-way ANOVA and post hoc Bonferroni’s test was used. When comparing 2 groups, unpaired 2-tailed Student’s *t* test was used. For paired data, paired 2-tailed Student’s *t* test was used. The correlation analysis and correlation matrix were analyzed using Spearman’s correlation and visualized in R version 3.6.3. A *P* value less than 0.05 was considered statistically significant. For the specific statistical analysis, please see Supplemental Table 4.

### Study approval.

All animal procedures were approved by Experimental Animal Ethics Committee, School of Basic Medicine, Fudan University (approval number: 2018-JS-003). All efforts were made to minimize animal suffering and the number of animals used. For patients, written informed consent was obtained. Patients’ tissue was obtained and used in a manner compliant with the Declaration of Helsinki, and all procedures were approved by the China Human Genetic Resources Management Office of the Huashan Hospital, approval number: GKYBSZ (year 2017) No. 256.

### Data availability.

All relevant data are available in the Supporting Data Values XLS file. See complete unedited blots in the supplemental material.

For other methods, please see Supplemental Methods.

## Author contributions

YG designed the study. YH, SM, BW, HS, YW, JX, ZS, YL, and XJ performed the experiments. YH, JX, and JL analyzed the data. YG, ZXX, GW, and JH validated the data. YH wrote the manuscript. YG and ZXX critically edited the manuscript.

## Supplementary Material

Supplemental data

Supplemental table 1

Supplemental table 3

Supplemental table 4

Supplemental video 1

Supplemental video 2

Supporting data values

## Version 1 08/22/2023

Electronic publication
