# Supplementary material for: HSPB2 facilitates neural regeneration through autophagy for sensorimotor recovery after traumatic brain injury
Source: JCI Insight. 2023 Aug 22;8(16):e168919. doi: 10.1172/jci.insight.168919 (PMC10543718; doi:10.1172/jci.insight.168919)
Supplement: Supplemental data [file jciinsight-8-168919-s079.pdf]

HSPB2 Facilitates Neural Regeneration for Sensorimotor Recovery after Traumatic Brain Injury through Autophagy

Yichen Huang [1], Shan Meng [1], Biwu Wu [2], Hong Shi [3], Yana Wang [1], Jiakun Xiang [1], Jiaying Li [1], Ziyu Shi [1], Gang Wu [2], Yanchen Lyu [1], Xu Jia [1], Jin Hu [2], * Zhi-Xiang Xu [1] and * Yanqin Gao [1]

[1] State Key Laboratory of Medical Neurobiology, MOE Frontiers Center for Brain Science, and Institutes of Brain Science, Fudan University, Shanghai, China;

[2] Department of Neurosurgery, Huashan hospital, Fudan University, Shanghai, China

[3] Department of Anesthesiology, Shanghai Pulmonary Hospital, School of Medicine, Tongji University, Shanghai, China;

*Corresponding Author

Yanqin Gao

State Key Laboratory of Medical Neurobiology, Fudan University, Shanghai 200032, China.

Email: yqgao@shmu.edu.cn, Telephone: +8621 54237778

Zhi-Xiang Xu

MOE Frontiers Center for Brain Science, and Institutes of Brain Science, Fudan University, Shanghai 200032, China.

Email: zhixiangxu@fudan.edu.cn

This PDF file includes:

- Supplemental Methods
- Figure S1 to S11
- Table S2
- Legends for Movies S1 to S2
- Supplemental References

Other supporting materials for this manuscript include the following:

Tables S1, 3, 4

Movies S1 to S2

Supporting data

Supplemental Methods

Animals.

C57BL/6J mice were purchased from Shanghai Jie Si Jie Laboratory Animal Co., Ltd. HSPB2 knock-in mice (C57BL/6-*Gt (ROSA)26Sor*$^{em1\ (CAG\text{-}LoxP\text{-}STOP\text{-}LoxP\text{-}HSPB2\text{-}3\times HA\text{-}WPRE\text{-}polyA)}$, *Hspb2* gene ID: CCDS8352, abbreviated as *R26-e(CAG-LSL-Hspb2)*) were made from Shanghai Biomodel Organism Science & Technology Development Co., Ltd., and *Map2-Cre*^{ERT2} mice (C57BL/6-*Map2*$^{em1(2A\text{-}CreERT2)Smoc}$, Cat# NM-KI-00037) were also purchased from Shanghai Biomodel Organism. Neuron-specific HSPB2 overexpression [abbreviated as TG (transgenic)] mice were obtained by crossing the *R26-e(CAG-LSL-Hspb2)* mice and *Map2-Cre*^{ERT2} mice for two generations. The TG mice (genotype: *Rosa26*$^{LSL/LSL}$ × *Map2*$^{CreERT2/wt}$) were viable, fertile, and normal in size, displaying no apparent physical or behavioral abnormalities. To induce gene overexpression, TG mice received intraperitoneal injections of tamoxifen (75 mg/kg daily for 5 days). Hemizygous *R26-e(CAG-LSL-Hspb2)* mice (genotype: *Rosa26*$^{LSL/LSL}$ × *Map2*$^{wt/wt}$) served as age- and sex-matched wild-type (WT) control mice and received the same tamoxifen treatments. Mice were subjected to cortical controlled impact 10 days after tamoxifen treatments. For delayed tamoxifen induction, tamoxifen was administrated 7 days post-impact for 5 days.

Male 9-12 weeks old (25~28g) or female 12-15 weeks (25g~28g) mice were used for the experiment. Mice were housed in a pathogen-free facility with regulated temperature and humidity and a 12-h light/dark cycle. Food and water were provided ad libitum. All animal experiments in the study were approved by the Animal Care and Use Committee of Shanghai Medical College, Fudan University.

Patients

Brain tissues were collected from the damaged regions surgically removed from three patients with cerebral contusion and laceration (Table 1) who underwent emergency neurosurgery operations in Huashan Hospital, Shanghai, China. These tissues were marginal and inevitably excised during surgical removal of hematoma and necrotic tissue, with all precautions taken to minimize damage to healthy tissue. Informed consent was obtained from each patient and family. All patients had severe cerebral contusion and laceration, with no prior history of brain injury or other neurological disorders. Upon removal, the tissue was quickly dehydrated and paraffin-embedded, and sectioned into 10 μm slices for subsequent immunostaining.

Table 1. Detailed information of patients

Patients	Age	Gender	diagnosis	Tissue location	Tissue size (cm)	Admission neurological outcomes (GCS)
1	48	Male	Cerebral contusion and laceration	Left basal ganglia	2*1.5*0.6	10
2	54	Female	Cerebral contusion and laceration	Left basal ganglia	Diameter 1.5	3
3	32	Male	Cerebral contusion and laceration	Right basal ganglia	Diameter 0.3	14

GCS: Glasgow Coma Scale.

TBI model

TBI was induced using a cortical controlled impact (CCI) device (TBI 0310, Precision Systems and Instrumentation) following a previously described procedure(1). In brief, mice were anesthetized with 3 % isoflurane in a 70% N_2/30% O_2 mixture and fixed on stereotaxic apparatus maintaining breathing by animal respirator with 1.5 % isoflurane in 70% N_2/30% O_2 mixture and temperature by warming light. After sterilizing and removing hair, a midline incision was made to expose the skull. An approximate 4-mm craniotomy was performed over the right parietotemporal cortex using a motorized drill. The CCI was centered 2.5 mm lateral to the midline and 0.5 mm anterior to the bregma, conducted with a 3-mm flat-tip impounder. The CCI indexes were set as follows: velocity, 3.5m/s; duration, 150ms; depth, 1.5mm. After the CCI, the scalp incision was carefully sutured, and the mice were placed on a heating pad until recovery from anesthesia. Sham mice underwent all procedures except for CCI.

Primary neuronal culture and oxygen/glucose deprivation (OGD)

Cortical neurons were cultured from E16-18 fetal mice. The isolated cortex was removed and digested with 20 U/ml papain in Dulbecco's modified eagle medium (DMEM) /F12 (Cat# 11320033, Gibco) at 37 °C for 10 min. Dissociated neurons were cultured in Neurobasal media A (Cat# 21103049, Gibco) supplemented with 2% B-27 (Cat# 17504044, Gibco), 1% GlutaMAX (Cat# 35050079, Gibco), and 1% penicillin-

streptomycin (Cat# 15140163, Gibco) at 37 °C and 5% CO_2 incubator. On day 7, neurons were treated with oxygen and glucose deprivation (OGD). In brief, the medium was replaced by glucose-free DMEM/F12 (Cat# 21331020, Gibco), and the culture was transferred to a chamber containing 5% CO_2, 90% N_2, and 5% H_2 at 37 ℃ for 1 hour. Subsequently, the medium was replaced with normal neuron medium and the culture was returned to 37 °C and 5% CO_2 incubator.

To achieve HSPB2 overexpression, primary neurons were cultured from TG (genotype: Rosa26$^{LSL/LSL}$ × MAP2-Cre$^{ERT2/wt}$) fetal mice, and 4-hydroxytamoxifen (4-OHT, A56098, Beijing Wokai Biotechnology Co., Ltd) was added to the medium at the concentration of 2 μM on day 5 followed by subsequent OGD/r operation on day 7.

RNA silencing of Bag3

Lentivirus vectors (pLentai-hU6-shRNA-esEF1A-MataGFP-WPRE-pA) containing shRNA targeting Bag3 (5’-AGGTCTCCTCTGCTCCAATTC-3’, brief as shBAG3) and scrambled shRNA sequence were obtained from Shanghai Taitool Bioscience Co.,Ltd. The scrambled shRNA sequence was used as a control (brief as shSCR). To achieve BAG3 knockdown, the neurons were infected with lentivirus on day 4 by the MOI (multiplicity of infection) = 1 for 24 h, then the virus-containing medium was replaced by normal neurobasal medium.

Immunofluorescence (IF) staining and data analysis

Mice were deeply anesthetized with 1% pentobarbital sodium (50ml/kg) intraperitoneally before being perfused intracardially with ice-cold saline and 4% paraformaldehyde. The brain was harvested and cryoprotected sequentially in 4% paraformaldehyde, 20% sucrose in PB, and 30% sucrose in PB to complete post-fixation and dehydration. 25μm coronal section was obtained using a freezing microtome (HM525NX, Thermo Scientific).

Brain sections were washed with PBS and PBS+0.3% Triton, incubated in PBS+1% Triton X-100 to permeabilize and blocked with 10% goat or donkey serum for 1h. M.O.M. Kit (Cat# BMK-2202, Vector Laboratories) was used to block mouse antibodies. Brain sections were then incubated with primary antibodies overnight at 4℃. After washing, the sections were incubated with secondary antibodies conjugated

with Alexa Fluor-405/488/594/647 (1:1000, Jackson ImmunoResearch Laboratories) for 2 hours at room temperature. Slides were mounted with DAPI-Fluoromount-G (Cat# 0100-20, Southern Biotech). Fluorescence images were captured using a Nikon A1 confocal microscope equipped with NIS-Elements software (Nikon) or with Nikon ECLIPSE Ni-E microscope. Images were processed and analyzed with ImageJ software (Fiji, NIH, https://imagej.net/software/fiji/#publication), and Z-series images were processed and 3D-rendered with Imaris version9.0.1 (Oxford Instruments), as previously described (2).

To assess tissue loss, 10 serial sections with an interval of 11 sections were NeuN immunostained to calculate the NeuN-immunopositive area (mm^2) or volume (mm^3) of tissue loss using ImageJ [area of tissue loss % = (area of contralateral hemisphere - area of the ipsilateral hemisphere) / area of the ipsilateral hemisphere; the volume of tissue loss % =∑ (area of tissue loss) ×25μm× 12 / ∑ area of the ipsilateral hemisphere) ×25μm× 12].

For biotinylated dextran (BDA) staining, a streptavidin blocking kit (SP-2002, Vector Laboratories) was used, followed by immunostaining with streptavidin Alexa Fluor 488/647 (Invitrogen).

Cell counting was performed unbiasedly using ImageJ function Analyze Particles according to consistent parameters, as previously described (2). In brief, positively stained cells were counted from two to three 40x microscopic fields (317.4 μm × 317.4 μm) in the cortex or striatum. A suitable threshold was established to differentiate target signal from background using the Analyze Particles function. The process for β-amyloid precursor protein (β-APP) quantification was consistent with cell counting. For Aβ quantification, fluorescence intensities were measured using ImageJ software by applying threshold adjustment, as described previously (3).

Immunoblotting (Western blotting)

After perfusion, cortical tissue from the peri-injury region (about 4×4×2 mm) of both ipsilateral and contralateral hemispheres was harvested, then homogenized and ultrasonicated with RIPA cell lysis buffer (Cat# 9806, Cell Signaling Technology, Boston, USA), 1 mM PMSF, and phosphatase inhibitor cocktail PhosSTOP (Cat# 4906845001, Roche, Basel, Switzerland) to extract proteins. Western blots were performed using the standard SDS-polyacrylamide gel electrophoresis (PAGE) method. In brief, polyvinylidene difluoride (PVDF) membranes were blocked with 5% nonfat milk for 1 h at room temperature and then

incubated overnight at 4 ˚C with primary antibodies. The membrane was then incubated with secondary antibodies for 1 h at room temperature (1:10,000, Proteintech, Rosemont, IL, USA) and scanned using the Bio-rad ChemiDoc system (Bio-rad, Hercules, CA, USA). ImageJ software was used for gel analyses.

Real-time qPCR

Total RNA was extracted from cortical tissue of the peri-injury region (about 4×4×2 mm) in the ipsilateral hemisphere using TRIzol reagent (Invitrogen, Cat# 15596026). First-stand cDNA was generated using the RevertAid First Strand cDNA Synthesis Kit (Thermo Scientific, Cat# K1622) according to the manufacturers' protocols. The following primers were used for mouse samples:

Hspb1：Forward: ATCCCCTGAGGGCACACTTA-3'
Reverse: 5'-GGAATGGTGATCTCCGCTGAC-3'

Hsbp2：Forward: 5'-CTCTATCCCATGATGGCATCCT-3'
Reverse: 5'-CTCAACTCTGGCTATCTCTTCCT-3'

Hspb3：Forward: 5'-GACCCCAGTGCGTTATCAGG-3'
Reverse: 5'-GGCTTTACTCAGGTCCTCGAT-3'

Hspb5：Forward: 5'-GTTCTTCGGAGAGCACCTGTT-3'
Reverse: 5'-GAGAGTCCGGTGTCAATCCAG-3'

Gapdh : Forward: 5'-CTGCCCAGAACATCATCCCT-3'
Reverse: 5'-TGAAGTCGCAGGAGACAACC-3'

Real-time qPCR was performed using SYBR Green Master Mix (Yeasen, Cat# 11201ES03) on QuantStudio 5 (Thermo Scientific, Cat# A28140). All reactions were performed in triplicates, and the relative amount of mRNA was normalized to GAPDH level.

Behavior tests

Behavioral tests were performed in a blinded manner and analyzed unbiasedly. Sensorimotor functions were measured 3–35 d post-TBI. All behavioral testing was conducted during the light cycle phase. Mice were trained for 3 days before surgeries to reach a close baseline.

The body curl test was used for detecting unilateral lesions(4). Mice were hand-suspended by the tail and rated for degree of torso flexion from vertical towards the contralateral injury side. The degree of body curl was rated as absent (1), mild (2), moderate (3), and severe (4). Normal mice hang vertically without flexion and thus deviate 0° from vertical (rating of 1). Flexion of the torso at 22.5° from vertical was rated 2 (mild); flexion between 22.5° and 45° from vertical was rated 3 (moderate), and flexion of the torso at 45° or more and with/or without grasping of the hindlimbs by forelimbs was given a rating of 4 (severe).

The foot fault test (grid-walking test) was performed as described previously(5) to assess sensorimotor coordination. Mice were placed on an elevated grid surface (30 cm (L) × 35 cm (W) × 31 cm (H)) with a grid opening of 2.25 cm^2 (1.5 cm × 1.5 cm square) and videotaped for 3 min from below the grid. The videotapes were analyzed by a blinded investigator to count the number of total steps and the number of foot faults made by the impaired limbs. Foot faults were determined when the mouse misplaced its left forepaw such that the paw fell through the grid, and expressed as a percentage of total steps. The foot-fault rate = the number of foot faults / the number of total steps.

The rotarod test was performed by a rotating drum (Ugo Basile, Gemonio, Italy). Briefly, mice were forced to run on the rotating drum with speeds starting at 4 rpm and accelerating to 40 rpm within 300 s. Three consecutive trials were conducted for each mouse with an interval of 15 min. The time at which a mouse fell off the drum was recorded as the latency to fall. Data were expressed as mean values from three trials.

The Morris water maze test was carried out 29-34 days following TBI to evaluate spatial cognitive functions as described previously(6). Briefly, in the learning stage (day 29-33), a square platform (11 × 11 cm^2) was submerged 2 cm beneath the water surface in a circular pool (diameter = 109 cm) filled with opaque water. Mice were placed into the pool at one of the four locations and given 60 seconds to locate the hidden platform. At the end of each trial, the mouse was placed on the platform or allowed to stay on the platform for 30 s with prominent spatial cues displayed around the room. The time spent to reach the platform (learning phase) was recorded. Four trials were performed each day with randomly assigned orders. The memory test was performed on day 34. The platform was removed, and a single 60-s probe trial was conducted. Time spent in the target quadrant where the platform was previously located was recorded.

MRI

For in vivo MRI, mice were initially anesthetized with 3% isoflurane and positioned on an animal cradle with a stereotaxic head holder. Anesthesia was maintained between 1% and 1.5% isoflurane throughout the experiment. Respiration and temperature were monitored continuously. MRI was performed with an 11.7T small animal system (Bruker BioSpec 117/16) with a 6-mm 4-channel surface array coil (Bruker BioSpin, Billerica, MA). The DTI data were collected using a spin echo imaging (SE)- DTI imaging sequence using the following setup: TR/TE = 50,00/24 ms, Direction = 30, FOV = 20 ×20 mm, acquisition matrix = 200 × 200, 30 slices with a slice thickness of 0.5 mm, 3 averages, 4 segments, and a b-value = 1,000 s/mm^2. Perfusion-fixed brains at day 49 were left in the skull and imaged ex vivo using the following setup: a SE sequence with 60 directions, TR/TE = 80/19 ms, FOV = 16 ×16 mm, acquisition matrix = 80 × 80, 74 slices with a slice thickness of 0.2 mm, 3 averages, 4 segments, and a b-value = 2,000 s/mm^2. DTI data were analyzed with DSI Studio software (http://dsi-studio.labsolver.org/). ROIs were drawn manually in a blinded manner encompassing the EC (external capsule) and STR (striatum) in the ipsilateral and contralateral hemispheres to determine FA and RD. DEC, FA, and RD maps were generated by DSI Studio software. Tracks across the midline of CC (corpus callosum) were traced and quantified by DSI Studio.

Electrophysiology

Compound action potentials (CAPs) in the external capsule were recorded as previously described(7). Mice were anesthetized with isoflurane and were decapitated to remove the brains rapidly. A 350-µm coronal brain slice was prepared using a vibratome (1200s, Leica). Slices were transferred to artificial cerebrospinal fluid (aCSF) saturated with 95% O_2+5% CO_2 mixture at 32°C for 0.5 h then at room temperature for 1 h for recovery. The aCSF contained 124 mM NaCl, 2.5 mM KCl, 2 mM $CaCl_2$, 1 mM NaH_2PO_4, 24 mM $NaHCO_3$, 1.3 mM $MgSO_4$, and 10 mM D-glucose. A concentric stimulating electrode and a glass microelectrode (5~8 MΩ) were used for stimulating and recording. CAPs were induced by monophase square waves (0.1 ms duration) with a stimulus generator (STG 4002, Multichannel). Evoked signals were amplified by Axoclamp 700B (Molecular Devices) and digitized Axon Digidata 1440A (Molecular Devices).

BDA tracing

Biotinylated dextran amine (BDA, 10,000 MW, Invitrogen, Cat# D1956) was intracerebrally injected to anterograde trace the projection of motor neurons, and label corticospinal tract (CST). Briefly, 2 μl of BDA (10% wt/vol, dissolved in PBS) was stereotactically injected into contralateral cortex (bregma: AP = 0.6 mm, ML = 1.2 mm, and DV = 1.5 mm, M1 zone). After 14 days, mice were sacrificed and coronal sections (25 μm) of the cerebra, cerebellum, and spinal cord were collected. The numbers of BDA fibers across the midline of cerebra corpus callosum (CC) layer (bregma 0mm), medulla oblongata facial nucleus (FN) layer (bregma – 6.36mm), and spinal cord C7 layer were analyzed to assess physiological crossover and compensatory sprouting. The labeled CST naturally crossed to the contralateral side at the corpus callosum in the cerebrum (CC) and the pyramidal tract in the medulla oblongata facial nucleus layer (FN). Compensatory sprouting occurred at the spine cord cervical layer 7 (C7) towards the injured limb.

Calcium fiber photometry recording

In vivo calcium fiber photometry recording was used to assess the cortical remapping of the ascending sensory pathway. For the surgery, mice were anesthetized and the whole contralateral skull, bregma, and lambda were exposed, avoiding exposure to the ipsilateral impact area. AAV2/9-hSyn-GCaMP6m-WPRE-pA (Shanghai Taitool Bioscience, Shanghai, China, 10^{12} V.G./mL, 400 nL) was stereotactically injected into the contralateral cortex [bregma: AP = 0.5 mm, ML = 2.0 mm, and DV = 1.7 mm, forelimb first sensory zone (S1 FL)]. After injection, a ceramic optical fiber [1.7 mm length, O.D. = 160 μm, numerical aperture (NA) = 0.37] was inserted into the injection area (bregma: AP = 0.5 mm, ML = 2.0 mm, and DV = 1.5 mm). Glue, screw, and dental cement were used to secure the optical fiber, then mice were carefully placed on a warm heating-pad until they woke up. Mice were individually housed and allowed to recover for at least two weeks. For recording, the 3-color single-channel fluorescence recording system (Thinkertech, Jiangsu, China) was used. The 470-nm channel was used for recording, while the 405-nm channel served as the control. The light power was adjusted to 30 μV to minimize bleaching. Then the optical fiber was attached to the recording fiber linking to the system. During recording, mice remained awake, and a 100 Hz tactile stimulus was applied to each side of the forepaw for 1 second by a modified device in turn (Video.S2). For each side of the forepaw, 3 trails were recorded. Normalized △F/F was used to evaluate calcium activities. Analysis was

performed on the system's software and Matlab (Mathworks). The reference baseline time period is - 5~0 s, when 0 is the time to deliver the tactile stimulus. The original △F/F was z-scored by each trial (both paws), and the mean △F/F of 0~1 s was calculated to evaluate the calcium activity responding to stimulation.

AAV-LC3 labeling

AAV-mRFP-GFP-LC3 (pHBAAV-CMV-mCherry-EGFP-LC3) was bought from Hanbio (Shanghai, China) and stereotactically injected into the ipsilateral and contralateral cortex (bregma: AP = −1 mm, ML = 2.0 mm, and DV = 1.5 mm) of mouse (2 μl) 14 days before TBI to label autophagic flux. Briefly, after perfusion and dehydration, serial coronal sections (25 μm) were collected and photographed. Z-series images were processed and 3D-rendered using Imaris version 9.0.1 (Oxford Instruments). Since GFP degrades in an acidic environment while RFP does not, yellow puncta (formed out of the overlap between red and green) indicate autophagosomes, while red puncta indicate autolysosomes. About 5 cortical pyramidal neurons labeled were labeled in each hemisphere and randomly selected for further analysis. Pyramidal neurons were identified based on the morphology of long axons and dendrites by weak GFP fluorescence. For each hemisphere, 5 labeled pyramidal neurons were randomly chosen for further rendering and analysis.

CQ application

Chloroquine (CQ, Sigma, Cat# C6628, dissolved in saline solution at 6.25 mg/mL) was intraperitoneal injected (0.625mg/g) 1 hour following TBI and daily till day 3 or 7 to block autophagic flux. Chloroquine accumulates in acidic lysosomes, raising the lysosomal pH, thus inhibiting lysosomal hydrolases and preventing autophagosome fusion and degradation.

Immunoprecipitation

After perfusion, cortex and hippocampus tissues or peri-injury area (about 4×4×2 mm) from the ipsilateral hemisphere were harvested, homogenized, and ultrasonicated with Lysis Buffer for WB/IP Assays (Cat# 20118, Yeasen, Shanghai, China) and 1 mM PMSF to acquire undivided protein. SureBeads Protein G Magnetic Beads (Cat# 1614021, Bio-Rad) were added to either anti-HA-Tag antibody (Mouse, #2367S, Cell Signaling Technology, 1:200) or IgG for 10 minutes, and added to the cleared cell lysates. The mixture was

incubated at room temperature for 1 h. Immunoprecipitates were eluted with 20 mM glycine (pH 2.0) and neutralized with 1 M phosphate buffer (pH 7.4). SDS-loading buffer was added and the mixture was heated at 95 °C for 10 minutes for subsequent immunoblotting analysis.

Immuno-co-localization analysis

The co-localization of the target protein with another protein was assessed by plot profile and Spearman's coefficiency. For the plot profile, a line across the interested region was drawn and the ImageJ *plot profile* function was used to visualize the distribution of the fluorescence intensity of the target protein and the other protein along the same line. For Spearman's coefficiency, ImageJ function *coloc 2* was used to assess the colocalization of the target protein and the other protein in the same region of interest. About 10-15 neurons were randomly selected for analysis.

Docking prediction

Protein structures were obtained from AlphaFold Protein Structure Database (https://alphafold.com/, HSPB2: AF-Q16082-F1, APP: AF-P05067-F1, BAG3: AF-O95817-F1). Docking was predicted by GRAMM-X (http://gramm.compbio.ku.edu/) with the rigid docking method. Interactions of the predicted docking structure were quantified by PDBePISA(https://www.ebi.ac.uk/msd-srv/prot_int/cgi-bin/piserver). The interface area and ΔiG were calculated to assess the stability of the docking structure. Protein structure and intermolecular hydrogen bonds were visualized by Discovery Studio Version 4.5 (BIOVIA).

Antibodies

Table 2. Antibody

Antibody	Host	Company	Category	Dilution
NeuN-Alexa Fluor 488	Rb	Abcam	Ab190195	1:1000
NeuN	Rb	Abcam	Ab104225	1:1000
HSPB2	Rb	SAB	#38402	1:200
Iba1	Goat	Abcam	Ab5076	1:1000
GFAP	Ms	Invitrogen	14-9892-82	1:1000

β-actin	Ms	Proteintech	60008-1-Ig	1:10000
HA-Tag	Rb	CST	#3724	1:1000
HA-Tag	Ms	CST	#2307	1:1000
Synaptophysin	Rb	Abcam	ab32127	1:200
LC3	Rb	Proteintech	14600-1-AP	WB: 1:4000<br>IF: 1:400
SQSTM1	Rb	Proteintech	18420-1-AP	WB: 1:8000<br>IF: 1:200
Akt	Rb	CST	#4691	1:1000
beta Amyloid (CT695) (βAPP)	Rb	Invitrogen	51-2700	1:1000
β-Tubulin (TUBB3) -Alexa Fluor 488	Rat	Abcam	ab195883	1:1000
BAG3	Rb	CST	# 23842	1:1000
Neurofilament heavy polypeptide (NF200)	Rb	Abcam	ab8135	1:1000
beta Amyloid (2C8) (Aβ)	Ms	Invitrogen	MA125493	1:1000
p-Akt（pS473）	Rb	Epitomics	2118-1	1:1000
mTOR	Rb	CST	#2983	1:1000
p-mTOR	Rb	CST	#5536	1:1000
GAP43	Rb	CST	#8945	1:1000

Rb: rabbit; Ms: mouse.

Supplemental Figures

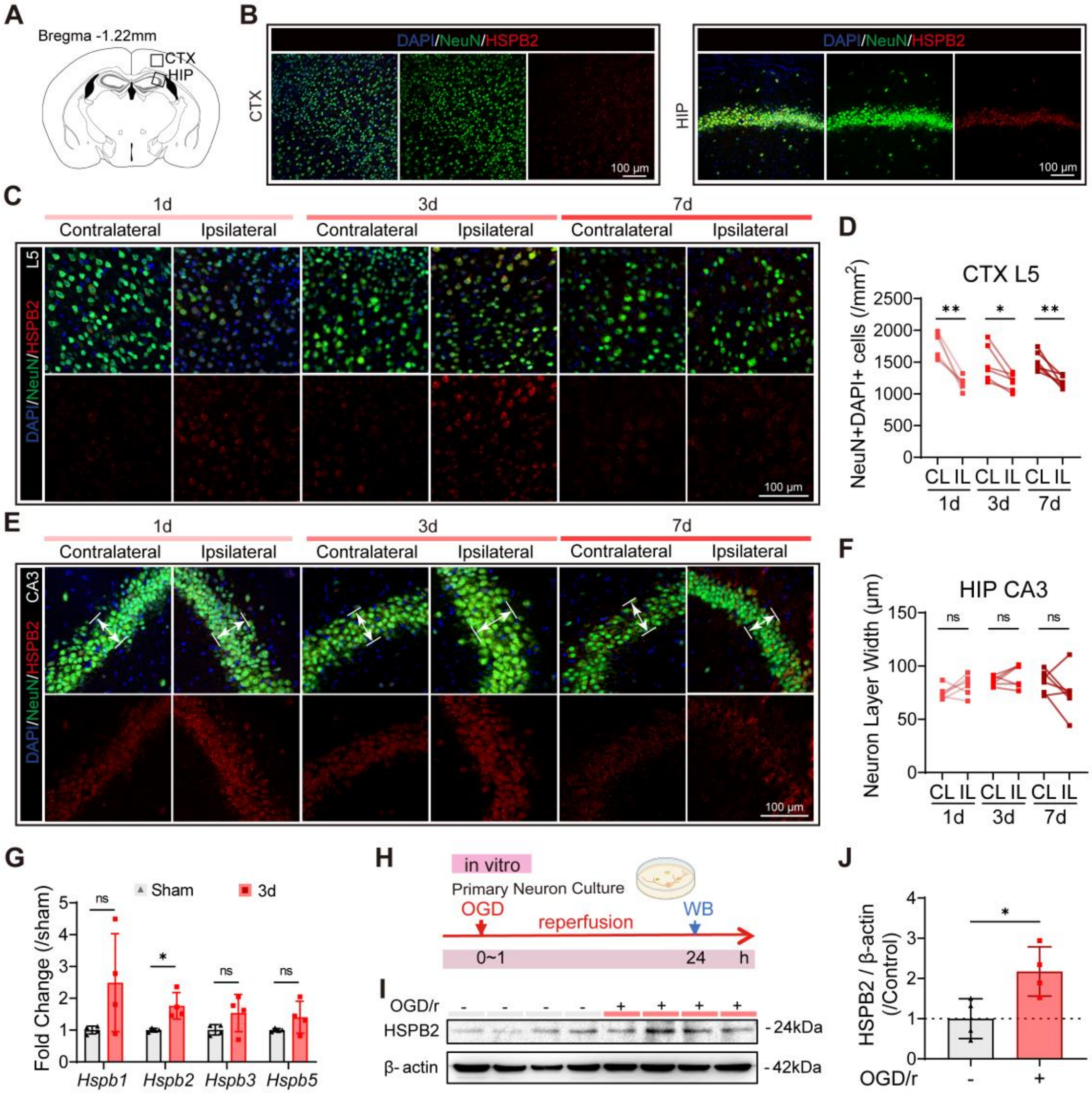

Figure S1 **Physiological and post-TBI HSPB2 expression in neurons.** **A** Region of interest (ROI) of cortex (CTX) and hippocampus (HIP) in the layer of post bregma 1.22mm; **B** Triple-label immunofluorescence of HSPB2(red)/NeuN(green, for neurons)/DAPI(blue) in CTX and HIP of normal brain, scale bar = 100 μm; **C** Representative images of HSPB2 expression in neurons at 1, 3, and 7 days in CTX Layer 5 (L5) after CCI, scale bar = 100 μm; **D** Quantitative analysis of neuron density in CTX neuron layer 5 at different time, n=6, analyzed using paired Student's t-test; **E** Representative images of HSPB2 expression in neurons at 1, 3, and 7 days in HIP CA3 after CCI, arrowed line indicates neuron layer width, scale bar = 100 μm; **F** Quantitative analysis of CA3 neuron layer width at different time, n=6, analyzed using paired Student's t-test; **G** Quantitative analysis of mRNA levels of *Hspb1*, *2*, *3*, and *5* at 3 days post TBI, n=4, analyzed using unpaired Student's t-test; **H** Experimental design of in vitro primary neuron culture OGD model; **I&J** Representative image of western blot and quantitative analysis of lysate HSPB2 relative expression after OGD/r in primary neuron culture, n=4, analyzed using unpaired Student's t-test. *: $p < 0.05$, **: $p < 0.01$, ns: no significance, as indicated.

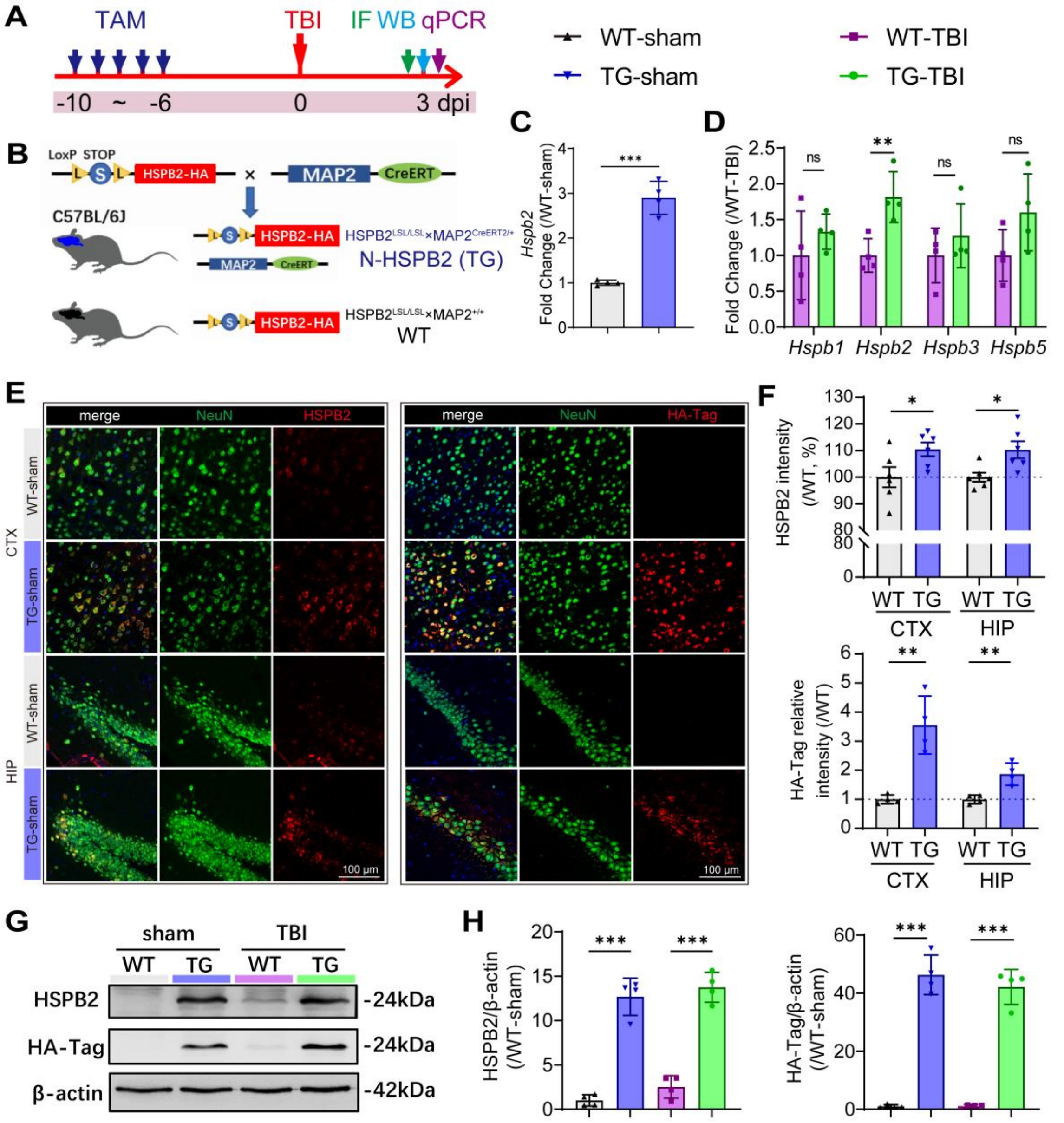

Figure S2 **Verification of HSPB2 overexpression transgenic mice. A** Schematic diagram of the experiment, TAM: tamoxifen; **B** Construction of Tamoxifen-induced neuron-specific HSPB2 overexpression mouse ; **C** Quantitative analysis of the mRNA levels of *Hspb2* at 3 days after injury, n=4, analyzed using one-way ANOVA and post hoc Bonferroni test; **D** Quantitative analysis of the mRNA levels of *Hspb1, 2, 3,* and *5* at 3 days post TBI, n=4, analyzed using unpaired Student's t-test **;E&F** Representative images and quantitative analysis of HSPB2 (E left & F up) and HA-Tag (E right & F down) expression in neurons in TG and WT (wild-type) mice, blue: DAPI, green: NeuN, red: HSPB2 or HA-Tag, scale bar = 100 μm, n=6, analyzed using unpaired Student's t-test; **G&H** Representative images and quantitative analysis of HSPB2 and HA-Tag relative expression at 3 days after injury, n=4, analyzed using one-way ANOVA and post hoc Bonferroni test. *: $p < 0.05$, **: $p < 0.01$, ***: $p < 0.001$, as indicated.

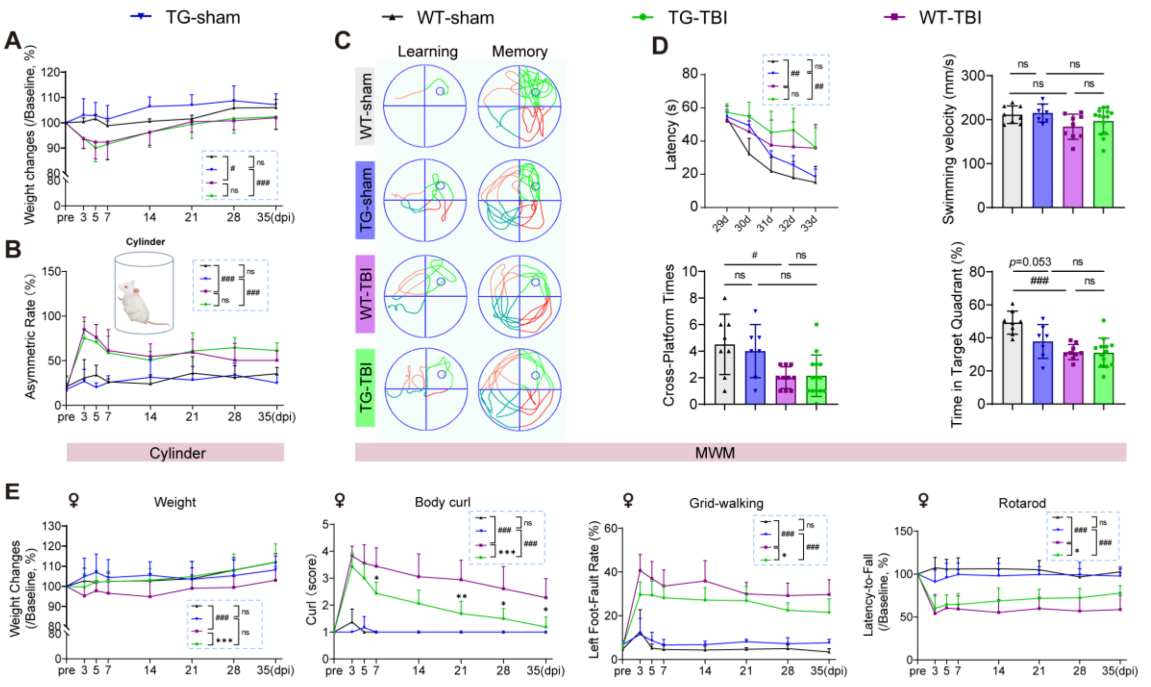

Figure S3 **Improved behavioral tests of male and female following TBI in TG mice. A** Weight changes following TBI, n=7, 7, 15, 17, respectively, analyzed using two-way ANOVA and post hoc Bonferroni test; **B** The forelimb asymmetry rate in the cylinder test, n=7, 7, 15, 17, respectively, analyzed using two-way ANOVA and post hoc Bonferroni test; **C** Representative routes of MWM during learning (d33) and memory (d34) stages; **D** Quantitative analysis of MWM, including learning stage latency from d29 to d33 (upper left), swimming velocity at d34 (upper right), platform crossing times at memory stage (lower left), and target quadrant traveling time (lower right), n=7, 7, 10, 15, respectively, analyzed using two- and one-way ANOVA and post hoc Bonferroni test; **E** Weight changes and behavioral tests for female mice, analyzed using two-way ANOVA and post hoc Bonferroni test. * TG *vs.* WT, # TBI *vs.* sham; */#: $p < 0.05$, **: $p < 0.01$, ***/###: $p < 0.001$, ns: no significance, as indicated.

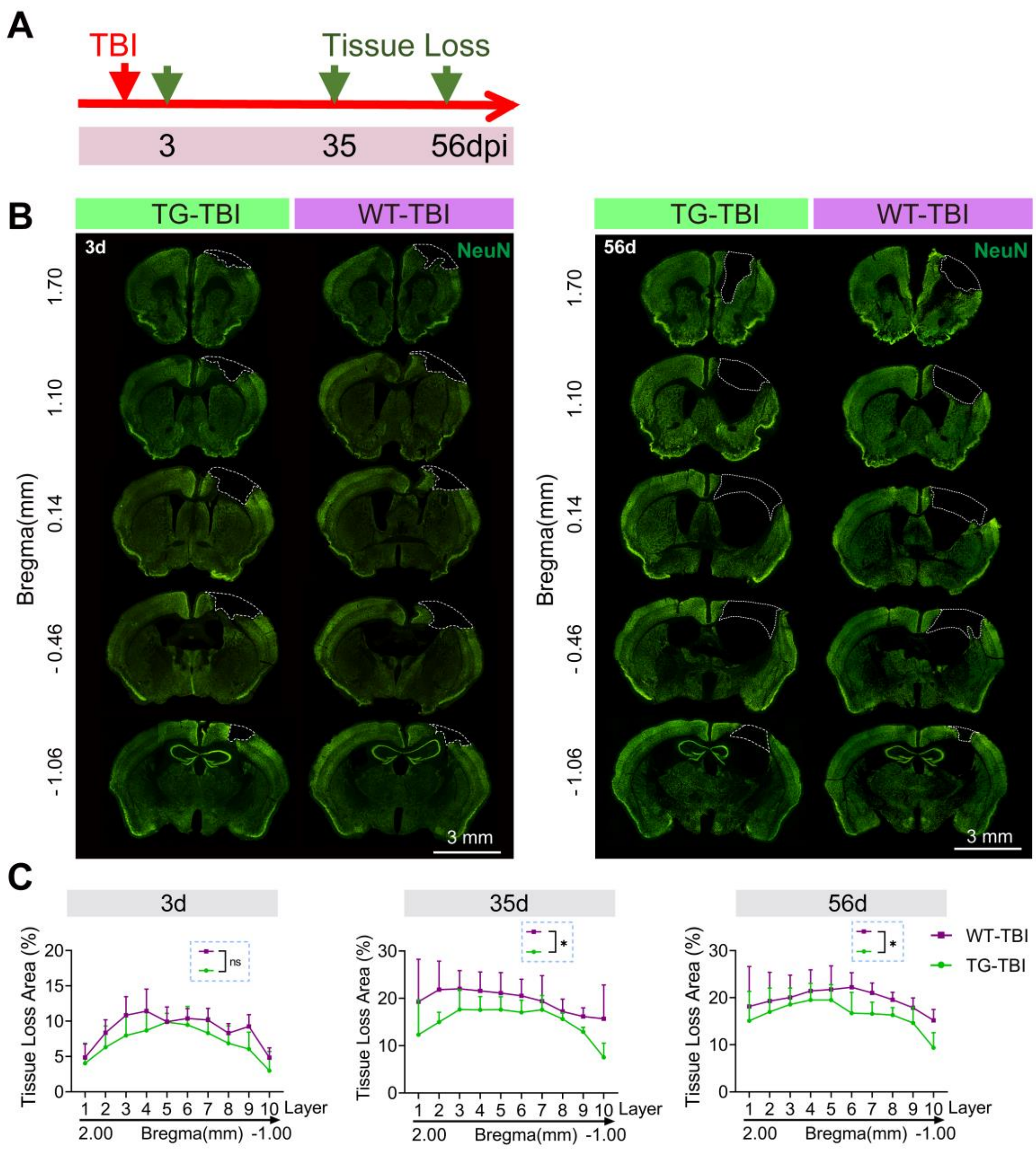

Figure S4 **Alleviated tissue loss following TBI in TG mice. A** Experimental design**; B** Illustration of tissue loss in injured layers at 3 and 56 days post-injury, with dotted boxes indicating loss area, scale bar = 3 mm; **C** Quantitative analysis of tissue loss area in injured layers, n=5-7, analyzed using two-way ANOVA and post hoc Bonferroni test. *: $p < 0.05$, ns: no significance, as indicated.

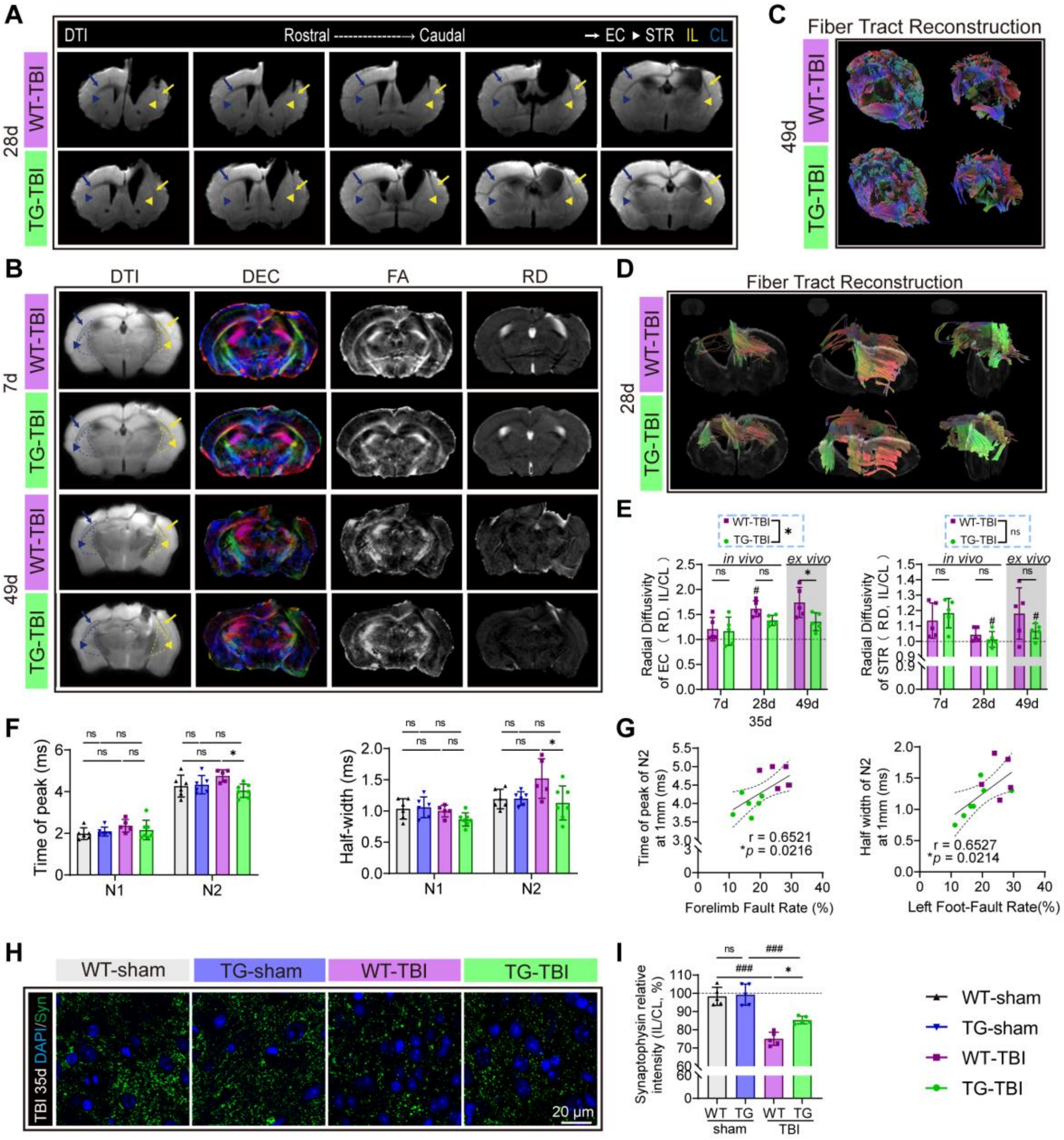

Figure S5 **Improved white matter integrity following TBI in TG mice verified by DTI and CAP. A** Illustration of DTI-MRI in different layers on day 28, triangle-arrowed dotted boxes indicate ROI STR, arrowed dotted boxes indicate ROI EC, yellow indicates the ipsilateral, blue indicates the contralateral; **B** Illustration of DTI-MRI and visualized parameters on day 7 (in vivo) and 49 (ex vivo); **C** Illustration of 3D reconstruction of fiber tracts on day 49 ex vivo, color indicating direction, from left to right: whole brain white matter fiber tracts, threshold-processed fiber tracts; **D** Illustration of 3D reconstruction of front view,

top view and side view of fiber tracts across the middle of CC on day 28 in vivo; **E** Quantitative analysis of relative RD of EC and STR and fiber tracts across middle of CC normalized to WT-TBI at 7 and 28 days (in vivo) and 49 days (ex vivo) following TBI, n=5, analyzed using two-way ANOVA and post hoc Bonferroni test, * TG-TBI *vs.* WT-TBI, $^{\#}$ indicates vs. 7d; **F** Quantitative analysis of time of peak and half-width of N1 and N2 of CAP, n=6-7, analyzed using one-way ANOVA and post hoc Bonferroni test. **G** Correlation analysis of forelimb fault rate in grid-walking test with time of peak/half-width of N2 at 35 days post-injury, r: Spearman correlation coefficient, n=6&7, analyzed using Spearman correlation test; **H** Illustration of synapses around injured area at 35 days after injury, blue: DAPI, green: synaptophysin, scale bar = 20 μm; **I** Quantitative analysis of synapse density in h, n=5, analyzed using one-way ANOVA and post hoc Bonferroni test. *: $p < 0.05$, $^{\#\#\#}$: $p < 0.001$, ns: no significance, as indicated.

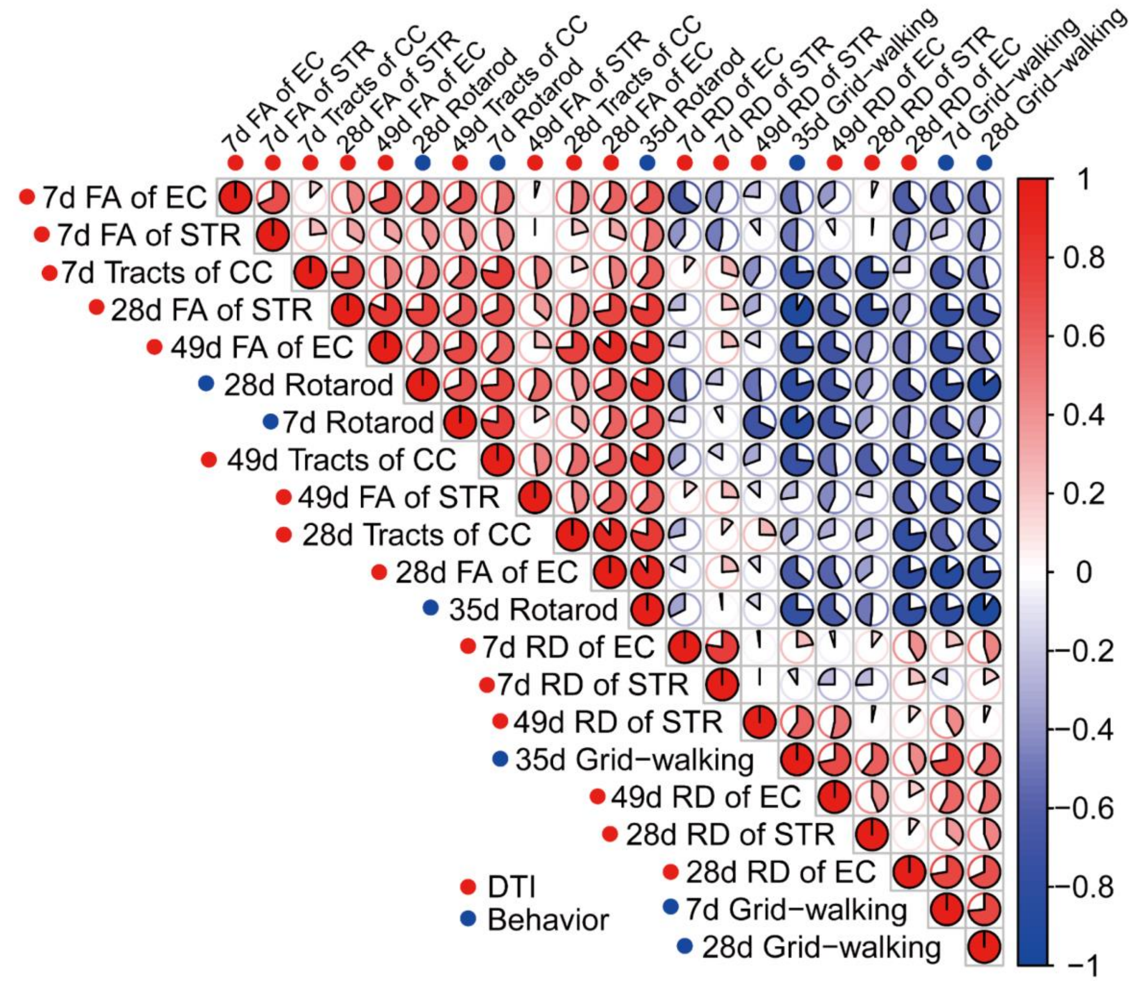

Figure S6 **Correlation matrix between DTI and behavior parameters.** Correlation matrix between DTI and behavioral indices at each time point, red: DTI indexes, blue: behavioral indexes, color intensity and fan size represent the Spearman r value, analyzed using Spearman correlation test.

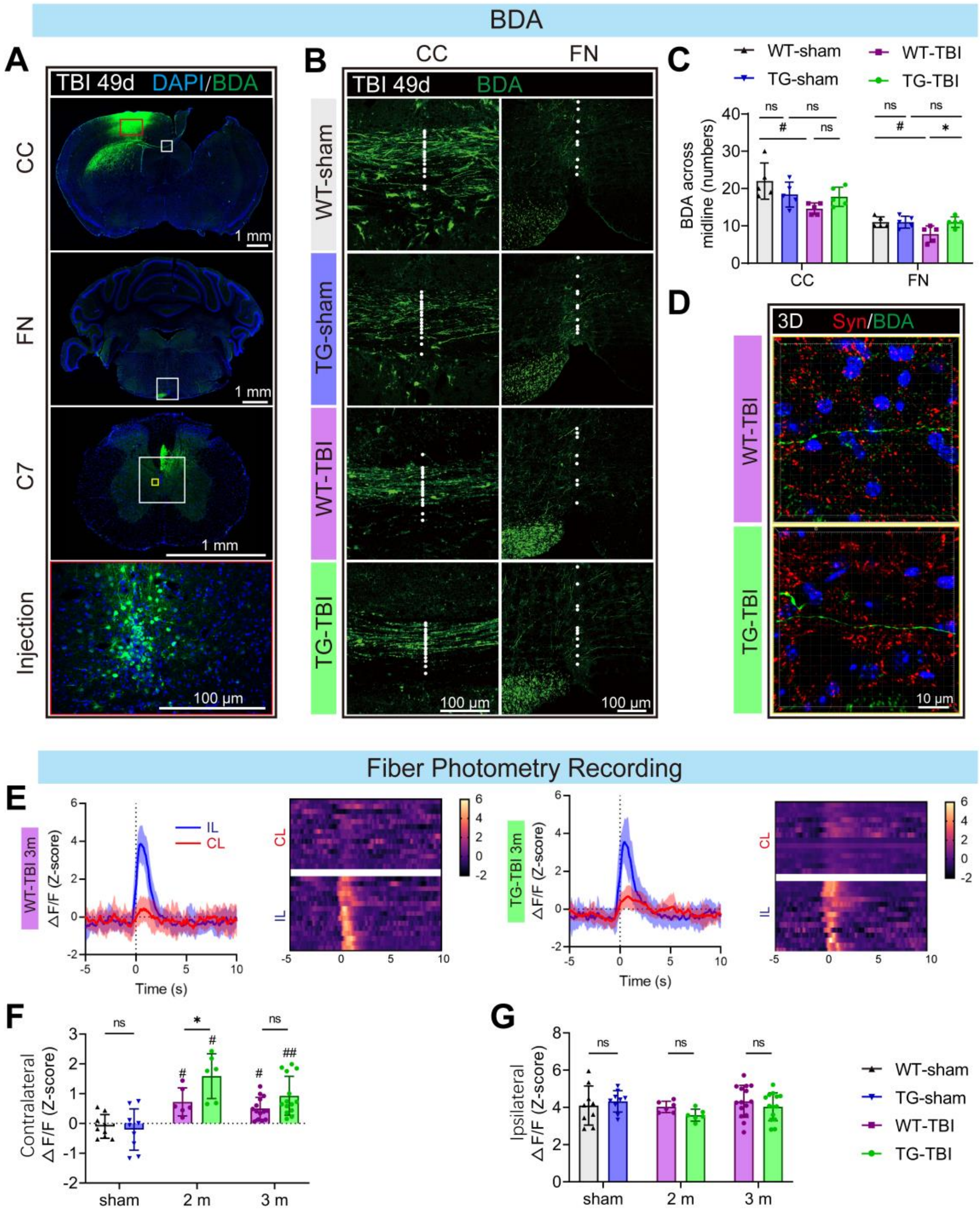

Figure S7 **Facilitated axon sprouting and cortical remapping following TBI in TG mice and verification of HPSB2 overexpression in vitro**. **A** Illustration of the BDA injection area and CST projection, CC: cerebrum corpus callosum layer, FN: medulla oblongata facial nucleus layer, C7: spine cord cervical segment 7, red box indicates injection area, white box indicates ROI for BDA across the midline, and yellow box indicates ROI for synapses on newly-formed BDA$^+$ axons, blue: DAPI, green: BDA, scale bar = 100 μm &

1 mm; **B** Illustration of BDA labeled axons crossing the midline of CC and FN layer at 49 days after injury, dot indicates the intersection of axon and midline, scale bar = 100 μm; **C** Quantitative analysis of BDA^+ axons crossing the midline of CC and FN layer at 49 days after injury, n = 5, analyzed using one-way ANOVA and post hoc Bonferroni test; **D** Illustration of 3D images of synaptophysin vesicles in newly-formed BDA^+ axons to be reconstructed in Figure 3F, blue: DAPI, red: synaptophysin, green: BDA, scale bar = 10 μm; **E** Line plots and heatmaps of the S1FL cortex fluorescent responses (z-scored △F/F) from -5 to 10 seconds after stimuli at each forepaw at 3 months, blue: ipsilateral, red: contralateral; **F&G** Quantitative analysis of contralateral (f) and ipsilateral (g) forepaw △F/F from 2 to 3 months after injury, n = 3 × 3 trails (sham), 2 × 3 trails (2 months), 5 × 3 trails (3 months) , analyzed using two-way ANOVA and post hoc Bonferroni test. * TG *vs.* WT, $^{\#}$ TBI *vs.* sham, */$^{\#}$: $p < 0.05$, **/$^{\#\#}$: $p < 0.01$, ***/$^{\#\#\#}$: $p < 0.001$, ns: no significance.

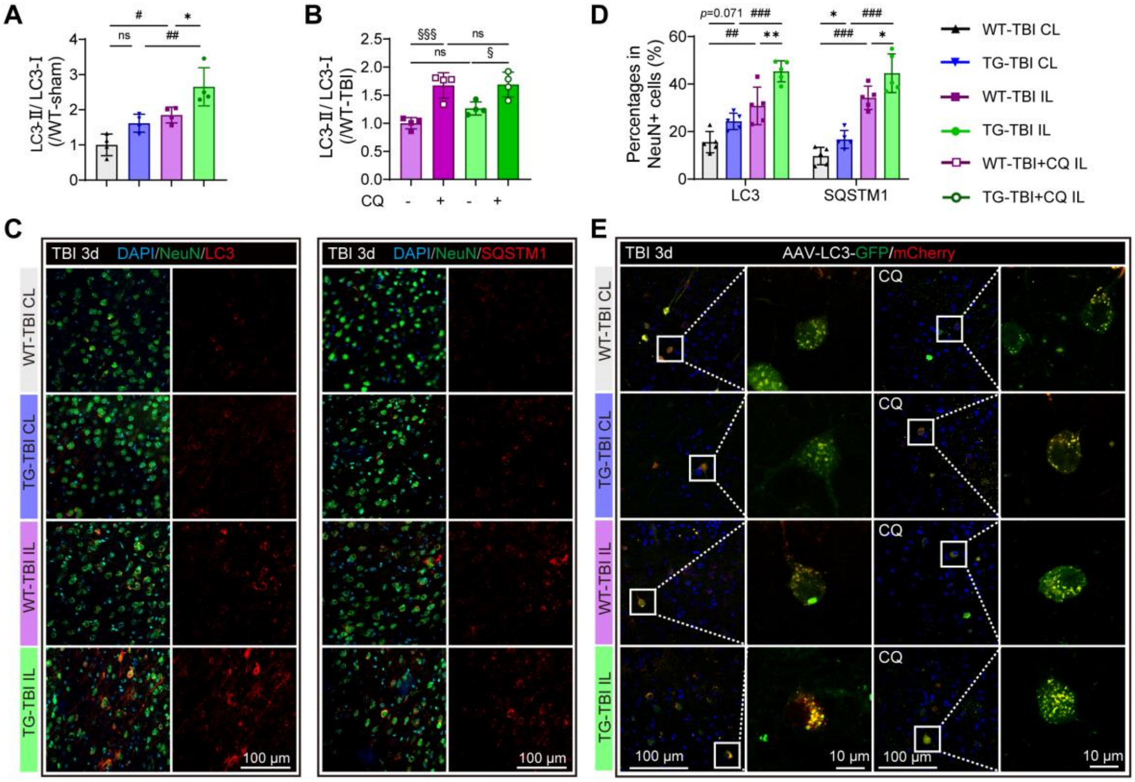

Figure S8 **Enhanced neuro-autophagy following TBI in TG mice. A&B** Quantitative analysis of LC3-Ⅱ/Ⅰ in Figure 4E, 4G, n=4, analyzed using one-way ANOVA and post hoc Bonferroni test; **C** Illustration of LC3 (left) and SQSTM1 (right) levels in neurons in CTX 3 days following TBI, blue: DAPI, green: NeuN, red: LC3 (left) / SQSTM1 (right), scale bar =100 μm; **D** Quantitative analysis of LC3 and SQSTM1 positive neuron's percentages in total neurons in CTX 3 days following TBI n=5, analyzed using one-way ANOVA and post hoc Bonferroni test; **E** Illustration of autophagosome and autolysosome vesicles in neurons in CTX of ipsilateral and contralateral sides with/without CQ administration at 3 days following TBI, blue: DAPI, red: autolysosome, yellow: autophagosome, boxes indicate enlarged area. dpi: day post injury. *: $p < 0.05$, **/$^{\#\#}$: $p < 0.01$, $^{\#\#\#}$: $p < 0.001$, ns: no significance, as indicated.

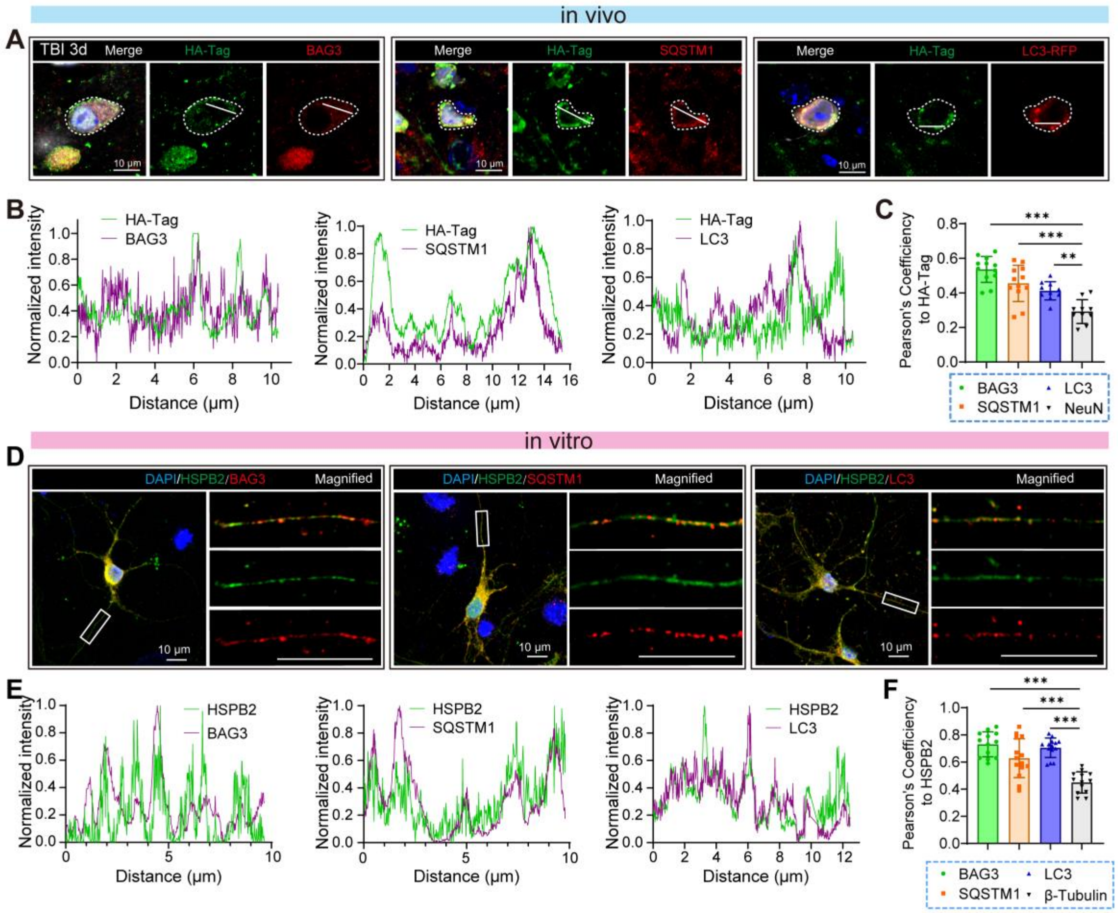

Figure S9 **HSPB2's colocalization in vivo and in vitro. A** Representative images of cell body colocalization of HA-Tag with BAG3, SQSTM1, and LC3 at 3 days following TBI, blue: DAPI, green: HA-Tag, red: BAG3, SQSTM1, and LC3, grey: NeuN, line indicates region of plot profile, scale bar = 10 μm; **B** plot profile demonstrating cytosolic HA-Tag and BAG3/SQSTM1/LC3 colocalizations; **C** Quantitative analysis of Pearson's correlation coefficient of HA-Tag with BAG3, SQSTM1, LC3 and NeuN, n=10-15, analyzed using one-way ANOVA and post hoc Bonferroni test; **D** Illustration of axonal colocalization of HSPB2 with BAG3, SQSTM1, and LC3 in primary neuron culture after OGD, blue: DAPI, green: HSPB2, red: BAG3, SQSTM1, or LC3, box indicates magnified area, scale bar = 10 μm; **E** plot profile demonstrating axonal HSPB2 and BAG3/SQSTM1/LC3 colocalizations; **F** Quantitative analysis of Pearson's correlation coefficient of HSPB2 with BAG3, SQSTM1, LC3 and β-Tubulin, n=10-15, analyzed using one-way ANOVA and post hoc Bonferroni test. **: $p < 0.01$, ***: $p < 0.001$, ns: no significance, as indicated.

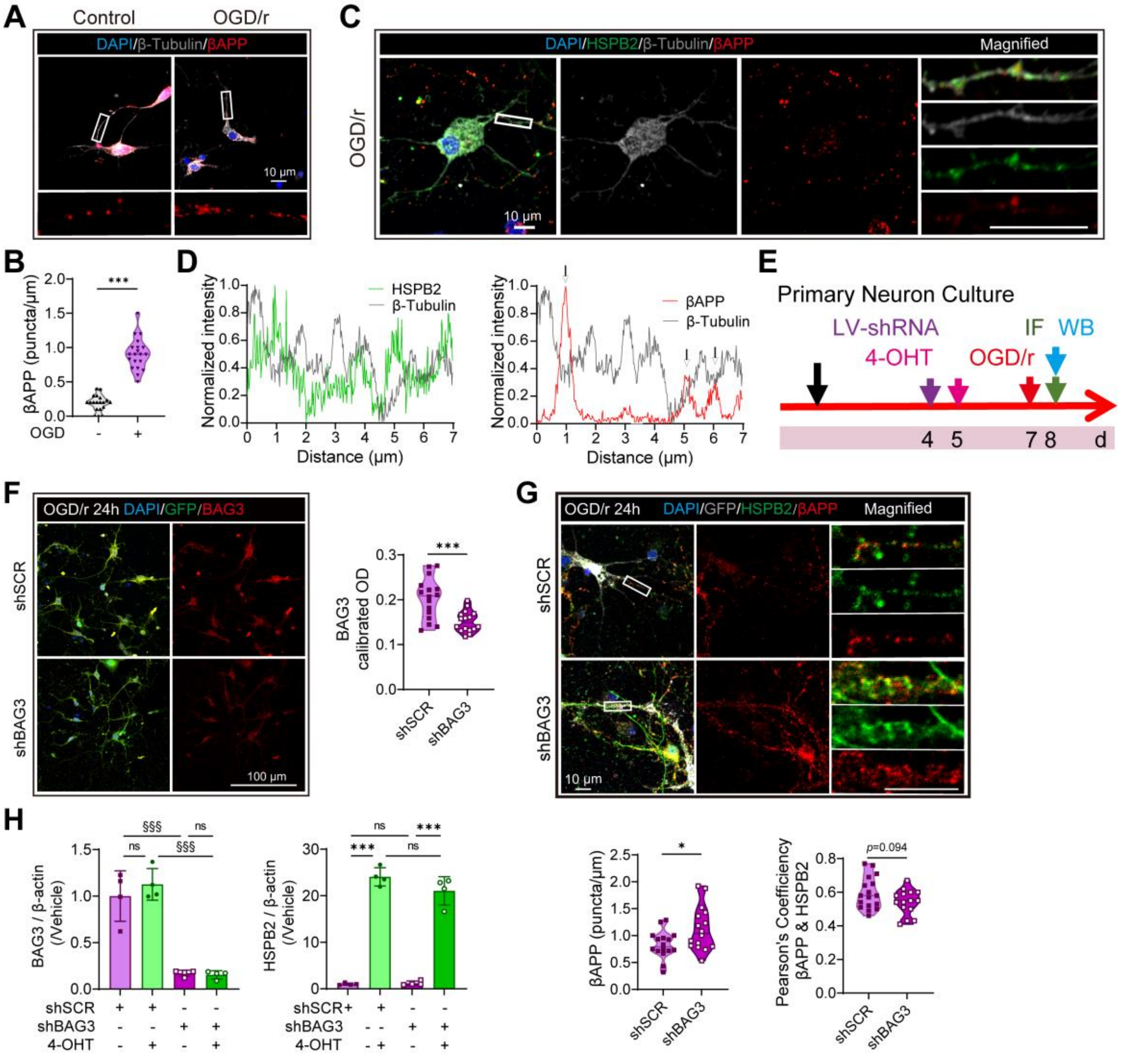

Figure S10 **βAPP's presence in vitro and BAG3 silencing. A&B** Illustration and quantitative analysis of axonal βAPP in primary neuron culture with or without OGD blue: DAPI, red: βAPP, grey: β-Tubulin, n = 20, analyzed using unpaired Student's t-test; **C** Illustration of axonal colocalization of βAPP with HSPB2 and β-Tubulin in primary neuron culture in OGD, blue: DAPI, green: HSPB2, red: βAPP, grey: β-Tubulin, box indicates magnified area, scale bar = 10 μm; **D** Plot profile demonstrating axonal β-Tubulin and HSPB2/βAPP colocalizations; **E** Experimental design for HSPB2 overexpression and BAG3 silence experiments in vitro; **F** Illustration and quantitative analysis of BAG3 in primary neuron culture with shSCR or shBAG3, blue: DAPI, red: BAG3, green: GFP, n = 16, analyzed using unpaired Student's t-test; **G** Illustration and quantitative analysis of of axonal βAPP and its colocalization with HSPB2 in primary neuron culture after OGD with or without BAG3 silencing, box indicates magnified area, scale bar = 10 μm**,** n=15-17, analyzed using unpaired Student's t-test; **G** Quantitative analysis of BAG3 and HSPB2 24h post OGD with or without BAG3 silencing or HSPB2 overexpression, n=4, analyzed using one-way ANOVA and post hoc Bonferroni test. 4-OHT: 4-Hydroxytamoxifen. § shBAG3 *vs.* shSCR. */§: $p < 0.05$, **/§§: $p < 0.01$, ***/§§§: $p < 0.001$, ns: no significance, as indicated.

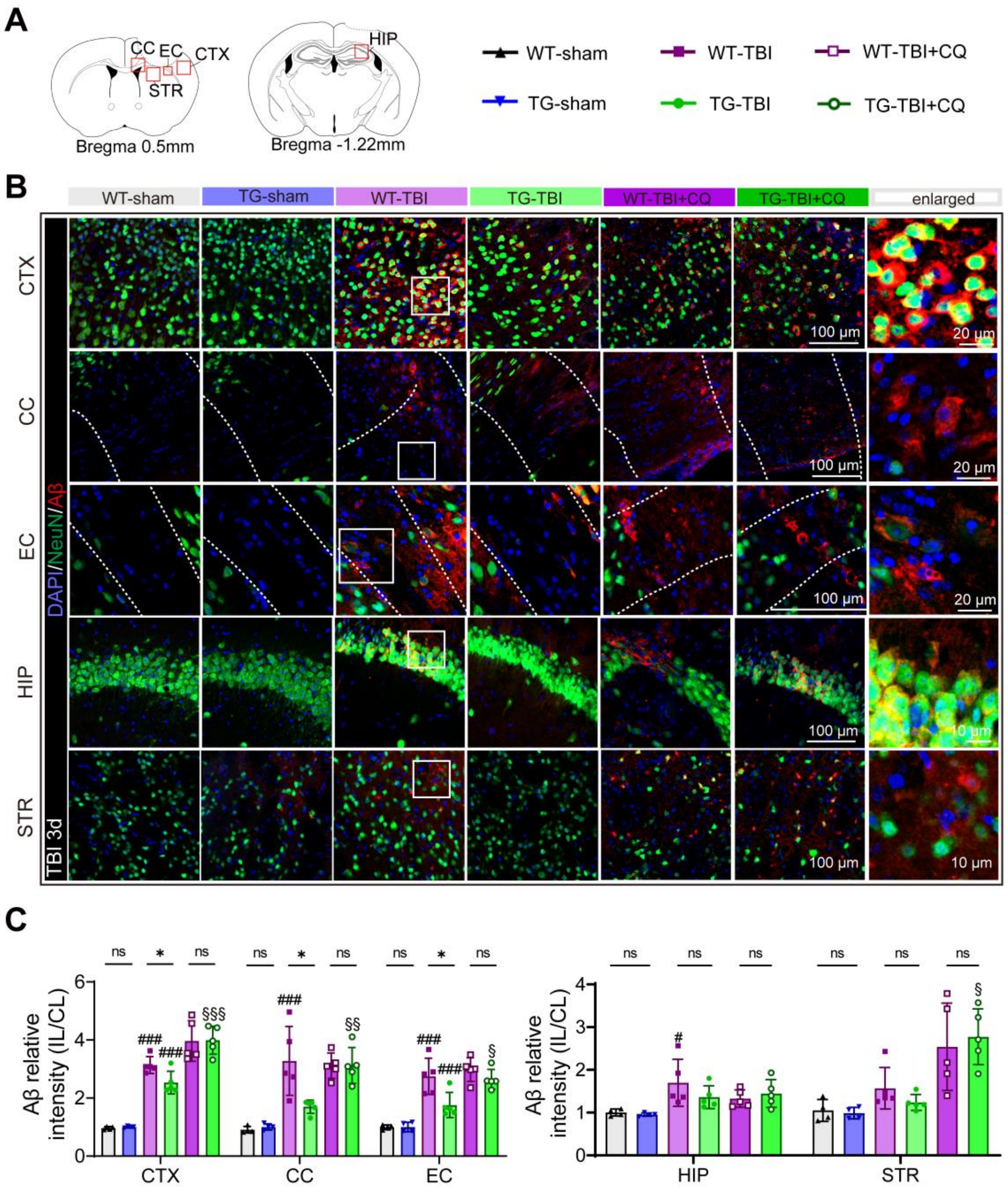

Figure S11 **Reduced Aβ deposition following TBI in TG mice through autophagy. A** ROI of CC, EC, CTX, STR, and HIP; **B** Illustration of Aβ aggregation in cortex, corpus callosum, external capsule, striatum and hippocampus with or without CQ at 3 days post-injury, dotted boxes indicate ROI, boxes indicate enlarged area, blue: DAPI, green: NeuN, red: Aβ; **C** Quantitative analysis of Aβ aggregation, n=4-5, analyzed using one-way ANOVA and post hoc Bonferroni test. ns: no significance.

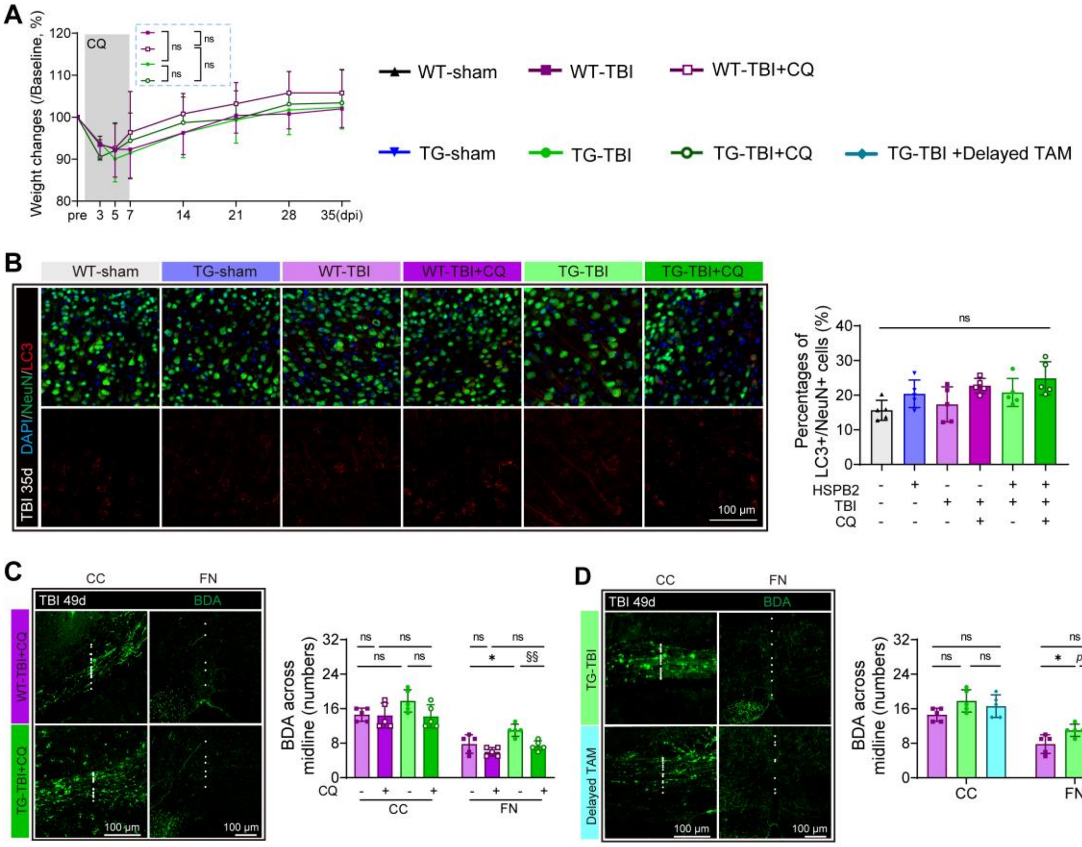

Figure S12 **HSPB2’s pro-recovery effects were reversed by acute-stage CQ administration or delayed induction. A** Quantitative analysis of weight change from 3 to 35 days post-injury with CQ, n=15, 10, 17, 10, respectively, analyzed using two-way ANOVA and post hoc Bonferroni test; **B** Illustration and quantitative analysis of LC3 levels in neurons in CTX after 35 days following TBI, blue: DAPI, green: NeuN, red: LC3, scale bar =100 μm, n=5, analyzed using one-way ANOVA and post hoc Bonferroni test;**C&D** Illustration and quantification of BDA fiber crossing midline in CC and FN at 49 days post-injury with CQ (c) or delayed TAM (d), scale bar = 100 μm, n=5, analyzed using one-way ANOVA and post hoc Bonferroni test. * indicates TG vs. WT, § indicates CQ vs. without CQ or delayed vs. normal TAM. *: $p < 0.05$, ns: no significance, as indicated.

Supplemental Tables

Table S1 Correlation matrix of DTI and behaviors

Please see a file for the other supporting materials: Table.S1.xlsx

Table S2 Docking prediction of βAPP, HSPB2 and BAG3

Protein A	Protein B	interface area, Å2	∆iG, kcal/mol	∆iG P-value
APP	HSPB2	2596.6	-32.4	0.345
BAG3	HSPB2	2113.4	-18.0	0.550

Protein structures were obtained from Alphafold, HSPB2: AF-Q16082-F1, APP: AF-P05067-F1,BAG3: AF-O95817-F1; docking was predicted by GRAMM-X, method: rigid docking; interactions were quantified by PDBePISA

Table S3 Correlation matrix of autophagy vesicles, foot-fault rates and βAPP accumulation

Please see a file for the other supporting materials: Table.S3.xlsx

Table S4 Statistical analysis

Please see a file for the other supporting materials: Table.S4.docx

Supplemental Movies

Movie S1. HSPB2 increased the fiber tracts across the middle of CC at day 49. The representative 3D structures of the projection of the fiber tracts across the middle of CC of WT-TBI and TG-TBI mice at 49 days post injury, using the software DSI-studio.

Please see a file for the other supporting materials: Movie. S1.mp4

Movie S2 Calcium fiber photometry recording. The process of calcium fiber photometry recording when stimulating each forepaw and their calcium responses.

Please see a file for the other supporting materials: Movie. S2.mp4

Supplemental References

1. G. Wang *et al.*, Scriptaid, a novel histone deacetylase inhibitor, protects against traumatic brain injury via modulation of PTEN and AKT pathway : scriptaid protects against TBI via AKT. *Neurotherapeutics* **10**, 124-142 (2013).

2. Y. Zhao *et al.*, Microglia-specific deletion of histone deacetylase 3 promotes inflammation resolution, white matter integrity, and functional recovery in a mouse model of traumatic brain injury. *J Neuroinflammation* **19**, 201 (2022).

3. A. M. A. Anthony Jalin, R. Jin, M. Wang, G. Li, EPPS treatment attenuates traumatic brain injury in mice by reducing A^2 burden and ameliorating neuronal autophagic flux. *Experimental neurology* **314**, 20-33 (2019).

4. P. M. Washington *et al.*, The effect of injury severity on behavior: a phenotypic study of cognitive and emotional deficits after mild, moderate, and severe controlled cortical impact injury in mice. *J Neurotrauma* **29**, 2283-2296 (2012).

5. P. Wei *et al.*, Cordycepin confers long-term neuroprotection via inhibiting neutrophil infiltration and neuroinflammation after traumatic brain injury. *J Neuroinflammation* **18**, 137 (2021).

6. X. Liu *et al.*, Interleukin-4 Is Essential for Microglia/Macrophage M2 Polarization and Long-Term Recovery After Cerebral Ischemia. *Stroke* **47**, 498-504 (2016).

7. M.-Y. Xu, Y.-F. Wang, P.-J. Wei, Y.-Q. Gao, W.-T. Zhang, Hypoxic preconditioning improves long-term functional outcomes after neonatal hypoxia-ischemic injury by restoring white matter integrity and brain development. *Cns Neurosci Ther* **25**, 734-747 (2019).

HSPB2 expression after TBI – CTX

Full unedited gel for **Figure.1B**

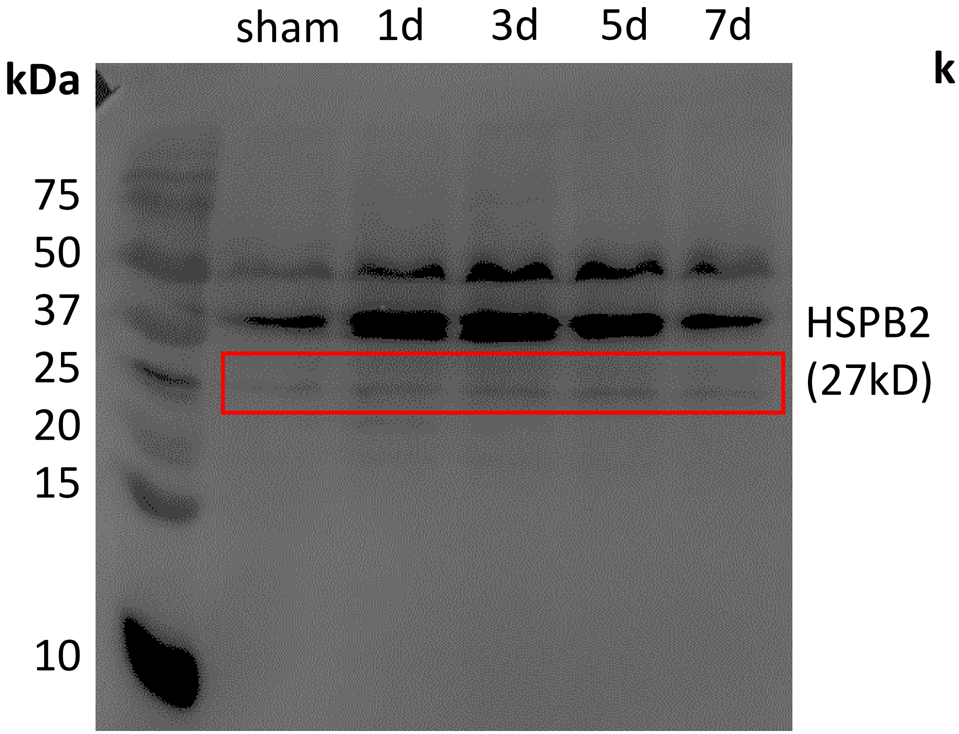

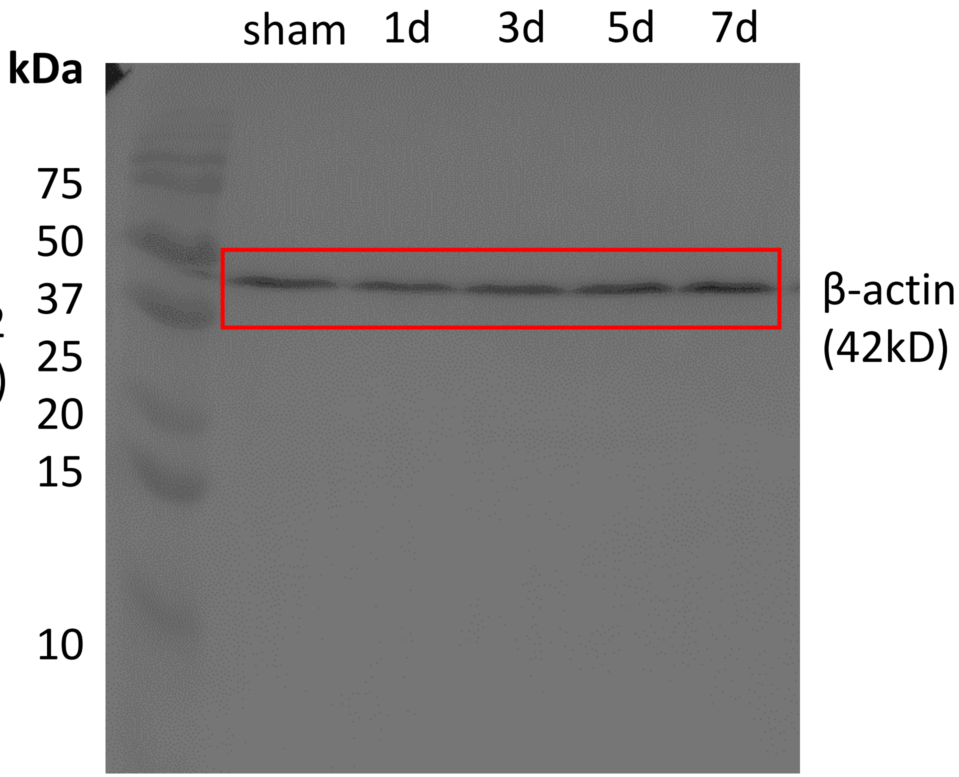

On 12% SDS-PAGE

HSPB2 expression after TBI– HIP Full unedited gel for **Figure.1B**

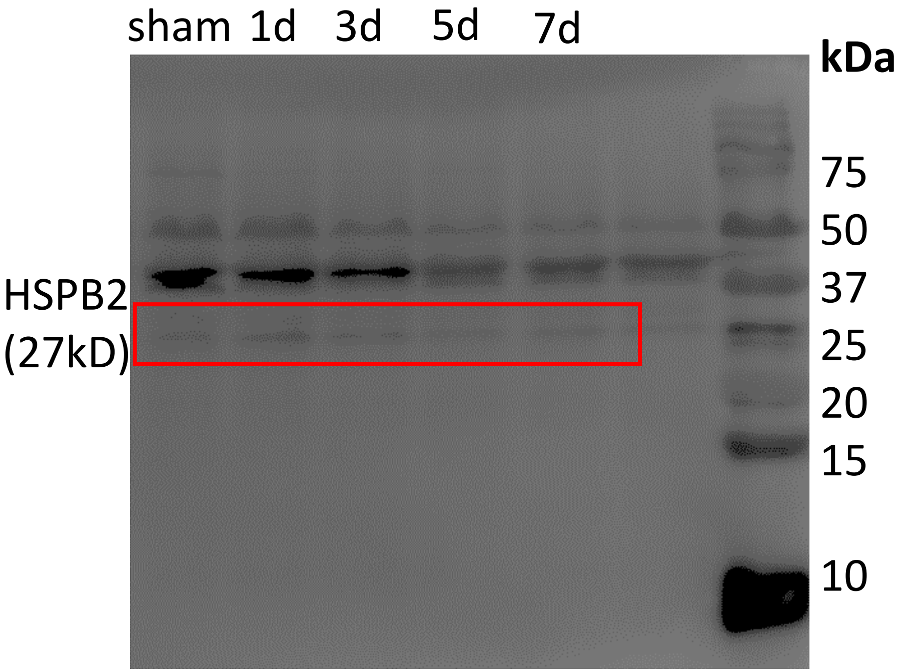

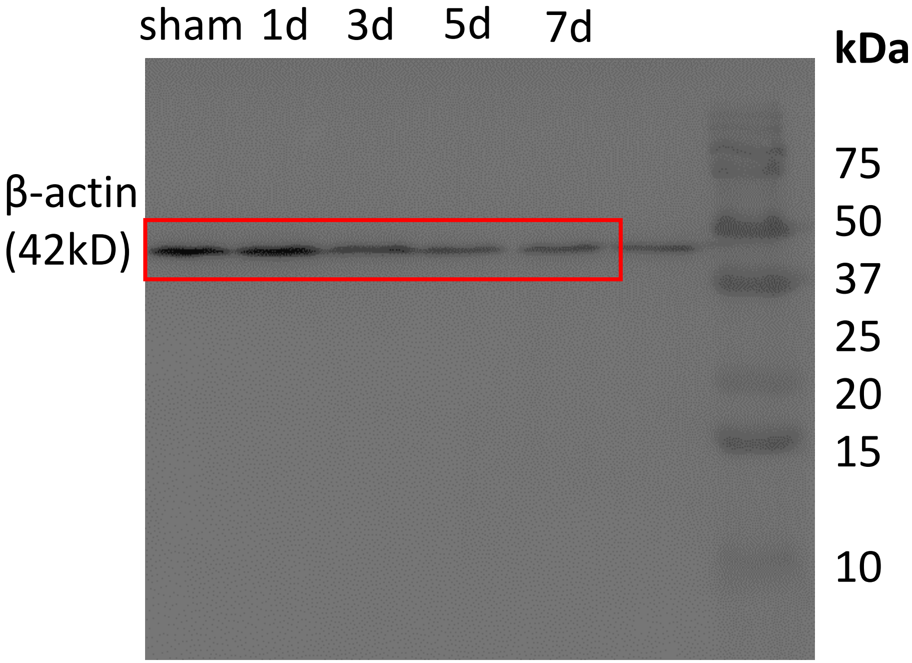

On 12% SDS-PAGE

LC3 after TBI 3d

Full unedited gel for **Figure.7D**

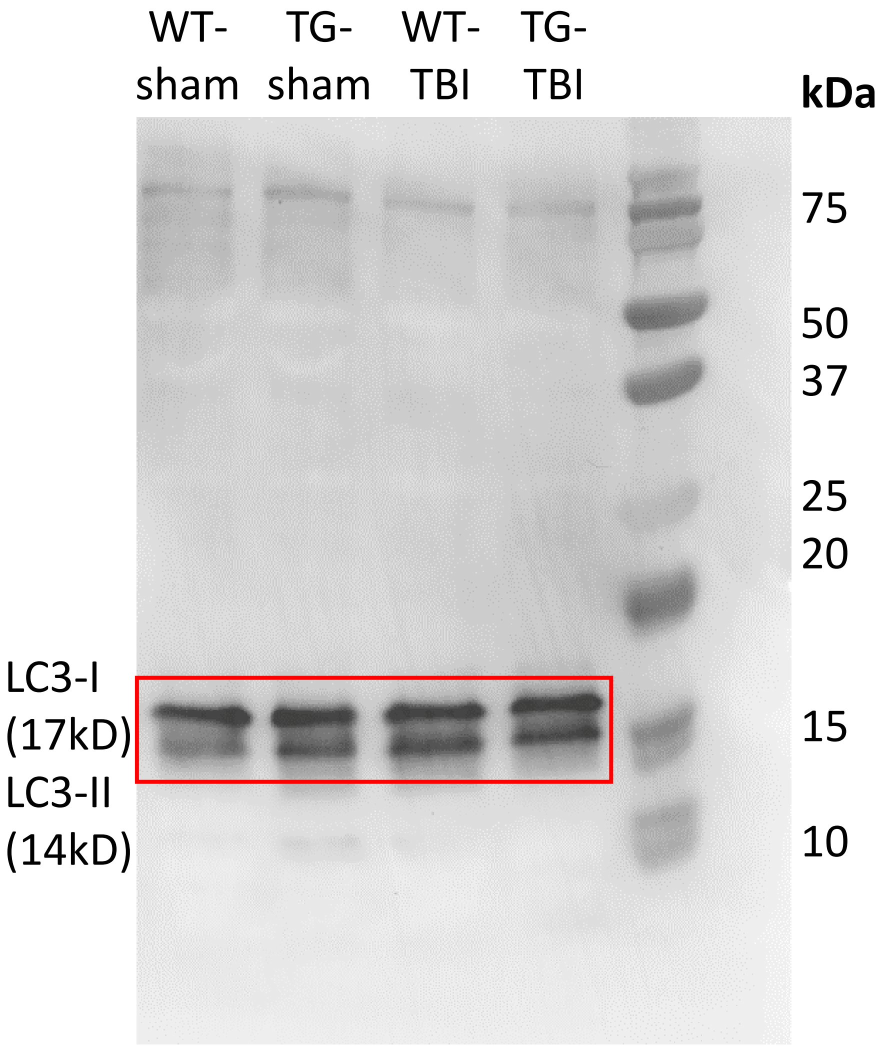

On 15% SDS-PAGE

SQSTM1 after TBI 3d Full unedited gel for **Figure.7D**

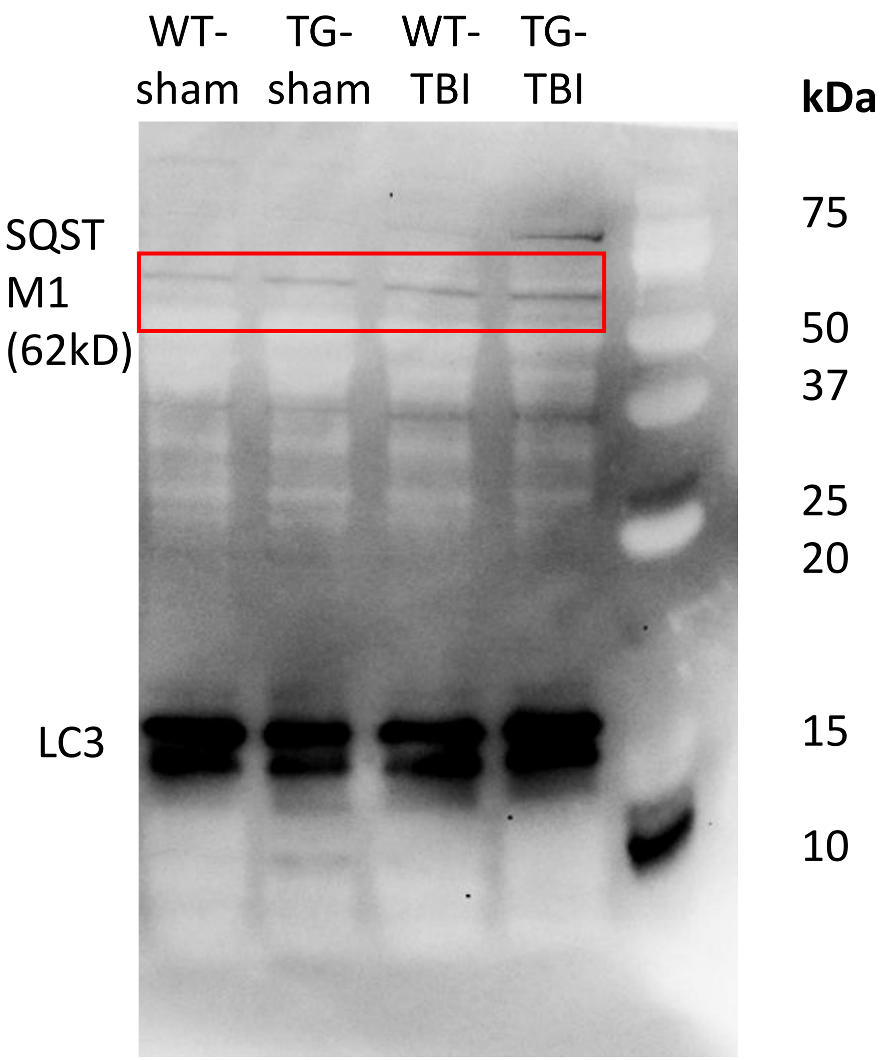

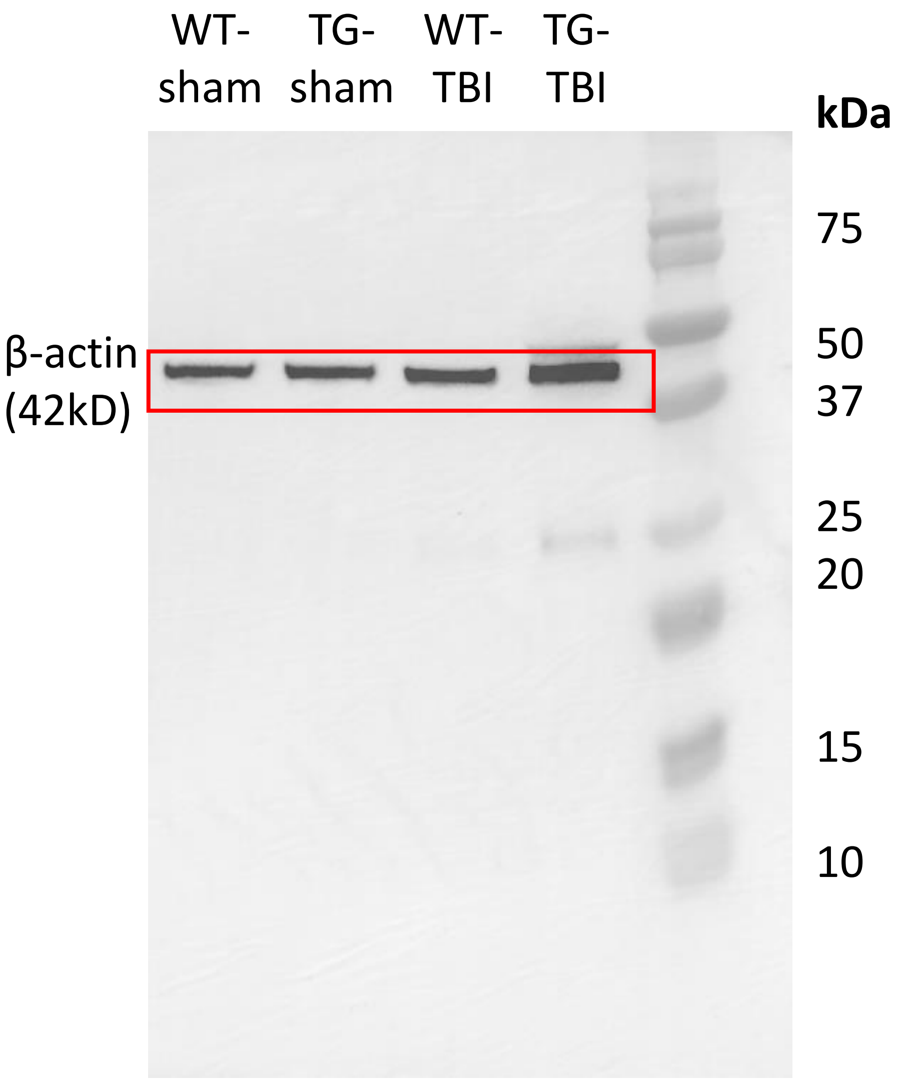

On 15% SDS-PAGE stripped after “**LC3 after TBI**”

SQSTM1 after TBI+CQ 3d

Full unedited gel for **Figure. 7E**

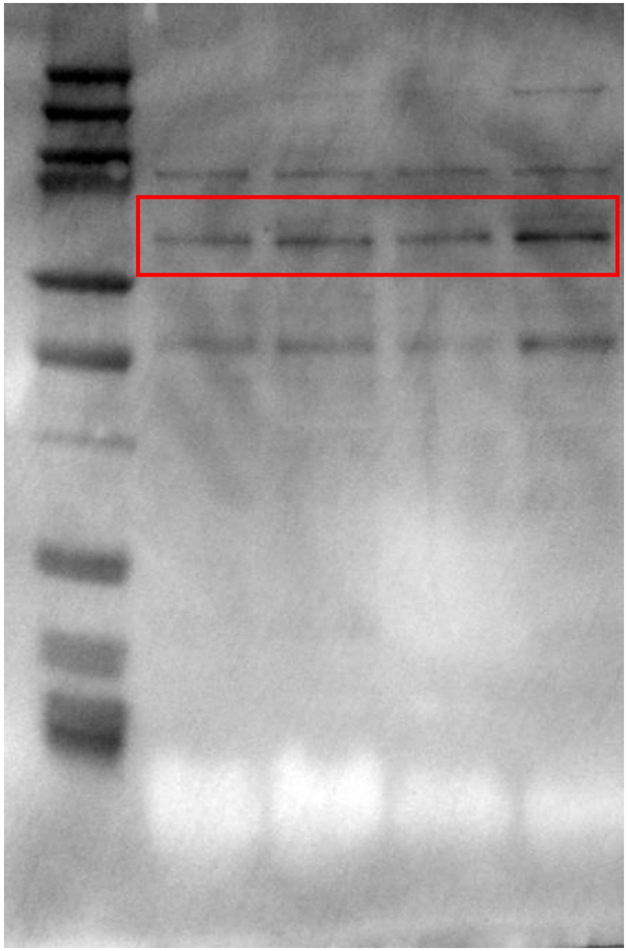

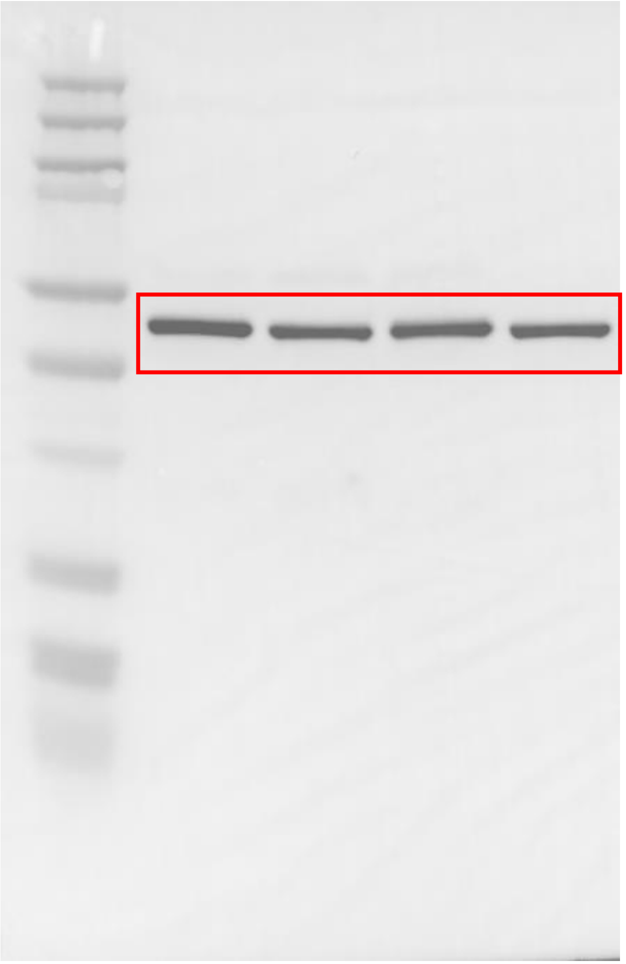

On 15% SDS-PAGE

LC3 after TBI+CQ 3d

Full unedited gel for **Figure.7E**

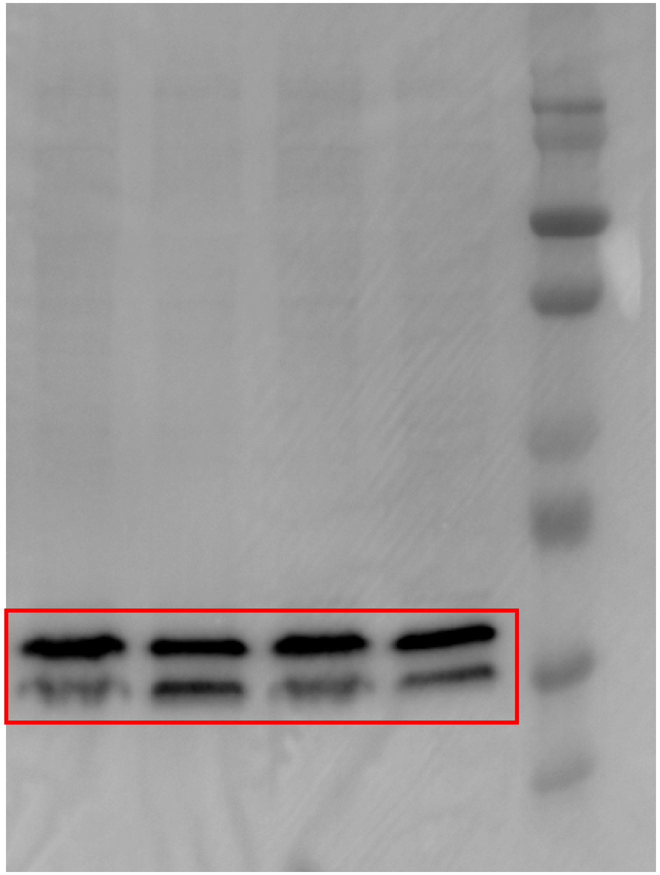

On 15% SDS-PAGE

p-mTOR/mTOR after TBI 3d Full unedited gel for **Figure.9B**

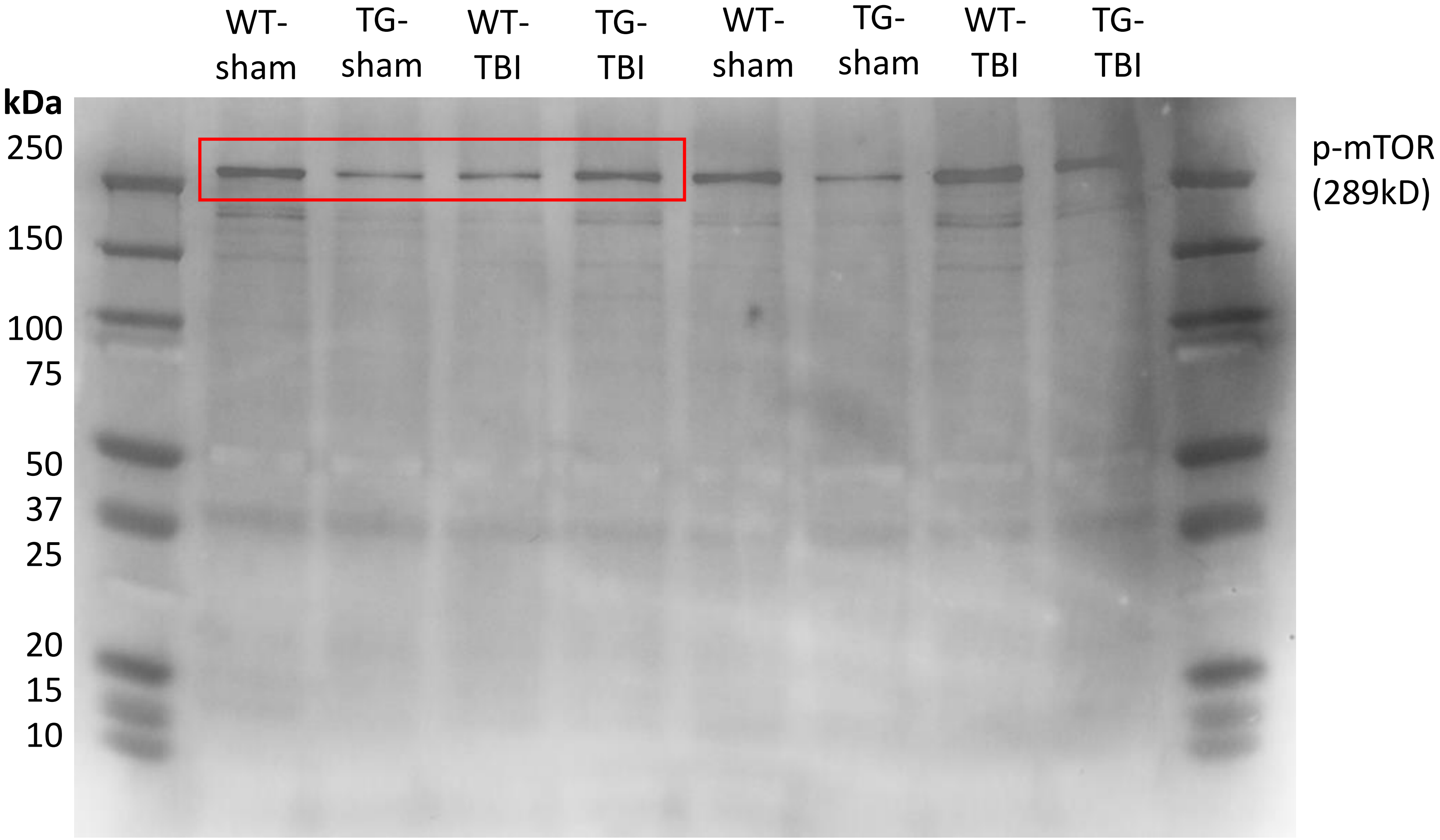

On 4-20% SDS-PAGE

p-mTOR/mTOR after TBI 3d

Full unedited gel for **Figure.9B**

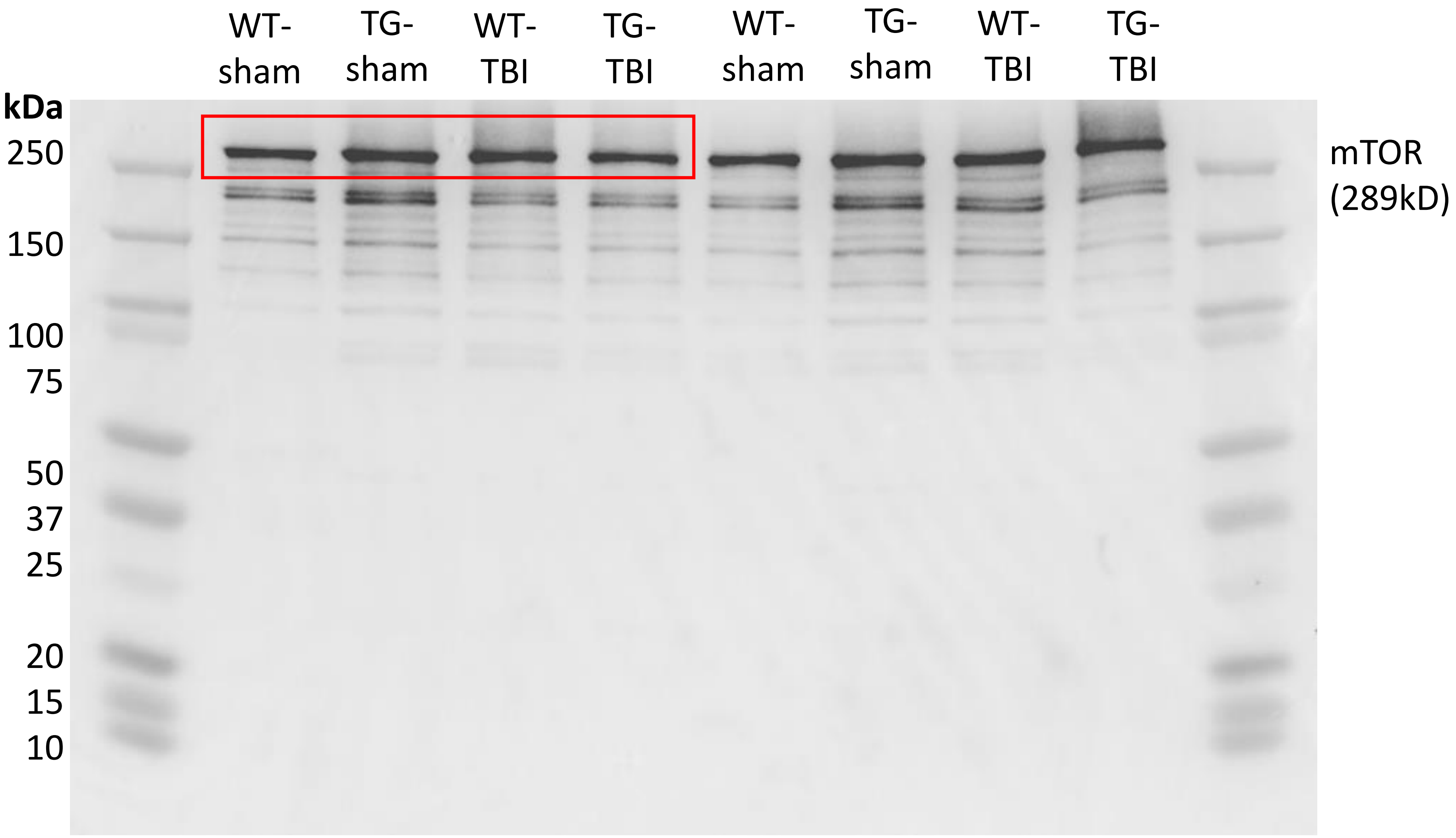

On 4-20% SDS-PAGE

stripped after “**p-mTOR after TBI 3d**”

p-mTOR/mTOR after TBI 3d

Full unedited gel for **Figure.9B**

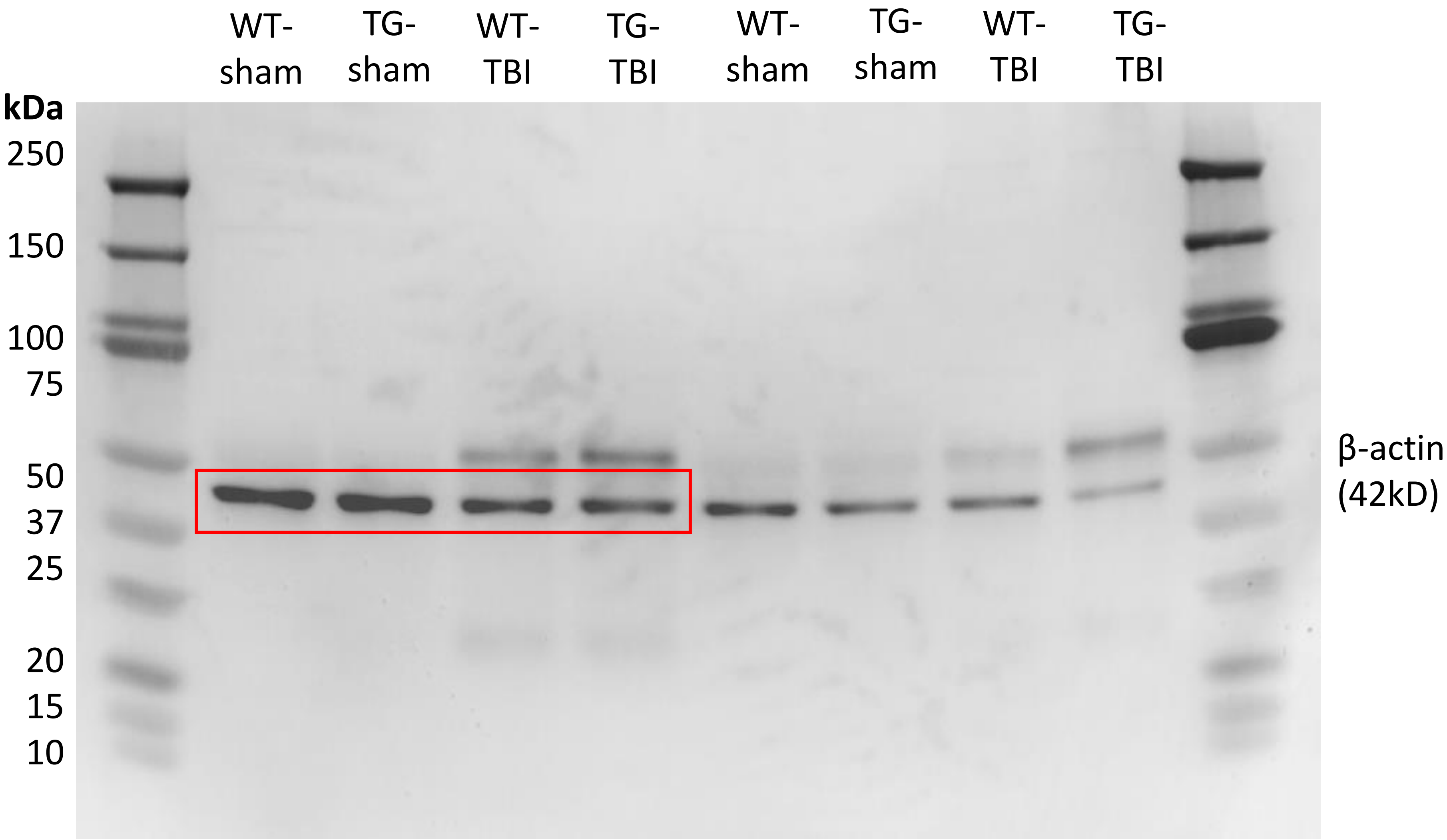

On 4-20% SDS-PAGE

stripped after "**p-mTOR after TBI 3d**"

p-Akt/Akt after TBI 3d Full unedited gel for **Figure.9B**

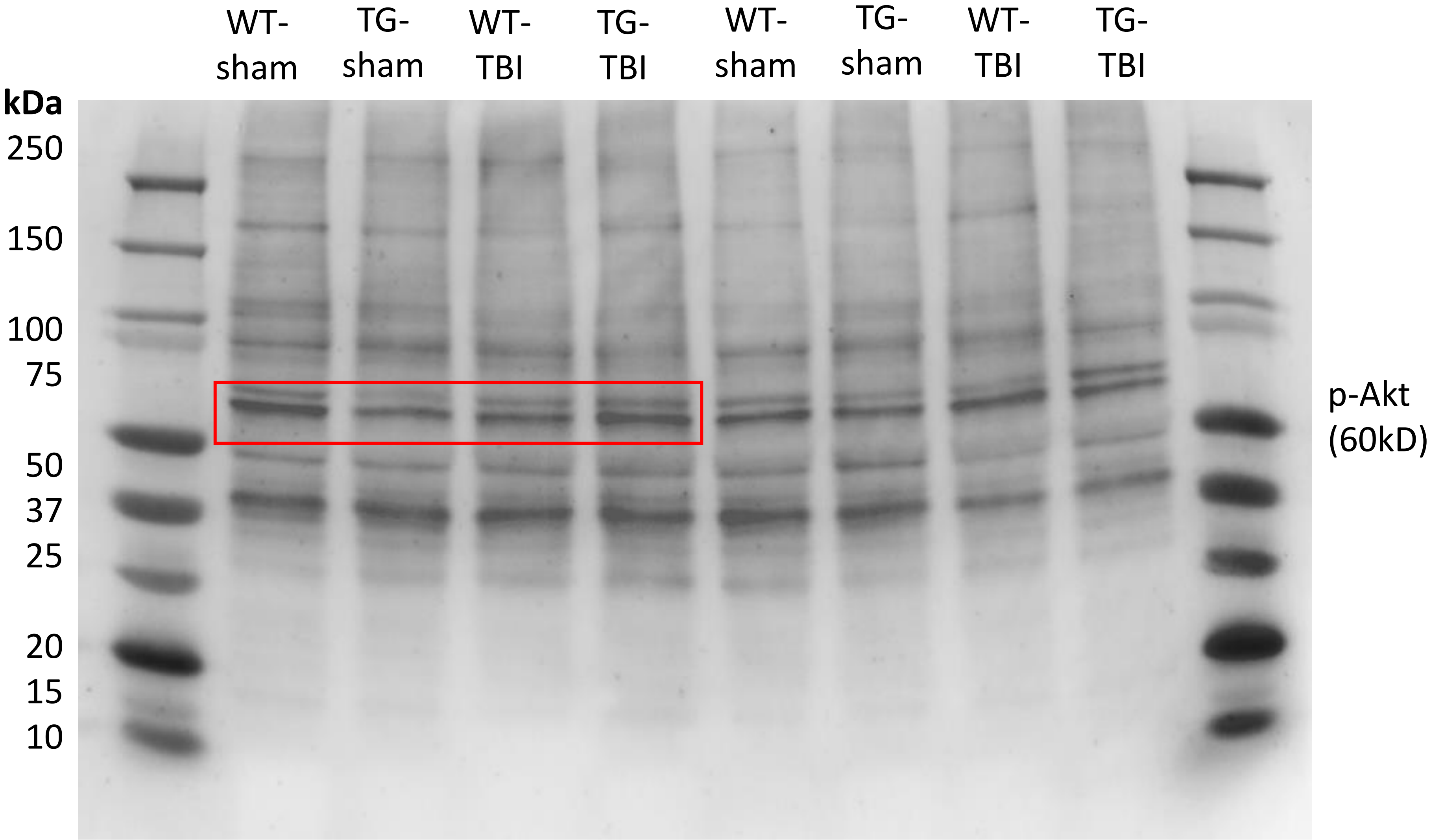

On 4-20% SDS-PAGE

p-Akt/Akt after TBI 3d

Full unedited gel for **Figure.9B**

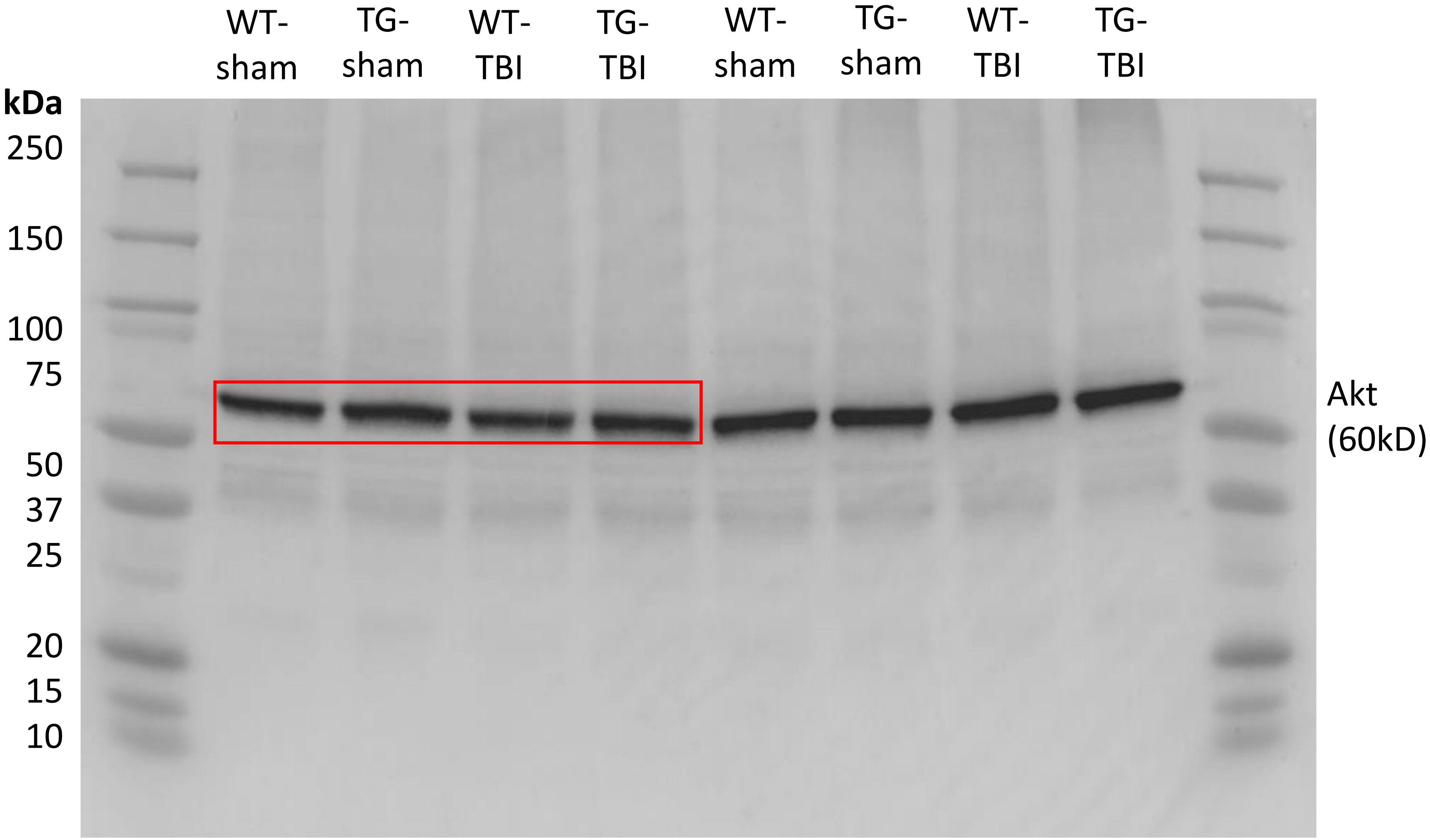

On 4-20% SDS-PAGE

stripped after "**p-Akt/Akt after TBI 3d**"

p-Akt/Akt after TBI 3d

Full unedited gel for **Figure.9B**

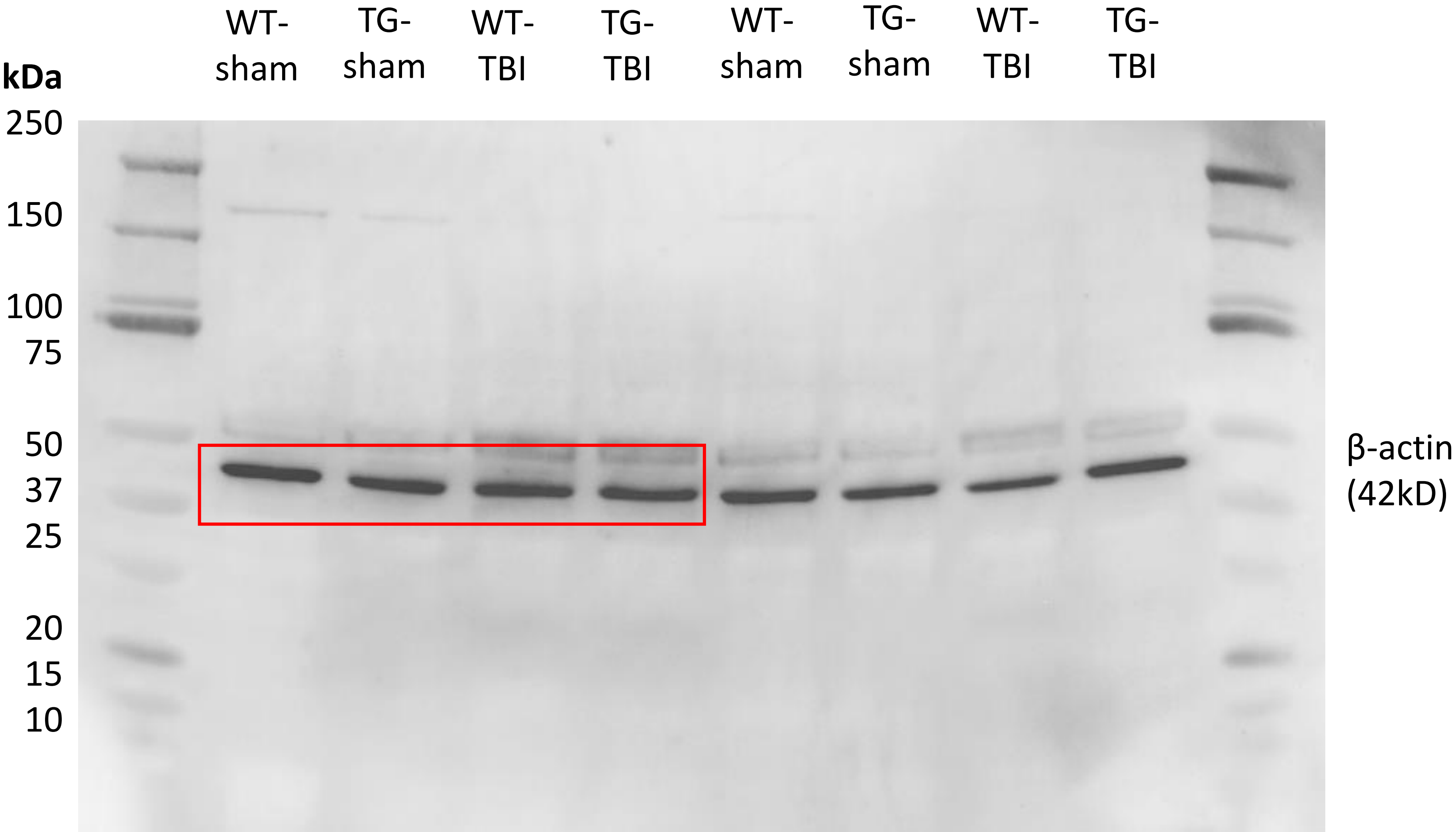

On 4-20% SDS-PAGE

stripped after “**p-Akt/Akt after TBI 3d**”

GAP-43 after TBI 3d

Full unedited gel for **Figure.9B**

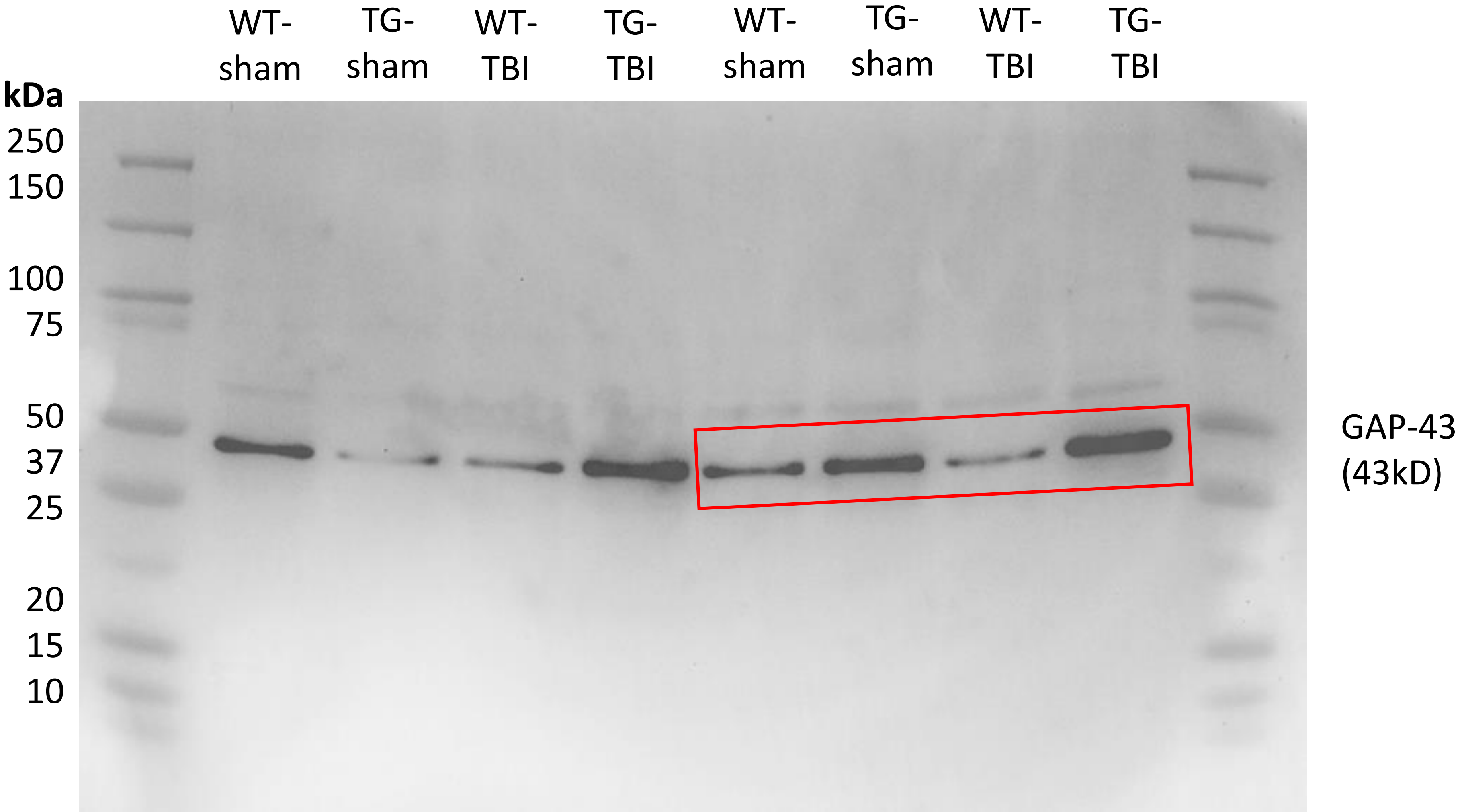

On 4-20% SDS-PAGE

stripped after “**p-Akt/Akt after TBI 3d**”

p-mTOR/mTOR after TBI +CQ 3d

Full unedited gel for **Figure.9D**

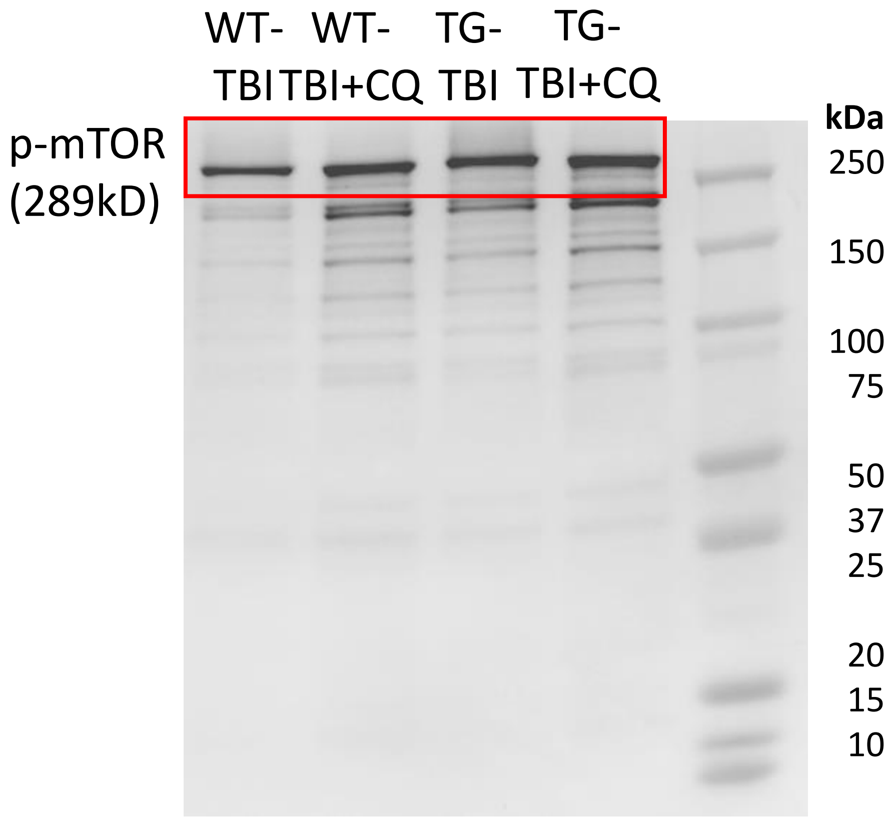

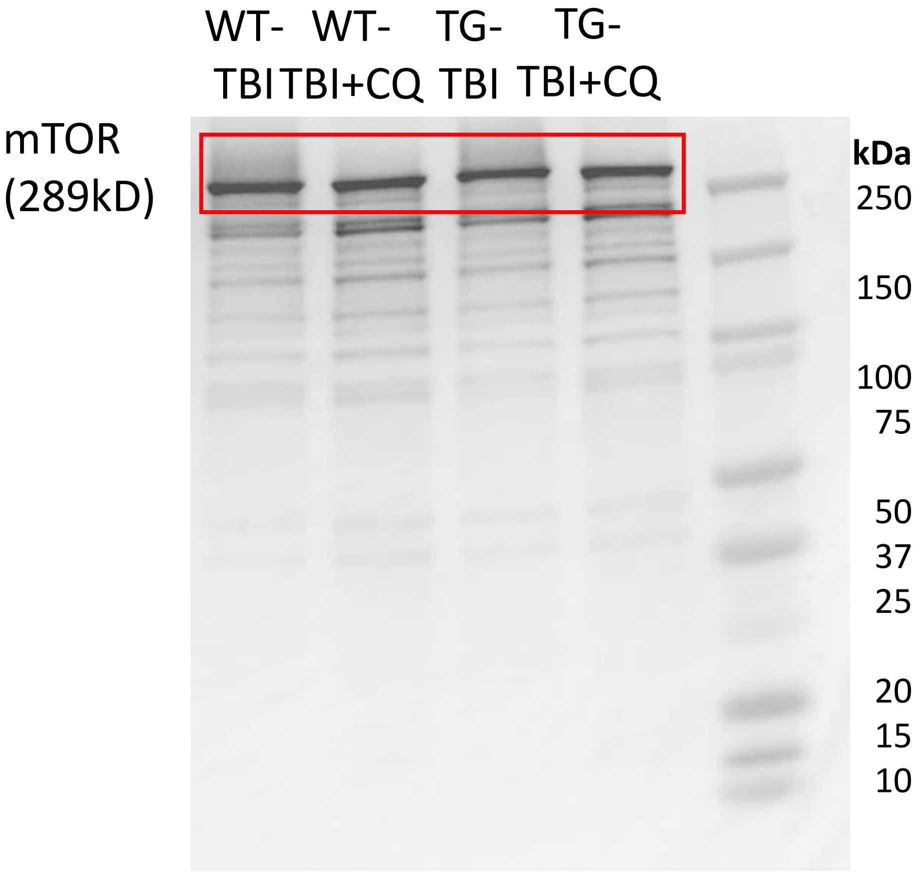

On 4-20% SDS-PAGE stripped after "**p-mTOR/mTOR after TBI +CQ 3d**"

p-Akt/Akt after TBI +CQ 3d

Full unedited gel for **Figure.9D**

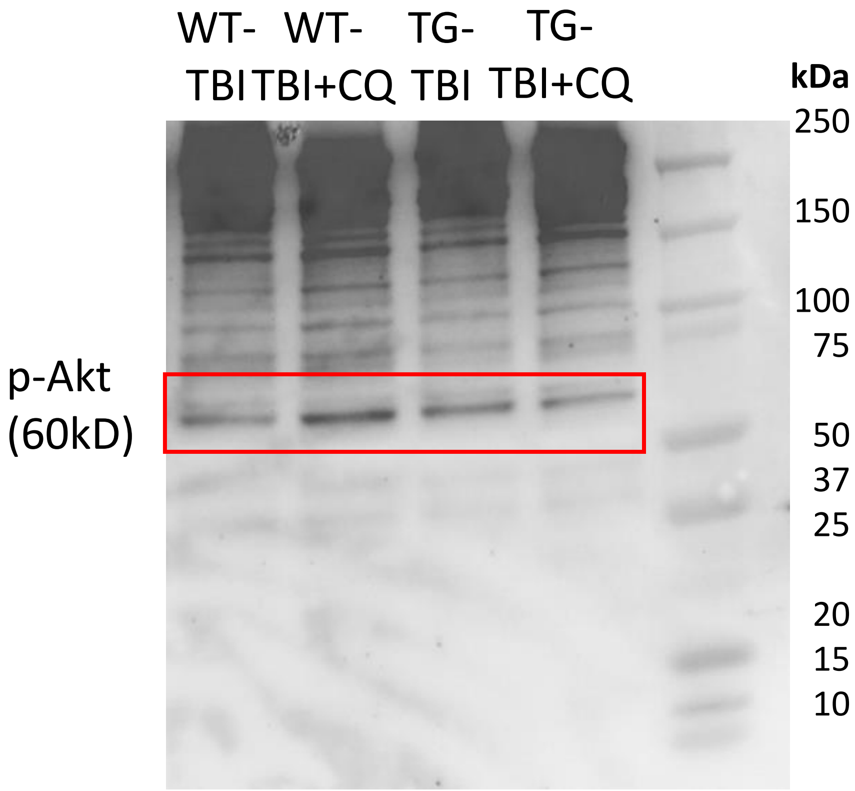

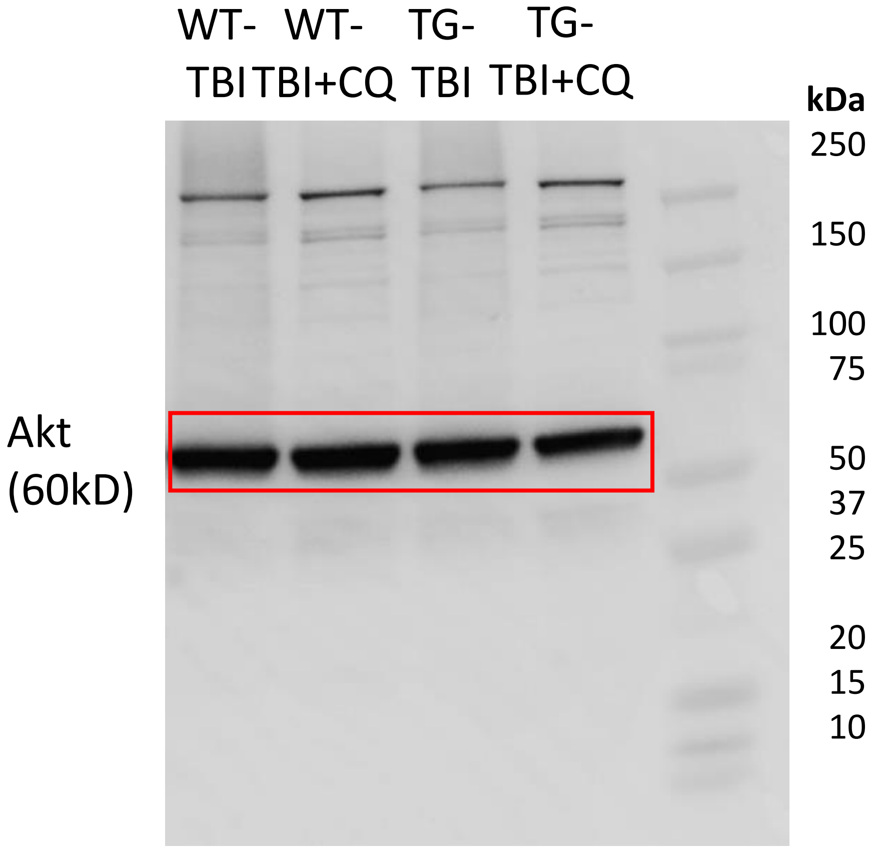

On 4-20% SDS-PAGE stripped after "**p-mTOR/mTOR after TBI +CQ 3d**"

GAP43 after TBI +CQ 3d Full unedited gel for **Figure.9D**

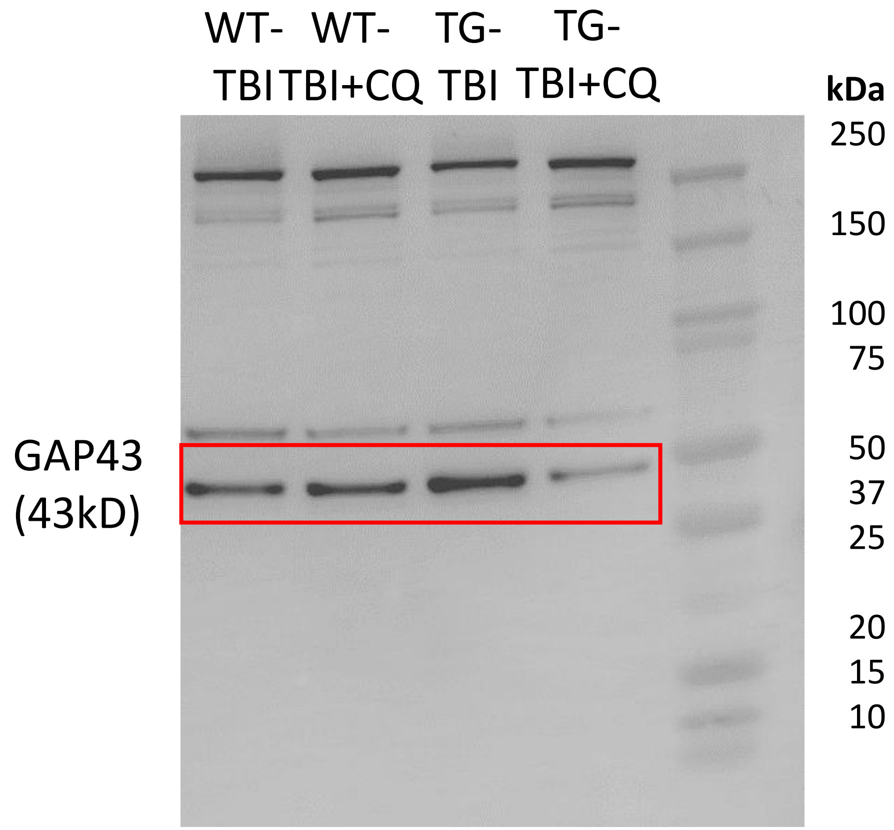

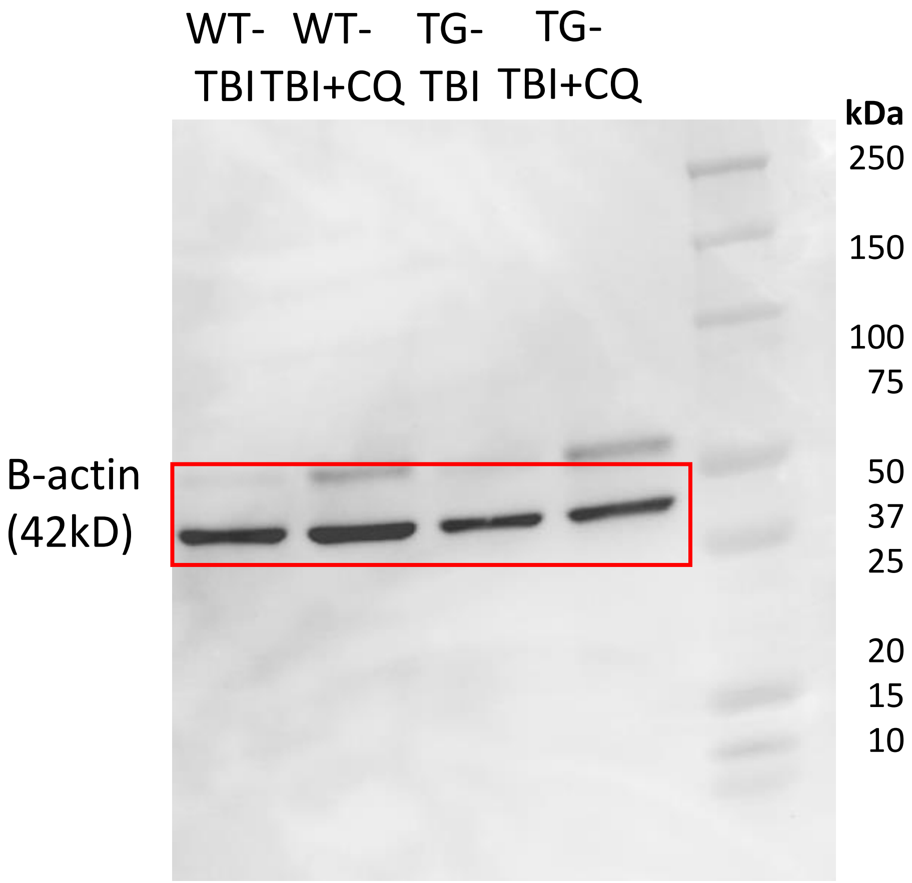

On 4-20% SDS-PAGE stripped after "**p-mTOR/mTOR after TBI +CQ 3d**"

BAG3 after TBI +CQ 3d

Full unedited gel for **Figure.10B**

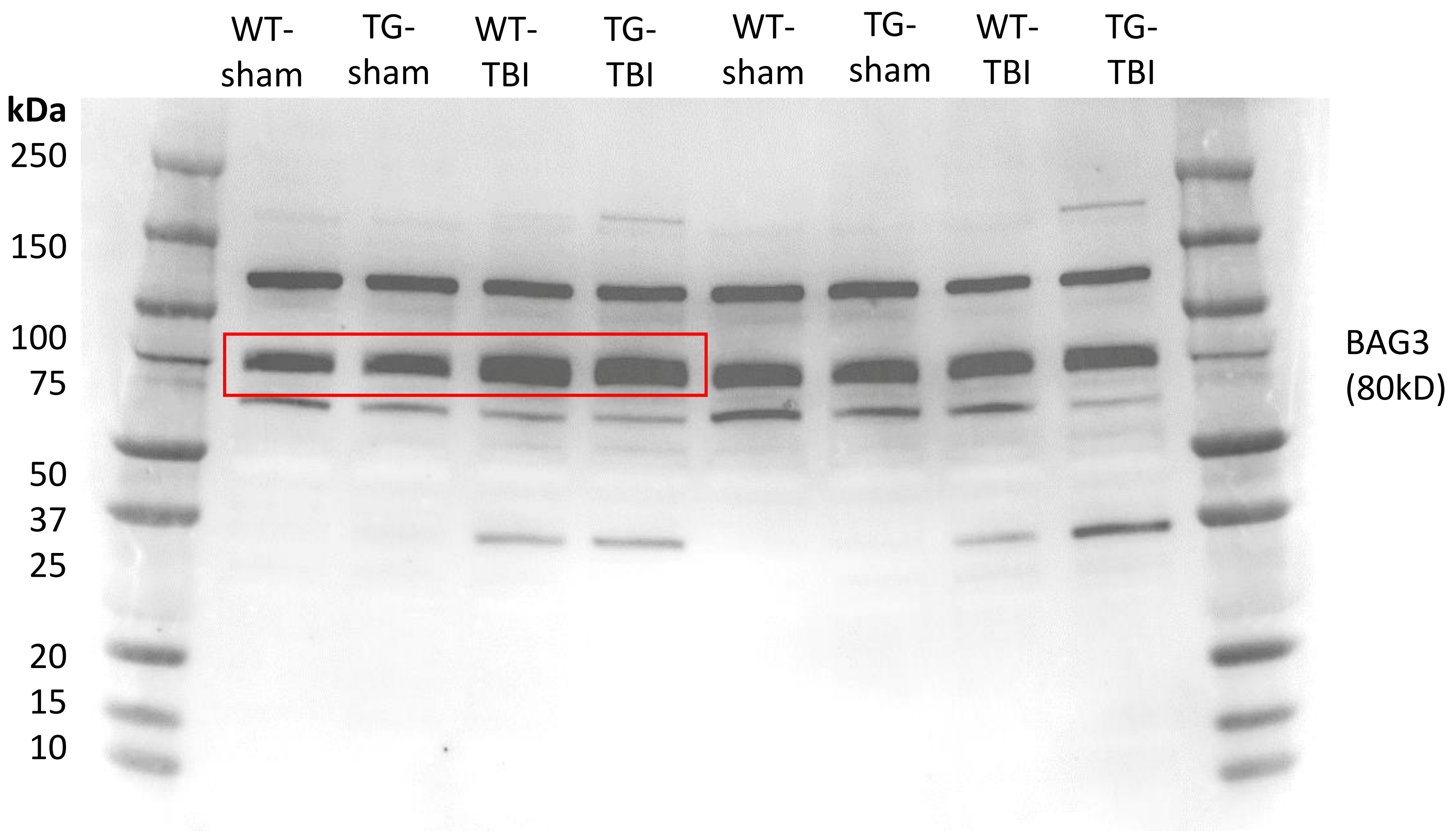

On 4-20% SDS-PAGE

BAG3 after TBI +CQ 3d

Full unedited gel for **Figure.10B**

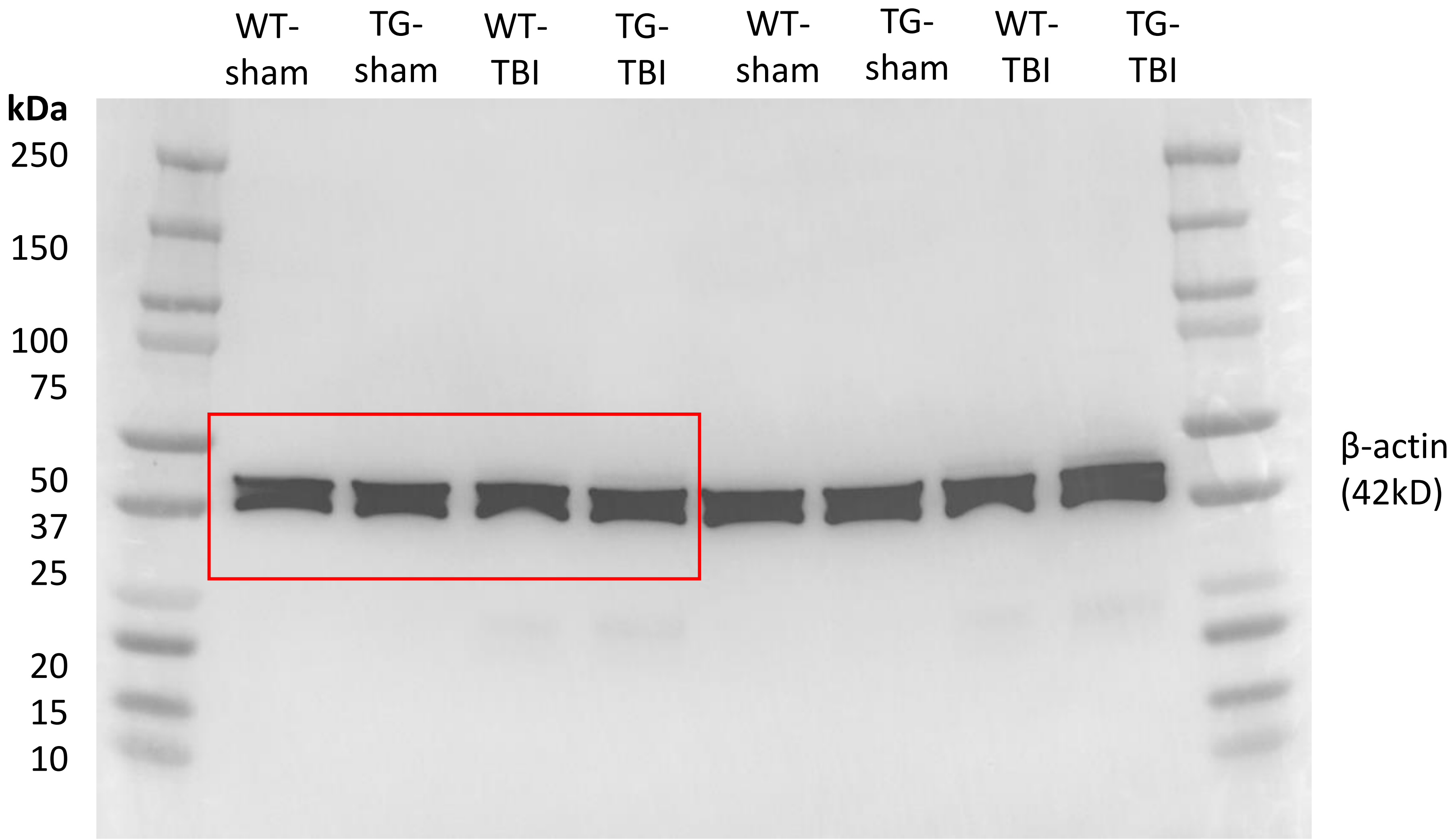

On 4-20% SDS-PAGE

stripped after "**BAG3 after TBI +CQ 3d**"

CoIP——βAPP

Full unedited gel for **Figure.10D**

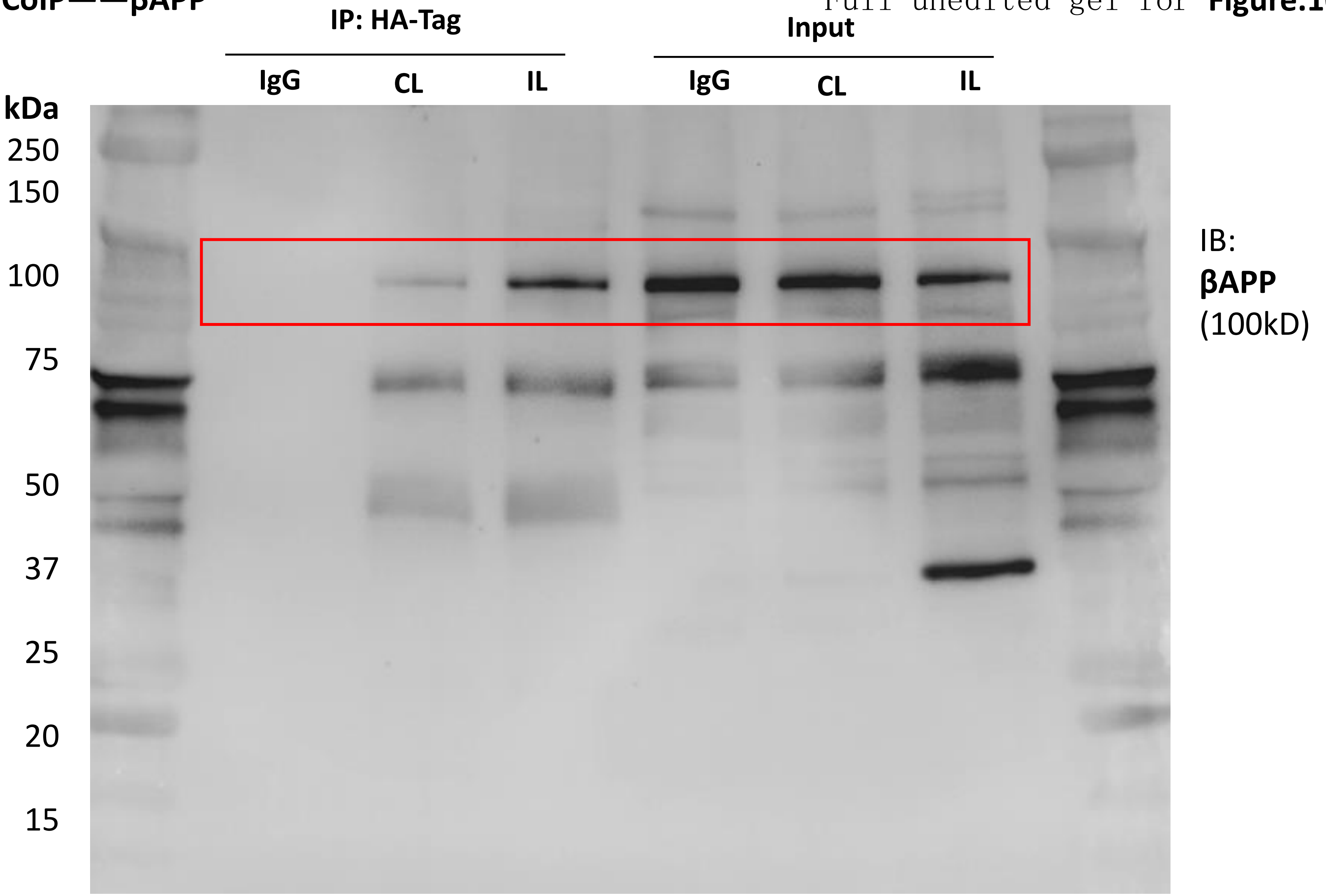

On 4-20% SDS-PAGE

CoIP——BAG3

Full unedited gel for **Figure.10D**

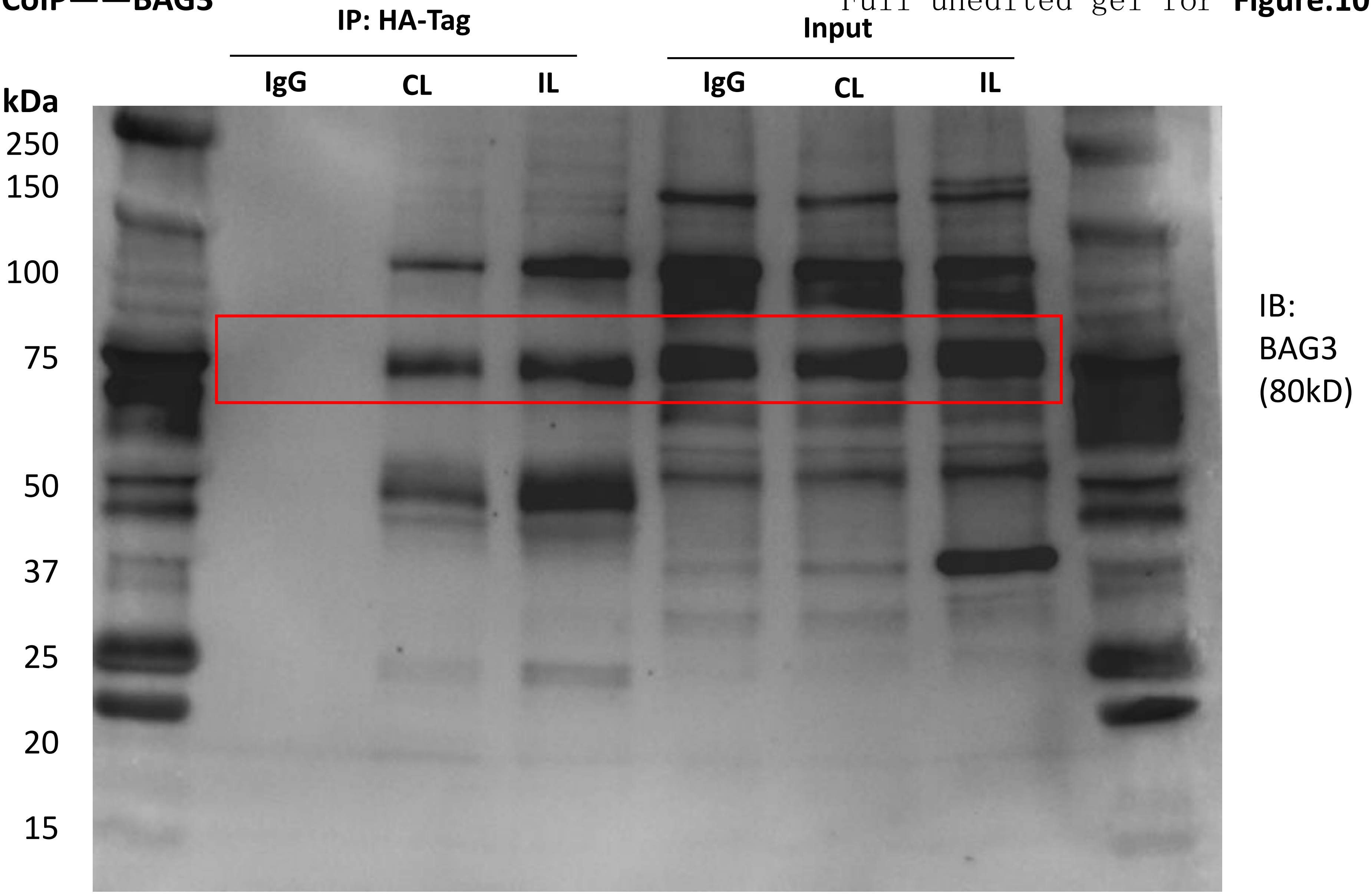

On 4-20% SDS-PAGE stripped after "**CoIP——βAPP**"

CoIP——SQSTM1 Full unedited gel for **Figure.10D**

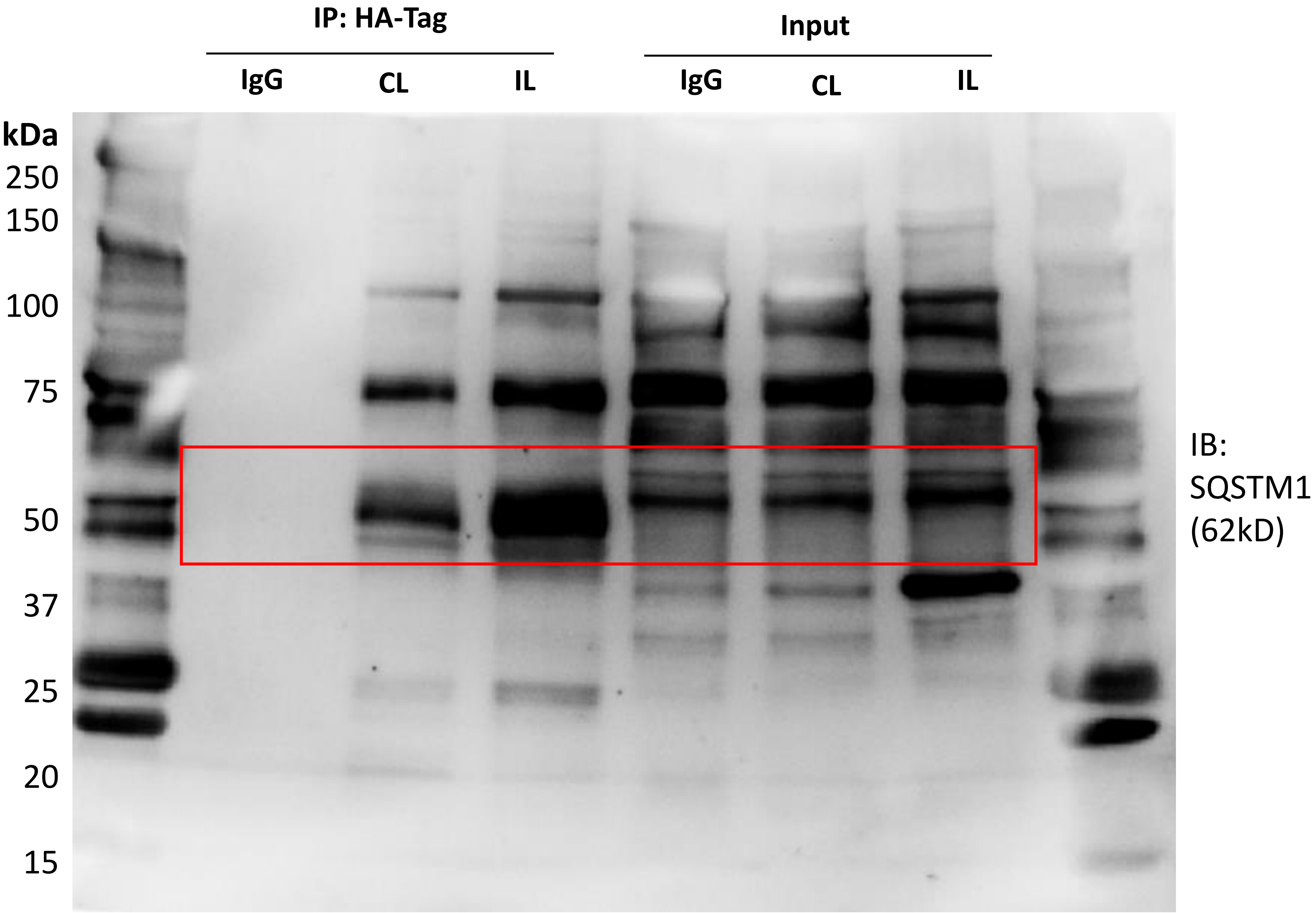

On 10% SDS-PAGE stripped after “**CoIP——BAG3**”

CoIP——HA-Tag Full unedited gel for **Figure.10D**

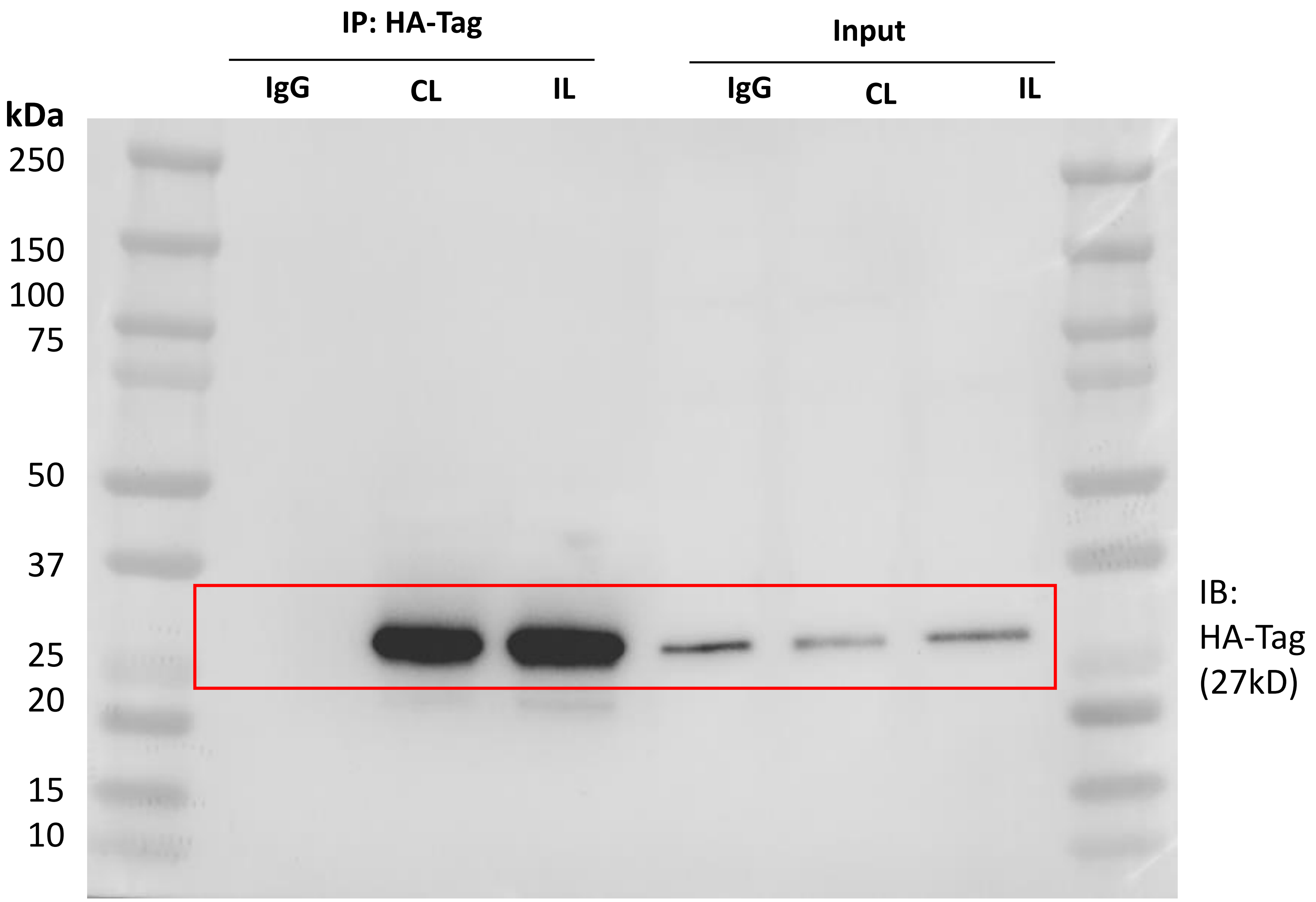

On 10% SDS-PAGE stripped after "**CoIP——SQSTM1**"

CoIP——Akt

Full unedited gel for **Figure.10D**

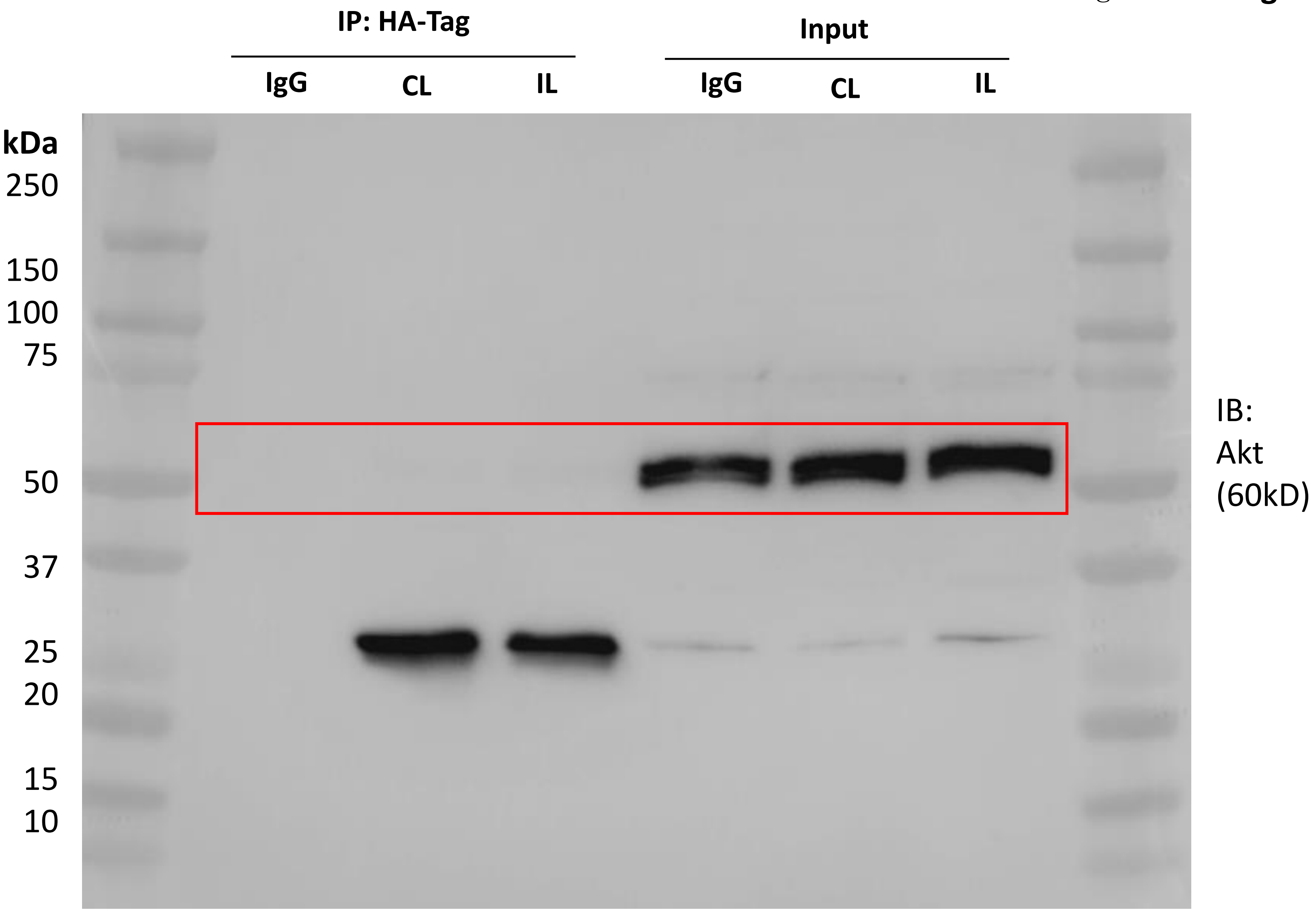

On 10% SDS-PAGE   stripped after “**CoIP——HA-Tag**”

BAG3 after OGD+shBAG3+4-OHT Full unedited gel for **Figure.11F**

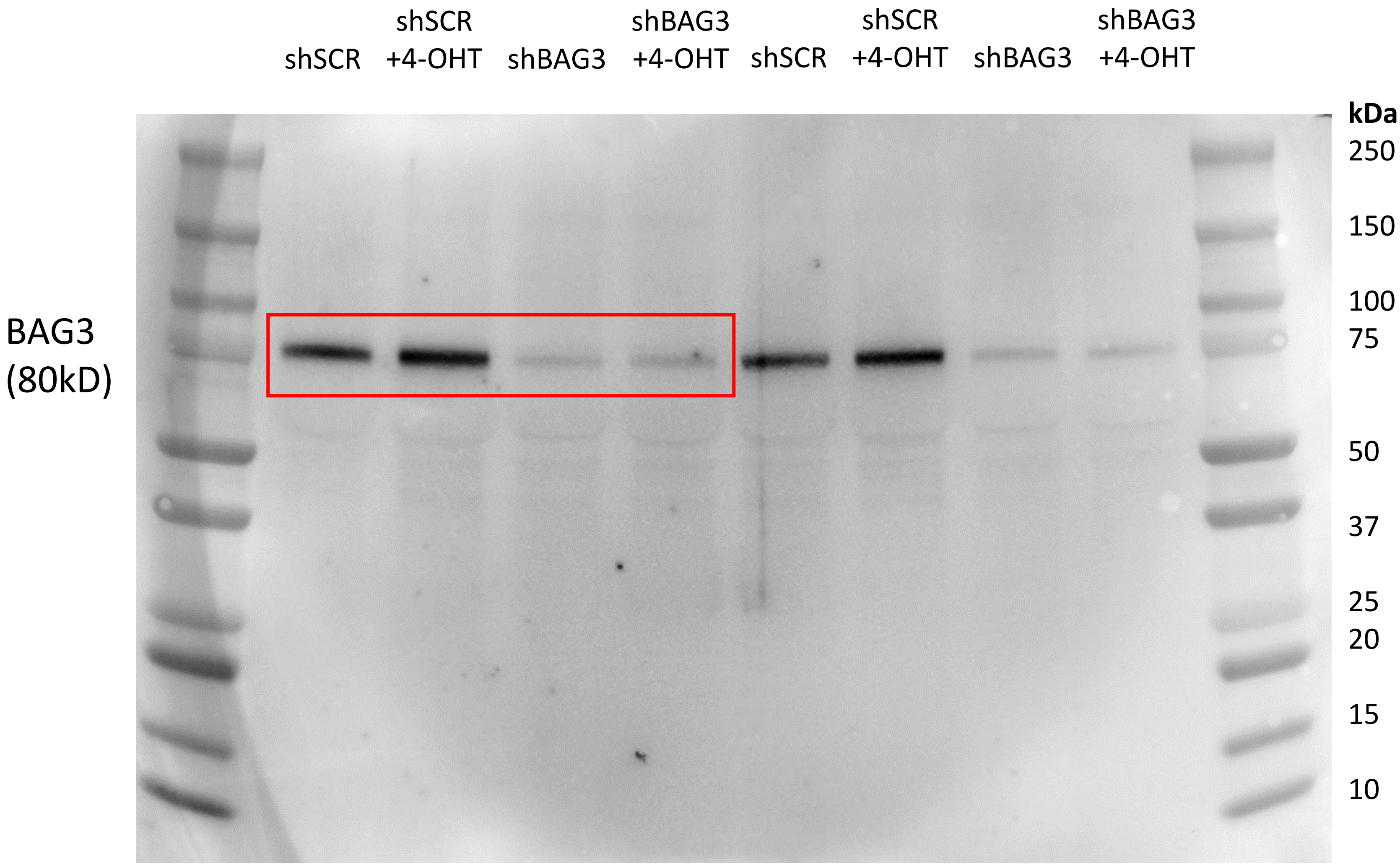

On 4-20% SDS-PAGE

SQSTM1 after OGD+shBAG3+4-OHT Full unedited gel for **Figure.11F**

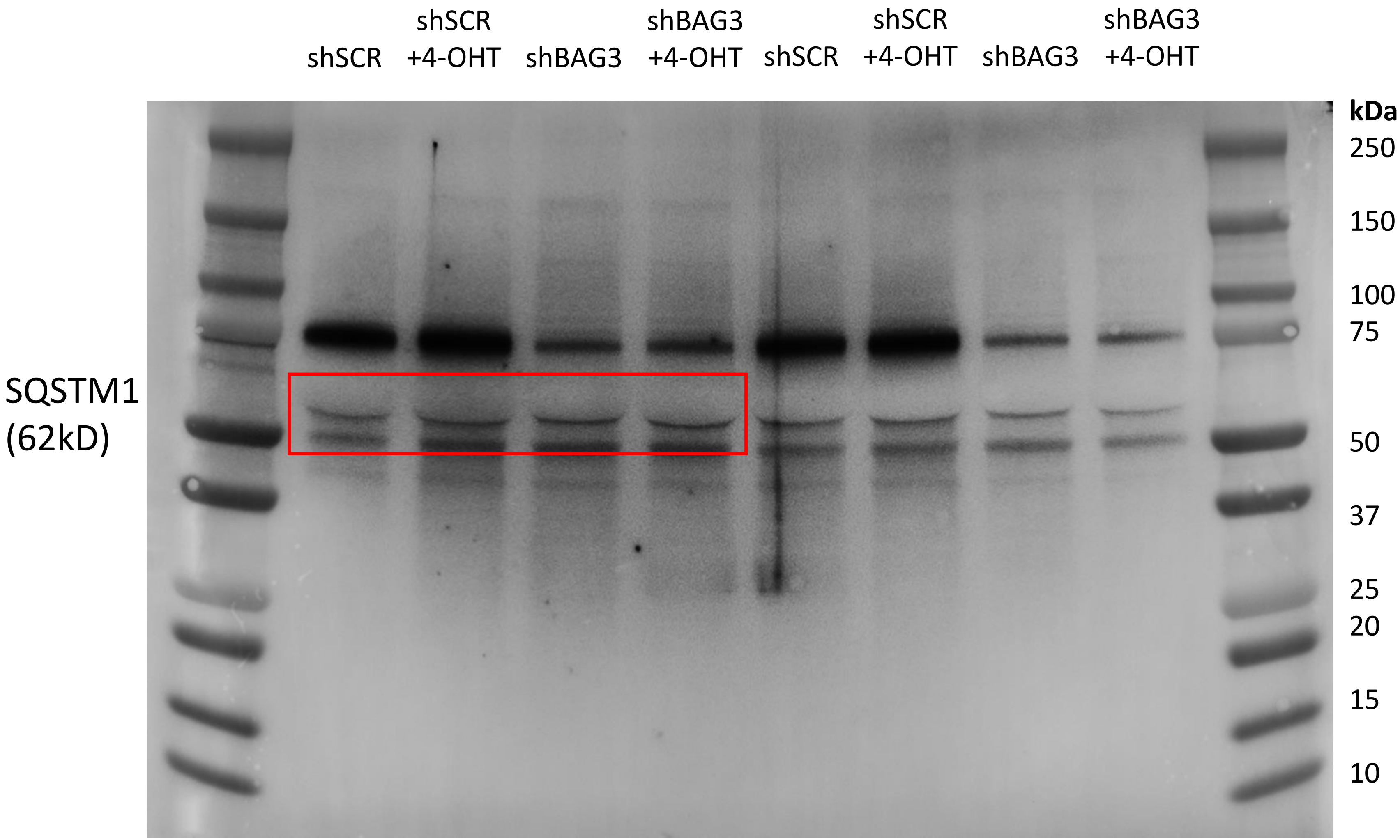

On 4-20% SDS-PAGE stripped after "**BAG3 after OGD+shBAG3+4-OHT**"

HSPB2 after OGD+shBAG3+4-OHT Full unedited gel for **Figure.11F**

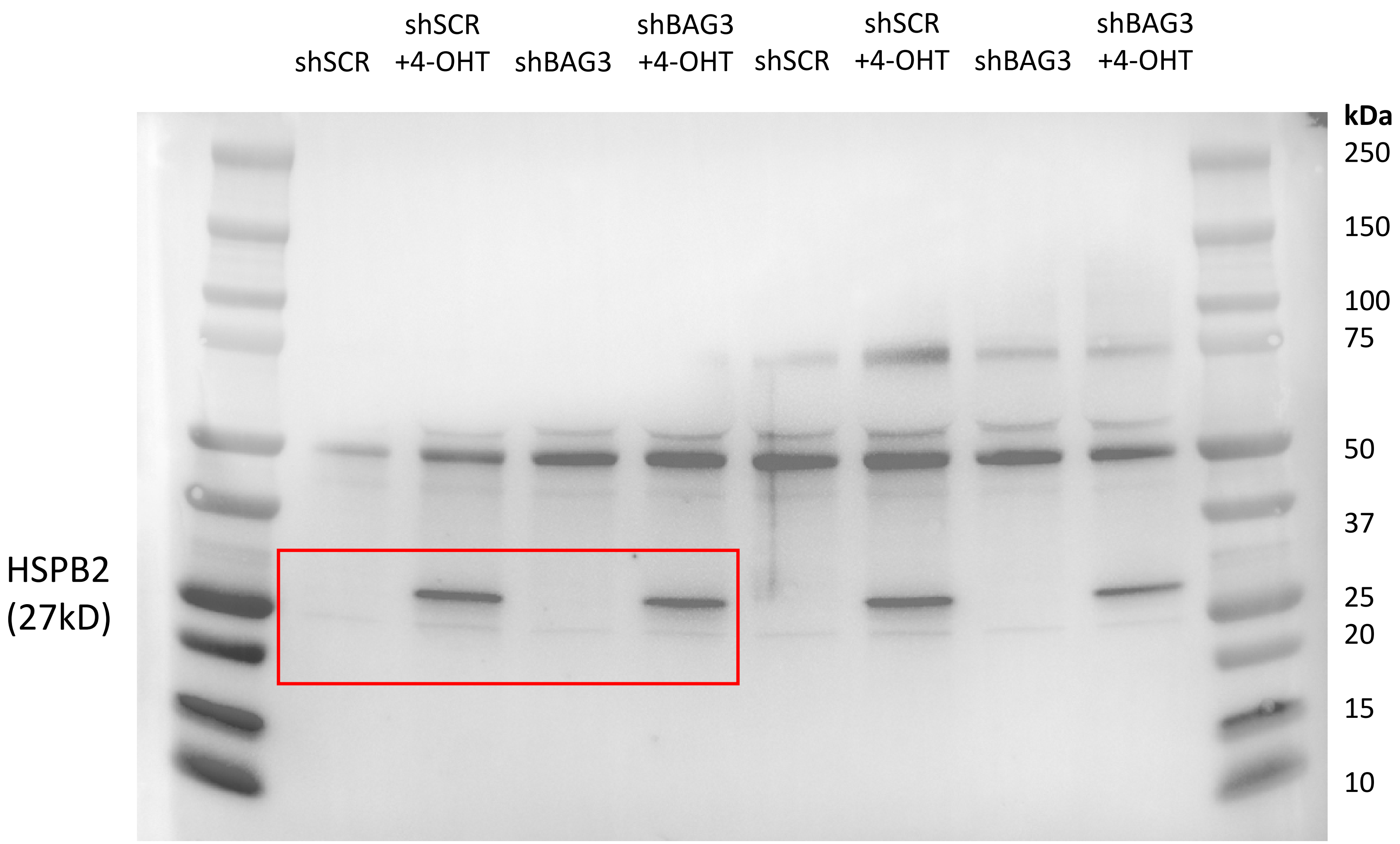

On 4-20% SDS-PAGE stripped after "**SQSTM1 after OGD+shBAG3+4-OHT**"

LC3 after OGD+shBAG3+4-OHT Full unedited gel for **Figure.11F**

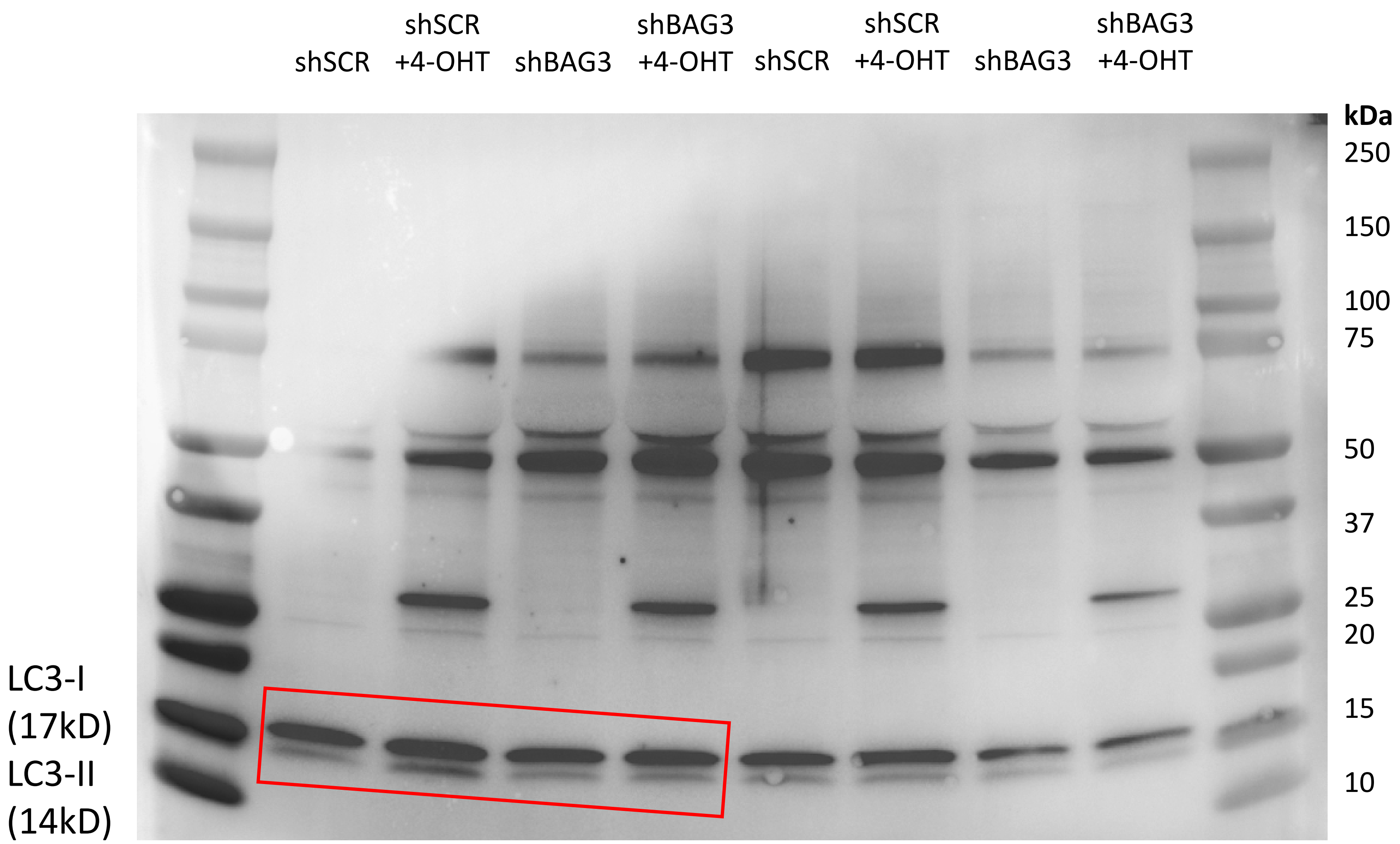

On 4-20% SDS-PAGE stripped after “**HSPB2 after OGD+shBAG3+4-OHT**”

β-actin after OGD+shBAG3+4-OHT Full unedited gel for **Figure.11F**

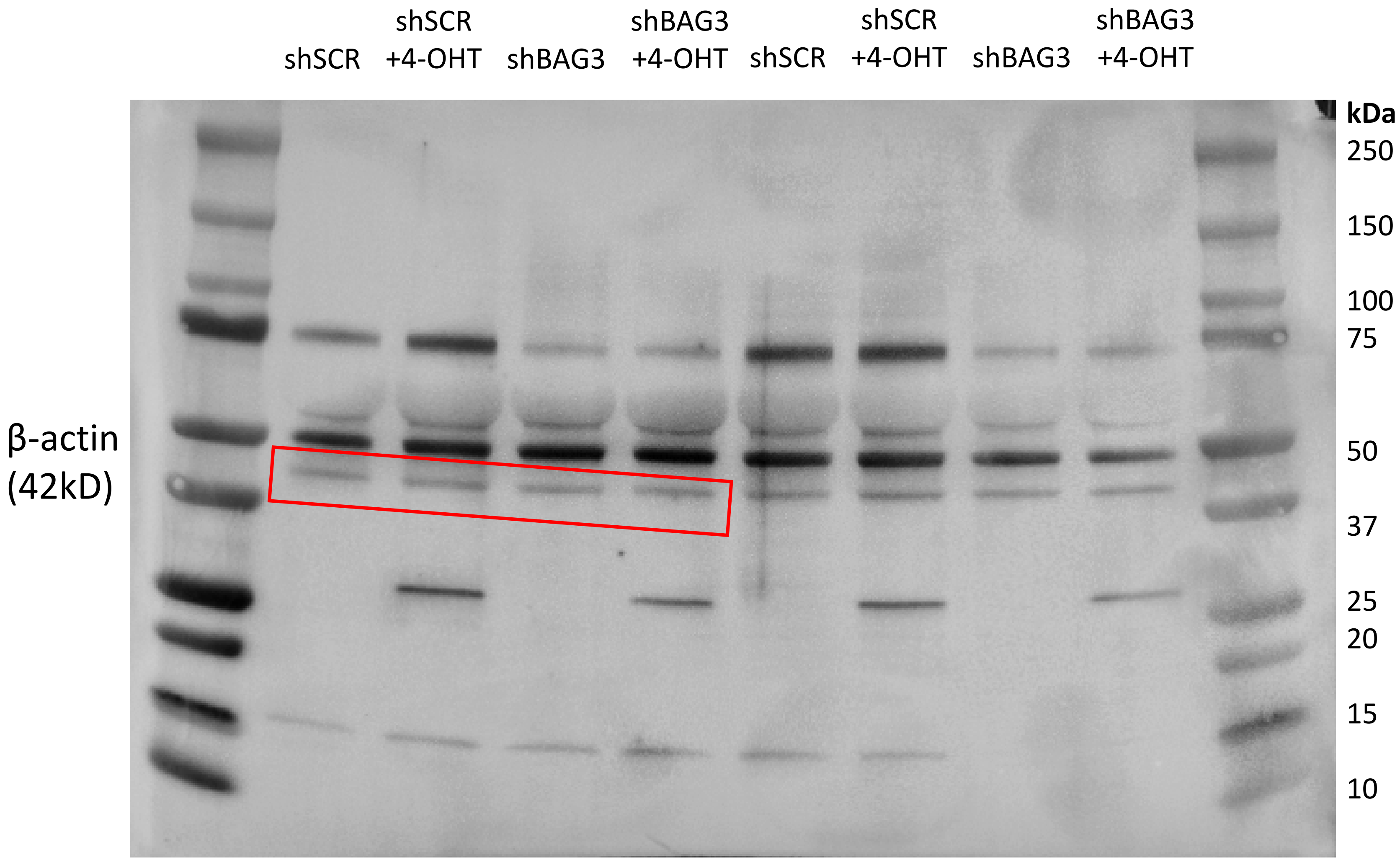

On 4-20% SDS-PAGE stripped after “**LC3 after OGD+shBAG3+4-OHT**”

Lysate HSPB2 after OGD

Full unedited gel for **Figure.S1I**

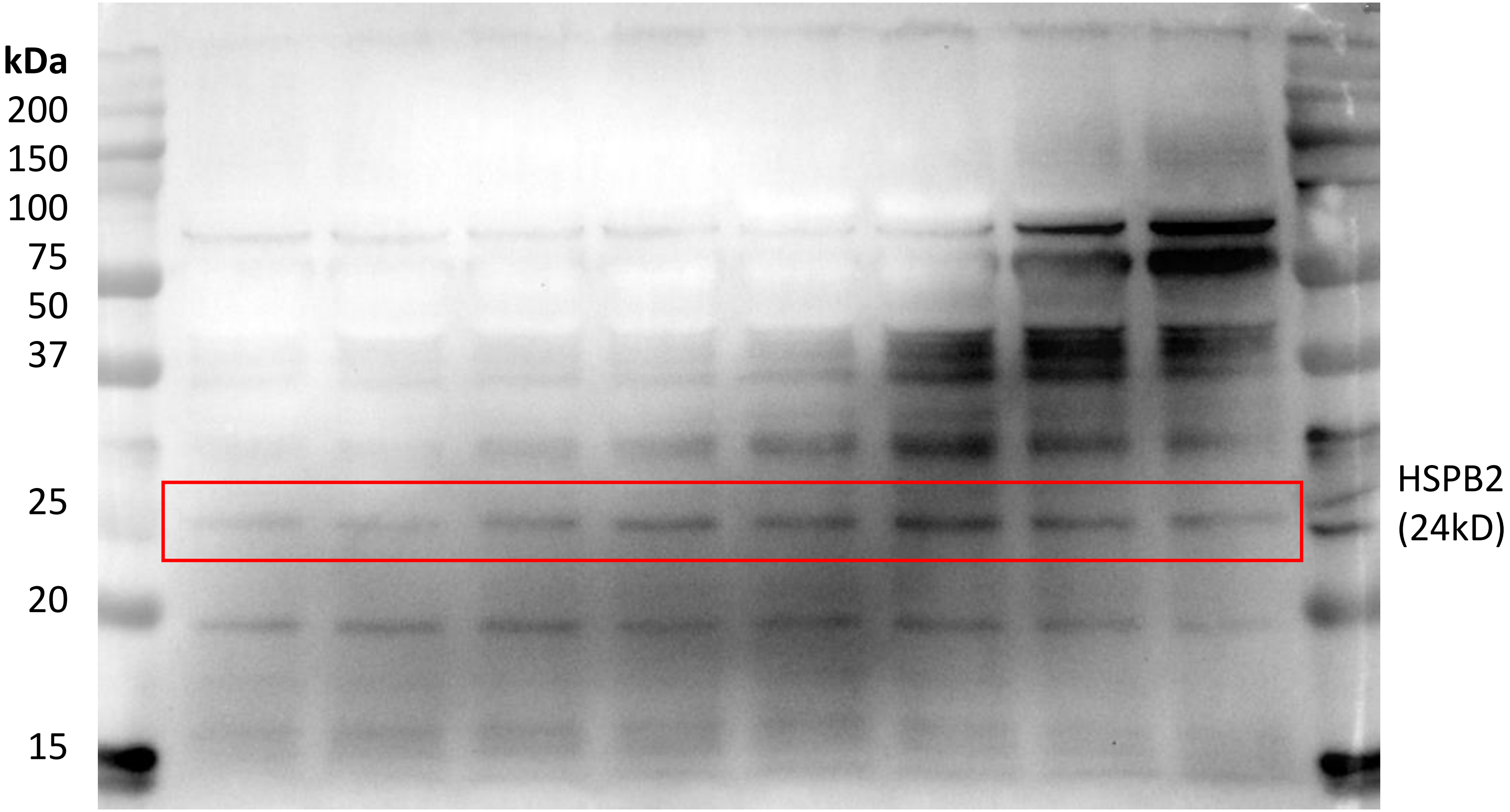

On 12% SDS-PAGE

Lysate HSPB2 after OGD

Full unedited gel for **Figure.S1I**

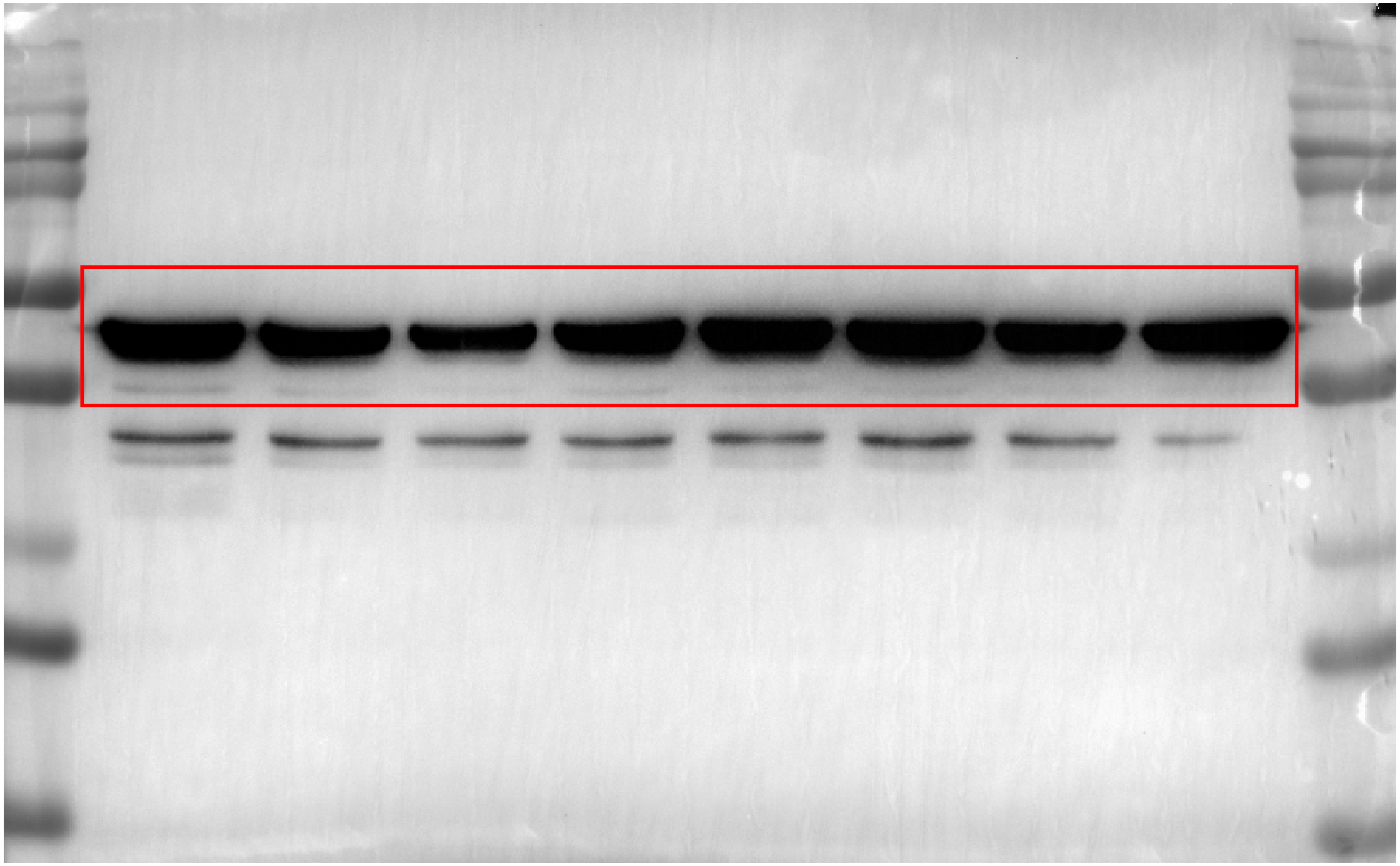

On 12% SDS-PAGE

stripped after "**Lysate HSPB2 after OGD**"

HSPB2 overexpression

Full unedited gel for **Figure.S2G**

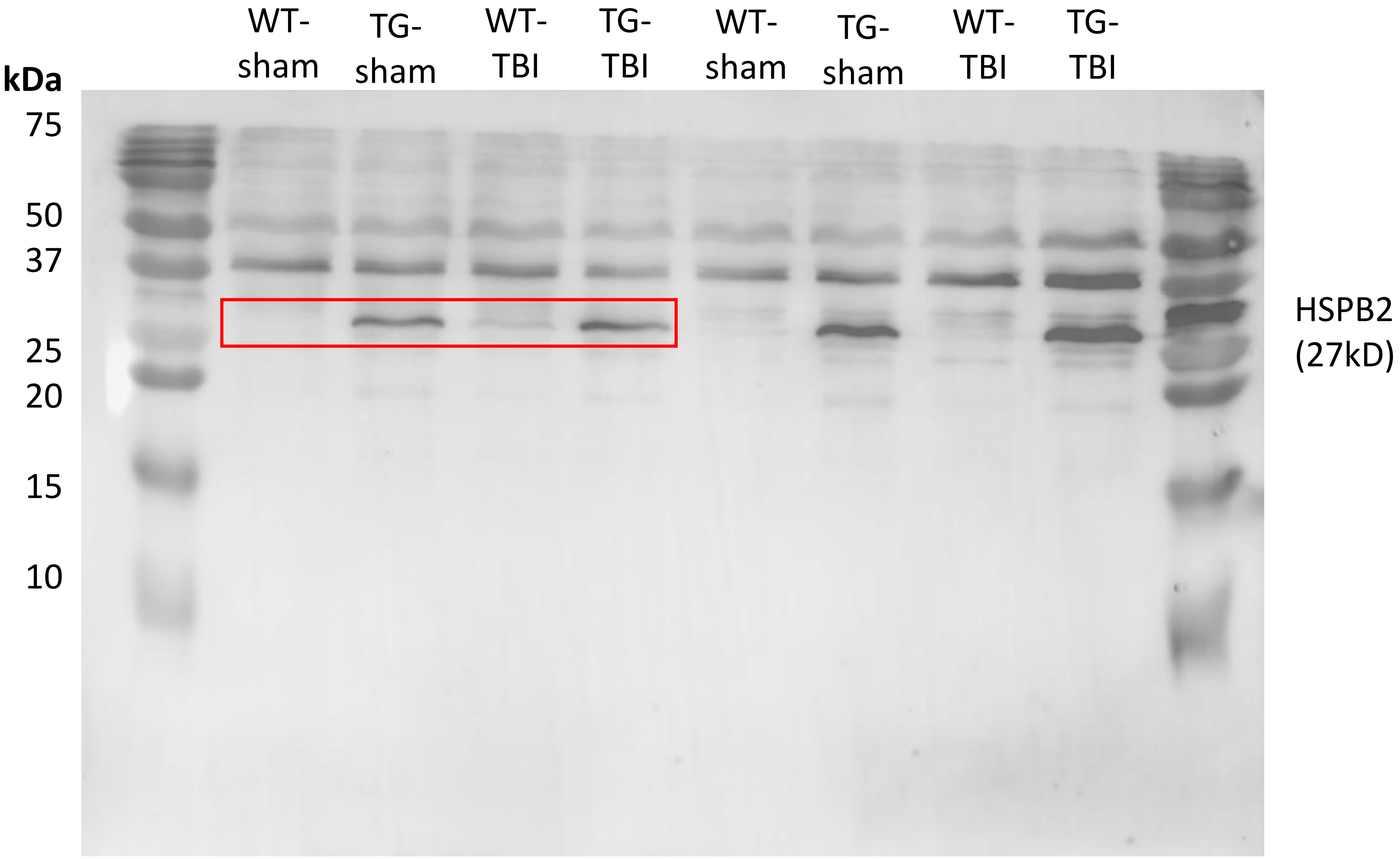

On 15% SDS-PAGE

HSPB2 overexpression

Full unedited gel for **Figure.S2G**

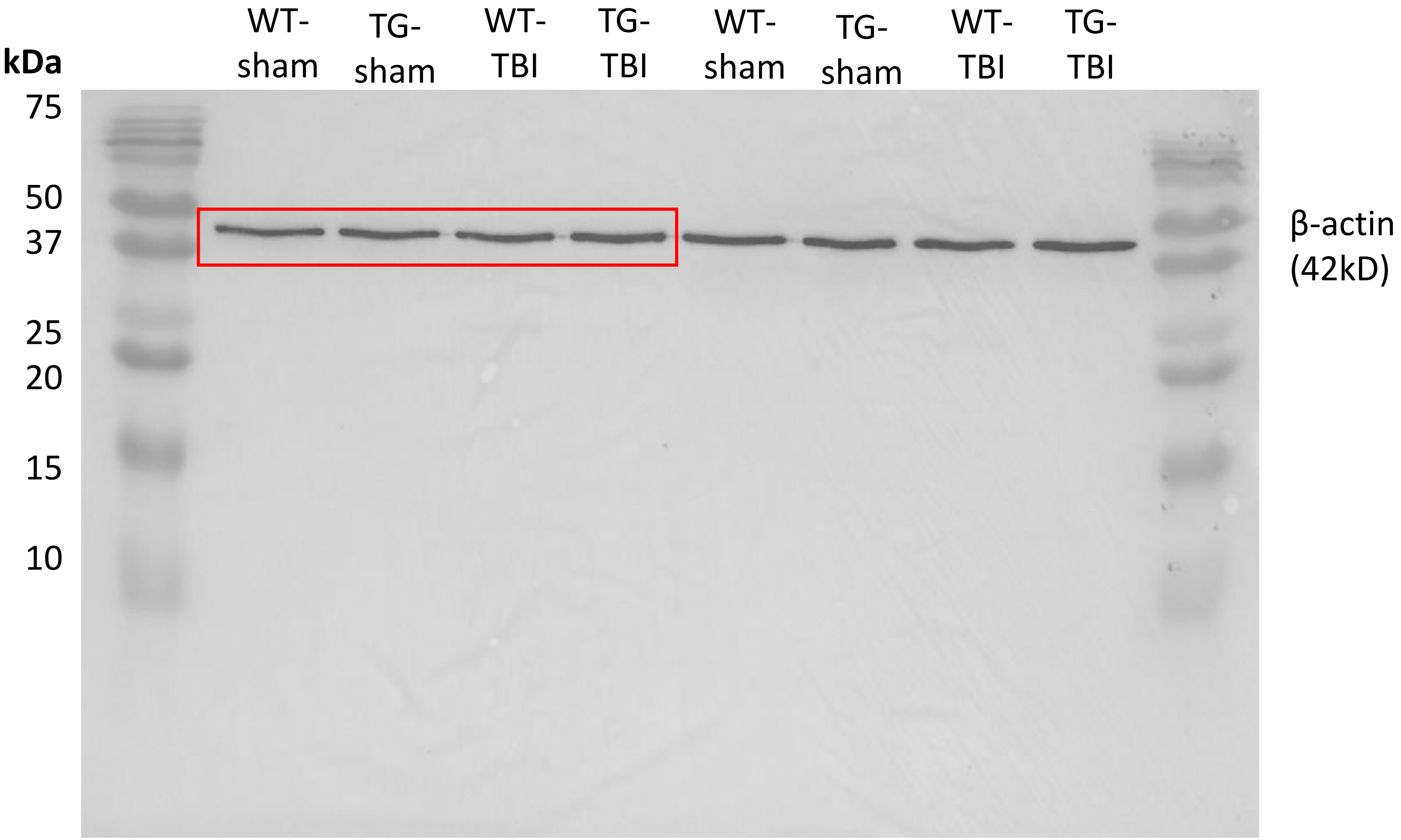

On 15 % SDS-PAGE

HSPB2 overexpression – HA-Tag Full unedited gel for **Figure.S2G**

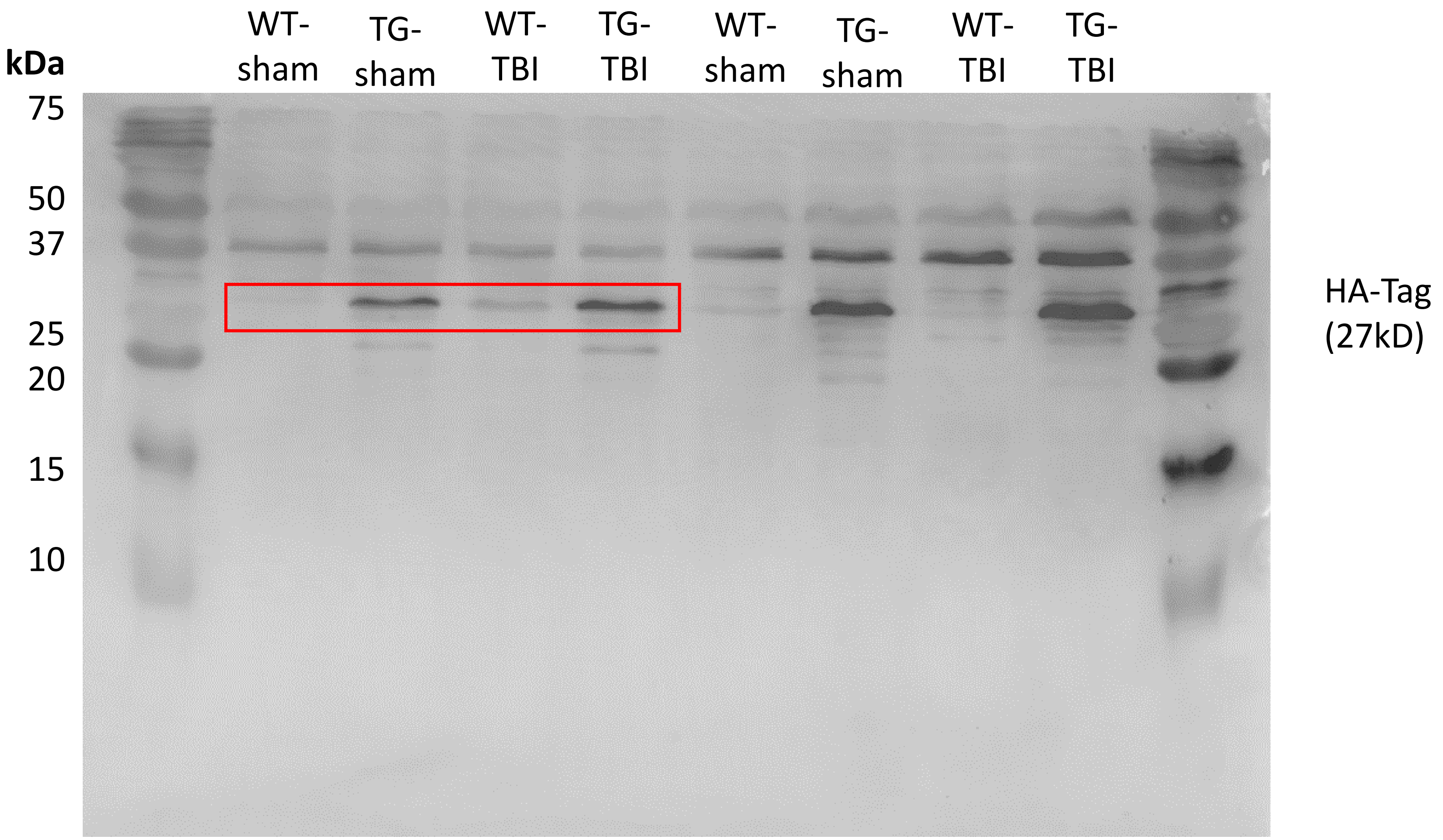

On 15 % SDS-PAGE stripped after "**HSPB2 overexpression**"
