# Supplementary material for: HSPB2 facilitates neural regeneration through autophagy for sensorimotor recovery after traumatic brain injury
Source: JCI Insight. 2023 Aug 22;8(16):e168919. doi: 10.1172/jci.insight.168919 (PMC10543718; doi:10.1172/jci.insight.168919)
Supplement: Supplemental table 4 [file jciinsight-8-168919-s082.pdf]

Table S4. Statistical analysis

FIGURE	n	DATA STRUCTURE	TEST USED	STATISTIC	P VALUE
1C.CTX	3	Normal distribution	One-way RM ANOVA, Bonferroni post hoc	F (4, 10) = 16.68	sham vs. 1d = 0.5215<br>sham vs. 3d = 0.0008<br>sham vs. 5d >0.9999<br>sham vs. 7d >0.9999
1C.HIP	3	Normal distribution	One-way RM ANOVA, Bonferroni post hoc	F (4, 10) = 3.240	sham vs. 1d = 0.1482<br>sham vs. 3d >0.9999<br>sham vs. 5d >0.9999<br>sham vs. 7d >0.9999
1E.CTX	6	Normal distribution	One-way RM ANOVA, Bonferroni	F (3, 20) = 8.241	sham vs. 1d = 0.0871<br>sham vs. 3d = 0.0005<br>sham vs. 7d = 0.2791
1E.HIP	6	Normal distribution	One-way RM ANOVA, Bonferroni post hoc	F (3, 20) = 6.503	sham vs. 1d = 0.3738<br>sham vs. 3d = 0.0044<br>sham vs. 7d >0.9999
1G	6	Normal distribution	Two-way RM ANOVA, Bonferroni post hoc	F (2, 15) = 114.7	sham:<br>NeuN+ vs.Iba1+ <0.0001<br>NeuN+ vs. GFAP+ <0.0001<br>TBI:<br>NeuN+ vs.Iba1+ <0.0001<br>NeuN+ vs. GFAP+ <0.0001<br>Sham vs. TBI:<br>NeuN+ = 0.0002<br>Iba1+ = 0.0320<br>GFAP+ = 0.0007
2C	3	Normal distribution	Unpaired t test, two-tailed		0.0296
3B. Body curl	WT-sham = 7<br>TG-sham = 6<br>WT-TBI = 15<br>TG-TBI = 17	Normal distribution	Two-way RM ANOVA, Bonferroni post hoc	F (3, 41) = 60.83	TG-sham vs. WT-sham >0.9999<br>WT-TBI vs. WT-sham <0.0001<br>TG-TBI vs. TG-sham <0.0001<br>TG-TBI vs. WT-TBI <0.0001<br>TG-TBI vs. WT-TBI:<br>d21 = 0.0001<br>d28 <0.0001<br>d35 <0.0001
3B. Grid-walking	WT-sham = 5<br>TG-sham = 7<br>WT-TBI = 14<br>TG-TBI = 16	Normal distribution	Two-way RM ANOVA, Bonferroni post hoc	F (3, 38) = 152.5	TG-sham vs. WT-sham = 0.3492<br>WT-TBI vs. WT-sham <0.0001<br>TG-TBI vs. TG-sham <0.0001<br>TG-TBI vs. WT-TBI =

					0.0001<br>TG-TBI vs. WT-TBI:<br>d3 = 0.0003<br>d5 = 0.0306<br>d7 = 0.0051<br>d14 = 0.0035<br>d35 = 0.0109
3B. Rotarod	WT-sham = 7<br>TG-sham = 7<br>WT-TBI = 15<br>TG-TBI = 17	Normal distribution	Two-way RM ANOVA, Bonferroni post hoc	F (3, 42) = 46.56	TG-sham vs. WT-sham >0.9999<br>WT-TBI vs. WT-sham <0.0001<br>TG-TBI vs. TG-sham <0.0001<br>TG-TBI vs. WT-TBI = 0.0023<br>TG-TBI vs. WT-TBI:<br>d5 = 0.0238<br>d14 = 0.0397<br>d21 = 0.0379<br>d35 =0.0184
3D. 3d	WT-TBI = 6<br>TG-TBI = 5	Normal distribution	Unpaired t test, two-tailed		0.5694
3D. 35d	5	Normal distribution	Unpaired t test, two-tailed		0.0236
3D. 56d	WT-TBI = 6<br>TG-TBI = 7	Normal distribution	Unpaired t test, two-tailed		0.0426
4C. FA of EC	5	Normal distribution	Two-way RM ANOVA, Bonferroni post hoc	F (1, 8) = 24.50	TG-TBI vs. WT-TBI = 0.0011<br>d7 = 0.0202<br>d28 = 0.0001<br>d49 = 0.0431<br>TG-TBI:<br>7d vs. 28d = 0.0383<br>7d vs. 49d = 0.0222
4C. FA of STR	5	Normal distribution	Two-way RM ANOVA, Bonferroni post hoc	F (1, 8) = 29.58	TG-TBI vs. WT-TBI = 0.0006<br>d7 = 0.6026<br>d28 = 0.0128<br>d49 = 0.0598<br>TG-TBI:<br>7d vs. 49d = 0.0048
4E	5	Normal distribution	Two-way RM ANOVA, Bonferroni post hoc	F (1, 8) = 13.22	TG-TBI vs. WT-TBI = 0.0066<br>d7 = 0.1648<br>d28 = 0.0514<br>d49 = 0.0428

4F	10	Normal distribution	Pearson correlation	r = -0.7576	0.0111
4H. N1	WT-sham = 6<br>TG-sham = 6<br>WT-TBI = 6<br>TG-TBI = 7	Normal distribution	Two-way RM ANOVA, Bonferroni post hoc	F (3, 21) = 26.31	TG-sham vs. WT-sham = 0.0011<br>WT-TBI vs. WT-sham <0.0001<br>TG-TBI vs. TG-sham = 0.0005<br>TG-TBI vs. WT-TBI = 0.6362
4H. N2	WT-sham = 6<br>TG-sham = 6<br>WT-TBI = 6<br>TG-TBI = 7	Normal distribution	Two-way RM ANOVA, Bonferroni post hoc	F (3, 21) = 13.66	TG-sham vs. WT-sham = 0.0047<br>WT-TBI vs. WT-sham <0.0001<br>TG-TBI vs. TG-sham = 0.0995<br>TG-TBI vs. WT-TBI = 0.0362
4I	10	Normal distribution	Pearson correlation	r = -0.6322	0.0280
5E	5	Normal distribution	One-way RM ANOVA, Bonferroni post hoc	F (3, 16) = 23.27	TG-sham vs. WT-sham >0.9999<br>WT-TBI vs. WT-sham = 0.0156<br>TG-TBI vs. TG-sham <0.0001<br>TG-TBI vs. WT-TBI = 0.0156
5G	5	Normal distribution	Unpaired t test, two-tailed		0.0129
6C	WT-sham = 9<br>TG-sham = 9<br>2m:<br>WT-TBI = 6<br>TG-TBI = 6<br>3m:<br>WT-TBI = 15<br>TG-TBI = 15	Normal distribution	Two-way Mix-effects ANOVA, Bonferroni post hoc	F (1, 28) = 10.14	WT-TBI vs. TG-TBI:<br>sham >0.9999<br>2m = 0.0019<br>3m = 0.0288<br>WT-TBI:<br>Sham vs. 2m = 0.0135<br>Sham vs. 3m = 0.0225<br>TG-TBI:<br>Sham vs. 2m = 0.0056<br>Sham vs. 3m = 0.0007
6D	10	Normal distribution	Pearson correlation	r = -0.6573	0.0389
7A	3	Normal distribution	Unpaired t test, two-tailed		0.0365
7D. SQSTM1	4	Normal distribution	One-way RM ANOVA, Bonferroni post hoc	F (3, 12) = 46.32	TG-sham vs. WT-sham = 0.0179<br>WT-TBI vs. WT-sham <0.0001<br>TG-TBI vs. TG-sham <0.0001<br>TG-TBI vs. WT-TBI = 0.0122
7D.LC3	4	Normal distribution	One-way RM	F (3, 12) = 14.82	TG-sham vs. WT-sham = 0.1145

			ANOVA, Bonferroni post hoc		WT-TBI vs. WT-sham = 0.0026<br>TG-TBI vs. TG-sham = 0.0009<br>TG-TBI vs. WT-TBI = 0.0365
7E. SQSTM1	4	Normal distribution	One-way RM ANOVA, Bonferroni post hoc	F (3, 12) = 30.29	WT-TBI vs. TG-TBI = 0.0012<br>WT-TBI vs. WT-TBI+CQ = 0.0008<br>TG-TBI vs. TG-TBI +CQ = 0.0074<br>TG-TBI+CQ vs. WT-TBI+CQ = 0.0118
7E. LC3	4	Normal distribution	One-way RM ANOVA, Bonferroni post hoc	F (3, 16) = 9.044	WT-TBI vs. TG-TBI >0.9999<br>WT-TBI vs. WT-TBI+CQ = 0.0013<br>TG-TBI vs. TG-TBI +CQ = 0.0386<br>TG-TBI+CQ vs. WT-TBI+CQ >0.9999
8C. Autophagosome(non-CQ)	WT-sham = 25<br>TG-sham = 25<br>WT-TBI = 24<br>TG-TBI = 24	Normal distribution	One-way RM ANOVA, Bonferroni post hoc	F (3, 94) = 28.00	TG-sham vs. WT-sham = 0.0099<br>WT-TBI vs. WT-sham = 0.0001<br>TG-TBI vs. TG-sham <0.0001<br>TG-TBI vs. WT-TBI = 0.0001
8C. Autophagosome(CQ)	WT-sham+CQ = 25<br>TG-sham+CQ = 24<br>WT-TBI+CQ = 25<br>TG-TBI+CQ = 23	Normal distribution	One-way RM ANOVA, Bonferroni post hoc	F (3, 92) = 33.94	TG-sham+CQ vs. WT-sham +CQ = 0.0563<br>WT-TBI+CQ vs. WT-sham+CQ <0.0001<br>TG-TBI+CQ vs. TG-sham+CQ <0.0001<br>TG-TBI+CQ vs. WT-TBI+CQ >0.9999
8C. Autophagosome(CQ vs. non-CQ)	WT-sham = 25<br>TG-sham = 25<br>WT-TBI = 24<br>TG-TBI = 24<br>WT-sham+CQ = 25<br>TG-sham+CQ = 24<br>WT-TBI+CQ = 25<br>TG-TBI+CQ =	Normal distribution	Unpaired t test, two-tailed		WT-sham vs. WT-sham +CQ = 0.0272<br>TG-sham vs. TG-sham +CQ = 0.0949<br>WT-TBI vs. WT-TBI +CQ <0.0001<br>TG-TBI vs. TG-TBI +CQ = 0.4528

	23				
8C. Autolysosome(non-CQ)	WT-sham = 25<br>TG-sham = 25<br>WT-TBI = 24<br>TG-TBI = 24	Normal distribution	One-way RM ANOVA, Bonferroni post hoc	F (3, 94) = 34.93	TG-sham vs. WT-sham = 0.1080<br>WT-TBI vs. WT-sham = 0.0003<br>TG-TBI vs. TG-sham <0.0001<br>TG-TBI vs. WT-TBI <0.0001
8C. Autolysosome (CQ)	WT-sham+CQ = 25<br>TG-sham+CQ = 24<br>WT-TBI+CQ = 25<br>TG-TBI+CQ = 23	Normal distribution	One-way RM ANOVA, Bonferroni post hoc	F (3, 92) = 11.15	TG-sham+CQ vs. WT-sham +CQ >0.9999<br>WT-TBI+CQ vs. WT-sham+CQ <0.0001<br>TG-TBI+CQ vs. TG-sham+CQ = 0.0488<br>TG-TBI+CQ vs. WT-TBI+CQ >0.9999
8C. Autolysosome (CQ vs. non-CQ)	WT-sham = 25<br>TG-sham = 25<br>WT-TBI = 24<br>TG-TBI = 24<br>WT-sham+CQ = 25<br>TG-sham+CQ = 24<br>WT-TBI+CQ = 25<br>TG-TBI+CQ = 23	Normal distribution	Unpaired t test, two-tailed		WT-sham vs. WT-sham +CQ = 0.0027<br>TG-sham vs. TG-sham +CQ <0.0001<br>WT-TBI vs. WT-TBI +CQ = 0.1163<br>TG-TBI vs. TG-TBI +CQ <0.0001
8C. Autophagic flux (non-CQ)	WT-sham = 25<br>TG-sham = 25<br>WT-TBI = 24<br>TG-TBI = 24	Normal distribution	One-way RM ANOVA, Bonferroni post hoc	F (3, 93) = 6.776	TG-sham vs. WT-sham >0.9999<br>WT-TBI vs. WT-sham = >0.9999<br>TG-TBI vs. TG-sham = 0.0099<br>TG-TBI vs. WT-TBI = 0.0259
8C. Autophagic flux (CQ)	WT-sham+CQ = 25<br>TG-sham+CQ = 24<br>WT-TBI+CQ = 25<br>TG-TBI+CQ = 23	Normal distribution	One-way RM ANOVA, Bonferroni post hoc	F (3, 92) = 3.052	TG-sham+CQ vs. WT-sham +CQ >0.9999<br>WT-TBI+CQ vs. WT-sham+CQ = 0.0322<br>TG-TBI+CQ vs. TG-sham+CQ >0.9999<br>TG-TBI+CQ vs. WT-TBI+CQ >0.9999
8C. Autophagic flux (CQ vs. non-CQ)	WT-sham = 25<br>TG-sham = 25<br>WT-TBI = 24	Normal distribution	Unpaired t test, two-tailed		WT-sham vs. WT-sham +CQ <0.0001<br>TG-sham vs. TG-sham +CQ <0.0001

	TG-TBI = 24<br>WT-sham+CQ = 25<br>TG-sham+CQ = 24<br>WT-TBI+CQ = 25<br>TG-TBI+CQ = 23				WT-TBI vs. WT-TBI +CQ = 0.0007<br>TG-TBI vs. TG-TBI +CQ <0.0001
9C. mTOR	4	Normal distribution	One-way RM ANOVA, Bonferroni post hoc	F (3, 12) = 29.54	TG-sham vs. WT-sham = 0.1066<br>WT-TBI vs. WT-sham = 0.0829<br>TG-TBI vs. TG-sham <0.0001<br>TG-TBI vs. WT-TBI <0.0001
9C. Akt	4	Normal distribution	One-way RM ANOVA, Bonferroni post hoc	F (3, 12) = 15.09	TG-sham vs. WT-sham = 0.0022<br>WT-TBI vs. WT-sham = 0.0044<br>TG-TBI vs. TG-sham = 0.0018<br>TG-TBI vs. WT-TBI = 0.0036
9C. GAP43	4	Normal distribution	One-way RM ANOVA, Bonferroni post hoc	F (3, 12) = 21.32	TG-sham vs. WT-sham >0.9999<br>WT-TBI vs. WT-sham = 0.5387<br>TG-TBI vs. TG-sham = 0.0022<br>TG-TBI vs. WT-TBI = 0.0036
9E. mTOR	4	Normal distribution	One-way RM ANOVA, Bonferroni post hoc	F (3, 12) = 47.20	WT-TBI vs. TG-TBI <0.0001<br>WT-TBI vs. WT-TBI+CQ = 0.0012<br>TG-TBI vs. TG-TBI +CQ = 0.0217<br>TG-TBI+CQ vs. WT-TBI+CQ = 0.0002
9E. Akt	4	Normal distribution	One-way RM	F (3, 12) = 34.46	WT-TBI vs. TG-TBI = 0.0751<br>WT-TBI vs. WT-TBI+CQ =

			ANOVA, Bonferroni post hoc		0.0036 TG-TBI vs. TG-TBI +CQ <0.0001 TG-TBI+CQ vs. WT-TBI+CQ = 0.0012
9E. GAP43	4	Normal distribution	One-way RM ANOVA, Bonferroni post hoc	F (3, 12) = 43.84	WT-TBI vs. TG-TBI <0.0001 WT-TBI vs. WT-TBI+CQ >0.9999 TG-TBI vs. TG-TBI +CQ <0.0001 TG-TBI+CQ vs. WT-TBI+CQ >0.9999
10B	4	Normal distribution	One-way RM ANOVA, Bonferroni post hoc	F (3, 12) = 84.16	TG-sham vs. WT-sham >0.9999 WT-TBI vs. WT-sham <0.0001 TG-TBI vs. TG-sham <0.0001 TG-TBI vs. WT-TBI = 0.2218
10G	BAG3 = 10 SQSTM1 = 15 LC3 = 10 NeuN = 10	Normal distribution	One-way RM ANOVA, Bonferroni post hoc	F (3, 41) = 179.8	BAG3 vs. NeuN <0.0001 SQSTM1 vs. NeuN <0.0001 LC3 vs. NeuN <0.0001
11C	BAG3 = 13 SQSTM1 = 12 HSPB2 = 13 β-Tubulin = 14	Normal distribution	One-way RM ANOVA, Bonferroni post hoc	F (3, 48) = 120.5	BAG3 vs. β-Tubulin <0.0001 SQSTM1 vs. β-Tubulin <0.0001 HSPB2 vs. β-Tubulin <0.0001
11E. βAPP&SQSTM1	shSCR=18 shBAG3 = 19	Normal distribution	Unpaired t test, two-tailed		0.0184
11E. βAPP&LC3	shSCR=22 shBAG3 = 17	Normal distribution	Unpaired t test, two-tailed		0.0001
11F. SQSTM1	4	Normal distribution	One-way RM ANOVA, Bonferroni post hoc	F (3, 12) = 3.987	shSCR+4-OHT vs. shSCR = 0.0871 shBAG3 vs. shSCR = 0.5720 shBAG3+4-OHT vs. shSCR+4-OHT = 0.0873 shBAG3+4-OHT vs. shBAG3 = 0.5730
11F. LC3- II / I	4	Normal distribution	One-way RM ANOVA, Bonferroni post hoc	F (3, 12) = 27.94	shSCR+4-OHT vs. shSCR = 0.0003 shBAG3 vs. shSCR >0.9999 shBAG3+4-OHT vs. shSCR+4-OHT <0.0001 shBAG3+4-OHT vs. shBAG3 = 0.4613

11F. LC3-Ⅱ/β-actin	4	Normal distribution	One-way RM ANOVA, Bonferroni post hoc	F (3, 12) = 17.87	shSCR+4-OHT vs. shSCR = 0.0091<br>shBAG3 vs. shSCR = 0.9772<br>shBAG3+4-OHT vs. shSCR+4-OHT <0.0001 shBAG3+4-OHT vs. shBAG3 >0.9999
12C.CC	WT-sham = 4<br>TG-sham = 4<br>WT-TBI = 6<br>TG-TBI = 5<br>WT-TBI+CQ = 5<br>TG-TBI+CQ = 5	Normal distribution	One-way RM ANOVA, Bonferroni post hoc	F (3, 14) = 113.1	TG-sham vs. WT-sham >0.9999<br>WT-TBI vs. WT-sham <0.0001<br>TG-TBI vs. TG-sham <0.0001<br>TG-TBI vs. WT-TBI = 0.0140<br>WT-TBI+CQ vs. WT-TBI = 0.0011<br>TG-TBI+CQ vs. TG-TBI <0.0001<br>TG-TBI+CQ vs. WT-TBI+CQ >0.9999
12C.EC	WT-sham = 4<br>TG-sham = 4<br>WT-TBI = 5<br>TG-TBI = 5<br>WT-TBI+CQ = 5<br>TG-TBI+CQ = 5	Normal distribution	One-way RM ANOVA, Bonferroni post hoc	F (3, 14) = 16.51	TG-sham vs. WT-sham >0.9999<br>WT-TBI vs. WT-sham = 0.0003<br>TG-TBI vs. TG-sham = 0.3304<br>TG-TBI vs. WT-TBI = 0.0044<br>WT-TBI+CQ vs. WT-TBI = 0.0540<br>TG-TBI+CQ vs. TG-TBI = 0.0054<br>TG-TBI+CQ vs. WT-TBI+CQ = 0.3843
12C.STR	WT-sham = 4<br>TG-sham = 4<br>WT-TBI = 5<br>TG-TBI = 5<br>WT-TBI+CQ = 5<br>TG-TBI+CQ = 5	Normal distribution	One-way RM ANOVA, Bonferroni post hoc	F (3, 14) = 22.00	TG-sham vs. WT-sham >0.9999<br>WT-TBI vs. WT-sham <0.0001<br>TG-TBI vs. TG-sham = 0.0138<br>TG-TBI vs. WT-TBI = 0.0190<br>WT-TBI+CQ vs. WT-TBI >0.9999<br>TG-TBI+CQ vs. TG-TBI = 0.0191<br>TG-TBI+CQ vs. WT-TBI+CQ >0.9999
13B	5	Normal distribution	Unpaired t test, two-tailed		WT-TBI vs. TG-TBI = 0.0446<br>WT-TBI vs. WT-TBI+CQ = 0.0342<br>TG-TBI vs. TG-TBI +CQ = 0.0058<br>TG-TBI+CQ vs. WT-TBI+CQ = 0.3157
13C. Body curl	WT-TBI =15<br>TG-TBI = 17<br>WT-TBI+CQ =	Normal distribution	Two-way RM	F (3, 48) = 16.83	WT-TBI vs. TG-TBI <0.0001<br>WT-TBI vs. WT-TBI+CQ = 0.5973<br>TG-TBI vs. TG-TBI +CQ = 0.0016

	10<br>TG-TBI+CQ =<br>10		ANOVA,<br>Bonferroni post<br>hoc		TG-TBI+CQ vs. WT-TBI+CQ =<br>0.1795<br>TG-TBI vs. WT-TBI:<br>d7 = 0.0380<br>d14 = 0.0390<br>d21 < 0.0001<br>d28 <0.0001<br>d35 <0.0001<br>TG-TBI vs. TG-TBI+CQ:<br>d5 = 0.0117<br>d7 = 0.0007<br>d14 = 0.0011<br>d21 = 0.0487<br>d28 = 0.5291<br>d35 = 0.5084
13C.Grid-walking	WT-TBI =15<br>TG-TBI = 17<br>WT-TBI+CQ =<br>10<br>TG-TBI+CQ =<br>10	Normal<br>distribution	Two-way RM<br>ANOVA,<br>Bonferroni post<br>hoc	F (3, 47) =<br>24.14	WT-TBI vs. TG-TBI <0.0001<br>WT-TBI vs. WT-TBI+CQ = 0.2153<br>TG-TBI vs. TG-TBI +CQ <0.0001<br>TG-TBI+CQ vs. WT-TBI+CQ >0.9999<br>TG-TBI vs. WT-TBI:<br>d3 = 0.0002<br>d5 = 0.0118<br>d7 = 0.0021<br>d14 = 0.0013<br>d21 = 0.0681<br>d28 = 0.1655<br>d35 = 0.0066<br>TG-TBI vs. TG-TBI+CQ:<br>d3 <0.0001<br>d5 = 0.0002<br>d7 <0.0001<br>d14 <0.0001<br>d21 = 0.2704<br>d28 >0.9999<br>d35 = 0.0050
13C. Rotarod	WT-TBI =15<br>TG-TBI = 17<br>WT-TBI+CQ =<br>10<br>TG-TBI+CQ =<br>10	Normal<br>distribution	Two-way RM<br>ANOVA,<br>Bonferroni post<br>hoc	F (3, 48) =<br>4.621	WT-TBI vs. TG-TBI= 0.0108<br>WT-TBI vs. WT-TBI+CQ >0.9999<br>TG-TBI vs. TG-TBI +CQ =<br>0.0904<br>TG-TBI+CQ vs. WT-TBI+CQ >0.9999<br>WT-TBI vs. TG-TBI:<br>d5: 0.0179

					d14: 0.0298<br>d21: 0.0284<br>d35: 0.0138<br>TG-TBI vs. TG-TBI +CQ:<br>d3: <0.0001<br>d7: 0.0018
14B	5	Normal distribution	One-way RM ANOVA, Bonferroni post hoc	F (2, 12) = 5.577	WT-TBI vs. TG-TBI = 0.0371<br>WT-TBI vs. TG-TBI+Delayed >0.9999<br>TG-TBI vs. TG-TBI+Delayed =0.0445
14C. Weight	WT-TBI =15<br>TG-TBI = 17<br>TG-TG-TBI+Delayed = 10	Normal distribution	Two-way RM ANOVA, Bonferroni post hoc	F (2, 39) = 0.4235	WT-TBI vs. TG-TBI >0.9999<br>WT-TBI vs. TG-TBI+Delayed >0.9999<br>TG-TBI vs. TG-TBI+Delayed >0.9999
14C. Curl	WT-TBI =15<br>TG-TBI = 17<br>TG-TG-TBI+Delayed = 10	Normal distribution	Two-way RM ANOVA, Bonferroni post hoc	F (2, 39) = 19.07	WT-TBI vs. TG-TBI <0.0001<br>WT-TBI vs. TG-TBI+Delayed >0.9999<br>TG-TBI vs. TG-TBI+Delayed <0.0001<br>WT-TBI vs. TG-TBI:<br>d7: 0.0190<br>d14: 0.0195<br>d21:<0.0001<br>d28:<0.0001<br>d35:<0.0001<br>TG-TBI vs. TG-TBI+Delayed<br>d3:0.0155<br>d5:0.0107<br>d7:0.0049<br>d14:0.0025<br>d21:0.0004<br>d28:<0.0001<br>d35:0.0019
14C. Grid-walking	WT-TBI =15<br>TG-TBI = 17<br>TG-TG-TBI+Delayed = 10	Normal distribution	Two-way RM ANOVA, Bonferroni post hoc	F (2, 38) = 14.10	WT-TBI vs. TG-TBI <0.0001<br>WT-TBI vs. TG-TBI+Delayed >0.9999<br>TG-TBI vs. TG-TBI+Delayed = 0.0014<br>WT-TBI vs. TG-TBI:<br>d3:<0.0001<br>d5:0.0059

					d7:0.0011<br>d14:0.0006<br>d21:0.0340<br>d35:0.0033<br>TG-TBI vs. TG-TBI+Delayed<br>d3:0.0010<br>d5:0.0449<br>d7:0.0025<br>d14:0.0021<br>d21:0.0370<br>d35:0.0189
14D	5	Normal distribution	One-way RM ANOVA, Bonferroni post hoc	F (3, 16) = 8.597	WT-TBI vs. TG-TBI = 0.0449<br>WT-TBI vs. WT-TBI+CQ = 0.4071<br>TG-TBI vs. TG-TBI +CQ = 0.0447<br>TG-TBI+CQ vs. WT-TBI+CQ = 0.4071
14E	5	Normal distribution	One-way RM ANOVA, Bonferroni post hoc	F (2, 12) = 5.138	WT-TBI vs. TG-TBI = 0.0689<br>WT-TBI vs. TG-TBI+Delayed >0.9999<br>TG-TBI vs. TG-TBI+Delayed =0.0386
S1D	6	Normal distribution	Unpaired t test, two-tailed		1d = 0.0018<br>3d = 0.0199<br>7d = 0.0072
S1F	6	Normal distribution	Unpaired t test, two-tailed		1d = 0.2228<br>3d = 0.3385<br>7d = 0.2533
S1G	4	Normal distribution	Unpaired t test, two-tailed		Hspb1 = 0.1030<br>Hspb2 = 0.0108<br>Hspb3 = 0.1327<br>Hspb5 = 0.1628
S1J	4	Normal distribution	Unpaired t test, two-tailed		0.0246
S2C	4	Normal distribution	Unpaired t test, two-tailed		0.0002
S2D	4	Normal distribution	Unpaired t test, two-tailed		Hspb1 = 0.3580<br>Hspb2 = 0.0087<br>Hspb3 = 0.3861<br>Hspb5 = 0.1125
S2F.HSPB2	6	Normal distribution	Unpaired t test, two-tailed		CTX: 0.0467<br>HIP: 0.0170
S2F.HA-Tag	6	Normal distribution	Unpaired t test, two-tailed		CTX: 0.0023<br>HIP: 0.0054

S2H.HSPB2	4	Normal distribution	One-way RM ANOVA, Bonferroni post hoc	F (3, 12) = 77.03	TG-sham vs. WT-sham <0.0001<br>WT-TBI vs. WT-sham >0.9999<br>TG-TBI vs. TG-sham >0.9999<br>TG-TBI vs. WT-TBI <0.0001
S2H.HA-Tag	4	Normal distribution	One-way RM ANOVA, Bonferroni post hoc	F (3, 12) = 119.8	TG-sham vs. WT-sham <0.0001<br>WT-TBI vs. WT-sham >0.9999<br>TG-TBI vs. TG-sham >0.9999<br>TG-TBI vs. WT-TBI <0.0001
S3A	WT-sham = 7<br>TG-sham = 7<br>WT-TBI = 15<br>TG-TBI = 17	Normal distribution	Two-way RM ANOVA, Bonferroni post hoc	F (3, 42) = 11.48	TG-sham vs. WT-sham = 0.8533<br>WT-TBI vs. WT-sham = 0.0286<br>TG-TBI vs. TG-sham <0.0001<br>TG-TBI vs. WT-TBI >0.9999
S3B	WT-sham = 7<br>TG-sham = 7<br>WT-TBI = 15<br>TG-TBI = 17	Normal distribution	Two-way RM ANOVA, Bonferroni post hoc	F (3, 20) = 21.41	TG-sham vs. WT-sham >0.9999<br>WT-TBI vs. WT-sham = 0.0011<br>TG-TBI vs. TG-sham <0.0001<br>TG-TBI vs. WT-TBI >0.9999
S3D. Latency	WT-sham = 7<br>TG-sham = 7<br>WT-TBI = 10<br>TG-TBI = 15	Normal distribution	Two-way RM ANOVA, Bonferroni post hoc	F (3, 36) = 12.24	TG-sham vs. WT-sham = 0.2754<br>WT-TBI vs. WT-sham = 0.0058<br>TG-TBI vs. TG-sham = 0.0089<br>TG-TBI vs. WT-TBI = 0.4110
S3D. Swimming velocity	WT-sham = 7<br>TG-sham = 7<br>WT-TBI = 14<br>TG-TBI = 15	Normal distribution	One-way RM ANOVA, Bonferroni post hoc	F (3, 34) = 2.425	TG-sham vs. WT-sham >0.9999<br>WT-TBI vs. WT-sham = 0.2410<br>TG-TBI vs. TG-sham = 0.8684<br>TG-TBI vs. WT-TBI >0.9999
S3D. Times	WT-sham = 7<br>TG-sham = 7<br>WT-TBI = 14<br>TG-TBI = 15	Normal distribution	One-way RM ANOVA, Bonferroni post hoc	F (3, 35) = 5.379	TG-sham vs. WT-sham >0.9999<br>WT-TBI vs. WT-sham = 0.0198<br>TG-TBI vs. TG-sham = 0.1308<br>TG-TBI vs. WT-TBI >0.9999
S3D. Quadrant	WT-sham = 7<br>TG-sham = 7<br>WT-TBI = 14<br>TG-TBI = 15	Normal distribution	One-way RM ANOVA, Bonferroni post hoc	F (3, 33) = 10.28	TG-sham vs. WT-sham =0.0531<br>WT-TBI vs. WT-sham = 0.0003<br>TG-TBI vs. TG-sham = 0.4549<br>TG-TBI vs. WT-TBI >0.9999
S3E.Weight	WT-sham = 6<br>TG-sham = 6<br>WT-TBI = 9<br>TG-TBI = 8	Normal distribution	Two-way RM ANOVA, Bonferroni post hoc	F (3, 23) = 2.580	TG-sham vs. WT-sham >0.9999<br>WT-TBI vs. WT-sham = 0.4693<br>TG-TBI vs. TG-sham >0.9999<br>TG-TBI vs. WT-TBI = 0.2248
S3E.Body Curl	WT-sham = 6<br>TG-sham = 6<br>WT-TBI = 9<br>TG-TBI = 8	Normal distribution	Two-way RM ANOVA, Bonferroni post hoc	F (3, 23) = 58.31	TG-sham vs. WT-sham >0.9999<br>WT-TBI vs. WT-sham <0.0001<br>TG-TBI vs. TG-sham <0.0001<br>TG-TBI vs. WT-TBI <0.0001

S3E.Gri-walking	WT-sham = 6<br>TG-sham = 6<br>WT-TBI = 9<br>TG-TBI = 8	Normal distribution	Two-way RM ANOVA, Bonferroni post hoc	F (3, 21) = 56.75	TG-sham vs. WT-sham >0.9999<br>WT-TBI vs. WT-sham <0.0001<br>TG-TBI vs. TG-sham <0.0001<br>TG-TBI vs. WT-TBI =0.0289
S3E.Rotarod	WT-sham = 6<br>TG-sham = 6<br>WT-TBI = 9<br>TG-TBI = 8	Normal distribution	Two-way RM ANOVA, Bonferroni post hoc	F (3, 21) = 28.88	TG-sham vs. WT-sham >0.9999<br>WT-TBI vs. WT-sham <0.0001<br>TG-TBI vs. TG-sham = 0.0004<br>TG-TBI vs. WT-TBI =0.0379
S4C. 3d	WT-TBI = 6<br>TG-TBI = 5	Normal distribution	Two-way RM ANOVA, Bonferroni post hoc	F (1, 9) = 4.538	0.0620
S4C. 35d	5	Normal distribution	Two-way RM ANOVA, Bonferroni post hoc	F (1, 8) = 7.819	0.0233
S4C. 56d	WT-TBI = 6<br>TG-TBI = 7	Normal distribution	Two-way RM ANOVA, Bonferroni post hoc	F (1, 10) = 4.988	0.0496
S5E. EC	5	Normal distribution	Two-way RM ANOVA, Bonferroni post hoc	F (1, 8) = 6.355	0.0358<br>WT-TBI vs. TG-TBI<br>7d: >0.9999<br>28d: 0.3293<br>49: 0.0322<br>WT-TBI:<br>7d vs. 28d: 0.0203
S5E. STR	5	Normal distribution	Two-way RM ANOVA, Bonferroni post hoc	F (1, 8) = 0.7732	0.4049<br>WT-TBI vs. TG-TBI<br>7d: >0.9999<br>28d: >0.9999<br>49: 0.2388<br>TG-TBI:<br>7d vs. 28d: 0.0381<br>7d vs. 49d: 0.0248
S5F. Time-of-peak N1	WT-sham = 6<br>TG-sham = 6<br>WT-TBI = 5<br>TG-TBI = 7	Normal distribution	One-way RM ANOVA, Bonferroni post hoc	F (3, 20) = 1.187	TG-sham vs. WT-sham >0.9999<br>WT-TBI vs. WT-sham >0.9999<br>TG-TBI vs. TG-sham >0.9999<br>TG-TBI vs. WT-TBI >0.9999
S5F. Time-of-peak N2	WT-sham = 6<br>TG-sham = 6<br>WT-TBI = 5	Normal distribution	One-way RM	F (3, 20) = 3.082	TG-sham vs. WT-sham >0.9999<br>WT-TBI vs. WT-sham = 0.3508<br>TG-TBI vs. TG-sham >0.9999

	TG-TBI = 7		ANOVA, Bonferroni post hoc		TG-TBI vs. WT-TBI = 0.0409
S5F. Half-width N1	WT-sham = 6<br>TG-sham = 6<br>WT-TBI = 5<br>TG-TBI = 7	Normal distribution	One-way RM ANOVA, Bonferroni post hoc	F (3, 20) = 2.662	TG-sham vs. WT-sham >0.9999<br>WT-TBI vs. WT-sham >0.9999<br>TG-TBI vs. TG-sham = 0.1143<br>TG-TBI vs. WT-TBI = 0.6336
S5F. Half-width N2	WT-sham = 6<br>TG-sham = 6<br>WT-TBI = 5<br>TG-TBI = 7	Normal distribution	One-way RM ANOVA, Bonferroni post hoc	F (3, 20) = 3.252	TG-sham vs. WT-sham >0.9999<br>WT-TBI vs. WT-sham = 0.1608<br>TG-TBI vs. TG-sham >0.9999<br>TG-TBI vs. WT-TBI = 0.0479
S5G. Time of peak	12	Normal distribution	Pearson correlation test	r = 0.6521	0.0216
S5G. Half width	12	Normal distribution	Pearson correlation test	r = 0.6527	0.0214
S5I	5	Normal distribution	One-way RM ANOVA, Bonferroni post hoc	F (3, 16) = 35.45	TG-sham vs. WT-sham >0.9999<br>WT-TBI vs. WT-sham <0.0001<br>TG-TBI vs. TG-sham = 0.0007<br>TG-TBI vs. WT-TBI = 0.0107
S7C. CC	5	Normal distribution	One-way RM ANOVA, Bonferroni post hoc	F (3, 16) = 4.201	TG-sham vs. WT-sham = 0.6281<br>WT-TBI vs. WT-sham = 0.0165<br>TG-TBI vs. TG-sham >0.9999<br>TG-TBI vs. WT-TBI = 0.8747
S7C. FN	5	Normal distribution	One-way RM ANOVA, Bonferroni post hoc	F (3, 16) = 4.571	TG-sham vs. WT-sham >0.9999<br>WT-TBI vs. WT-sham = 0.0484<br>TG-TBI vs. TG-sham >0.9999<br>TG-TBI vs. WT-TBI = 0.0484
S7F	WT-sham = 9<br>TG-sham = 9<br>2m:<br>WT-TBI = 6<br>TG-TBI = 6	Normal distribution	Two-way Mix-effects ANOVA, Bonferroni post hoc	F (1, 28) = 4.763	WT-TBI vs. TG-TBI:<br>sham >0.9999<br>2m = 0.0205<br>3m = 0.1612<br>WT-TBI:

	3m:<br>WT-TBI = 15<br>TG-TBI = 15				Sham vs. 2m = 0.0248<br>Sham vs. 3m= 0.0348<br>TG-TBI:<br>Sham vs. 2m = 0.0168<br>Sham vs. 3m= 0.0016
S7G	WT-sham = 9<br>TG-sham = 9<br>2m:<br>WT-TBI = 6<br>TG-TBI = 6<br>3m:<br>WT-TBI = 15<br>TG-TBI = 15	Normal distribution	Two-way Mix-effects ANOVA, Bonferroni post hoc	F (1, 53) = 0.6704	WT-TBI vs. TG-TBI:<br>sham >0.9999<br>2m = 0.9321<br>3m = 0.9021<br>WT-TBI:<br>Sham vs. 2m >0.9999<br>Sham vs. 3m >0.9999<br>TG-TBI:<br>Sham vs. 2m = 0.0514<br>Sham vs. 3m >0.9999
S8A	4	Normal distribution	One-way RM ANOVA, Bonferroni post hoc	F (3, 12) = 14.82	TG-sham vs. WT-sham = 0.1843<br>WT-TBI vs. WT-sham = 0.0325<br>TG-TBI vs. TG-sham = 0.0084<br>TG-TBI vs. WT-TBI = 0.0464
S8B	4	Normal distribution	One-way RM ANOVA, Bonferroni post hoc	F (3, 12) = 14.54	WT-TBI vs. WT-TBI + CQ = 0.0009<br>WT-TBI vs. TG-TBI = 0.3324<br>TG-TBI vs. TG-TBI+ CQ = 0.0302<br>WT-TBI + CQ vs. TG-TBI+ CQ >0.9999
S8D. LC3	5	Normal distribution	One-way RM ANOVA, Bonferroni post hoc	F (3, 16) = 25.37	TG-sham vs. WT-sham = 0.0711<br>WT-TBI vs. WT-sham = 0.0058<br>TG-TBI vs. TG-sham <0.0001<br>TG-TBI vs. WT-TBI = 0.0042
S8D. SQSTM1	5	Normal distribution	One-way RM ANOVA, Bonferroni post hoc	F (3, 16) = 43.10	TG-sham vs. WT-sham = 0.0173<br>WT-TBI vs. WT-sham <0.0001<br>TG-TBI vs. TG-sham <0.0001

					TG-TBI vs. WT-TBI = 0.0498
S9C	BAG3 = 12<br>SQSTM1 = 12<br>LC3 = 10<br>NeuN = 10	Normal distribution	One-way RM ANOVA, Bonferroni post hoc	$F(3, 40) = 17.84$	BAG3 vs. NeuN <0.0001<br>SQSTM1 vs. NeuN = 0.0001<br>LC3 vs. NeuN = 0.0095
S9F	BAG3 = 13<br>SQSTM1 = 14<br>LC3 = 14<br>β-Tubulin = 13	Normal distribution	One-way RM ANOVA, Bonferroni post hoc	$F(3, 40) = 17.84$	BAG3 vs. β-Tubulin <0.0001<br>SQSTM1 vs. β-Tubulin = 0.0002<br>LC3 vs. β-Tubulin <0.0001
S10B	Control = 19<br>OGD = 20	Normal distribution	Unpaired t test, two-tailed		<0.0001
S10F	16	Normal distribution	Unpaired t test, two-tailed		0.0004
S10H. BAG3	4	Normal distribution	One-way RM ANOVA, Bonferroni post hoc	$F(3, 12) = 41.45$	shSCR vs. shSCR+4-OHT >0.9999<br>shSCR vs. shBAG3 <0.0001<br>shSCR+4-OHT vs. shBAG3+4-OHT <0.0001<br>shBAG3 vs. shBAG3+4-OHT >0.9999
S10H. HSPB2	4	Normal distribution	One-way RM ANOVA, Bonferroni post hoc	$F(3, 12) = 185.5$	shSCR vs. shSCR+4-OHT <0.0001<br>shSCR vs. shBAG3 >0.9999<br>shSCR+4-OHT vs. shBAG3+4-OHT = 0.2253<br>shBAG3 vs. shBAG3+4-OHT <0.0001
S10G. βAPP	16	Normal distribution	Unpaired t test, two-tailed		0.0187
S10G. correlation	shSCR = 17<br>shBAG3 = 15	Normal distribution	Unpaired t test, two-tailed		0.0942
S11C.CTX	WT-sham = 4<br>TG-sham = 4<br>WT-TBI = 5<br>TG-TBI = 5<br>WT-TBI+CQ = 5<br>TG-TBI+CQ = 5	Normal distribution	One-way RM ANOVA, Bonferroni post hoc	$F(5, 22) = 16.98$	TG-sham vs. WT-sham >0.9999<br>WT-TBI vs. WT-sham <0.0001<br>TG-TBI vs. TG-sham <0.0001<br>TG-TBI vs. WT-TBI = 0.0148<br>WT-TBI+CQ vs. WT-TBI >0.9999<br>TG-TBI+CQ vs. TG-TBI =0.0029<br>TG-TBI+CQ vs. WT-TBI+CQ >0.9999

S11C.CC	WT-sham = 4<br>TG-sham = 4<br>WT-TBI = 5<br>TG-TBI = 5<br>WT-TBI+CQ = 5<br>TG-TBI+CQ = 5	Normal distribution	One-way RM ANOVA, Bonferroni post hoc	F (5, 22) = 16.98	TG-sham vs. WT-sham >0.9999<br>WT-TBI vs. WT-sham = 0.0005<br>TG-TBI vs. TG-sham = 0.7935<br>TG-TBI vs. WT-TBI = 0.0107<br>WT-TBI+CQ vs. WT-TBI >0.9999<br>TG-TBI+CQ vs. TG-TBI =0.0029<br>TG-TBI+CQ vs. WT-TBI+CQ >0.9999
S11C.EC	WT-sham = 4<br>TG-sham = 4<br>WT-TBI = 5<br>TG-TBI = 5<br>WT-TBI+CQ = 5<br>TG-TBI+CQ = 5	Normal distribution	One-way RM ANOVA, Bonferroni post hoc	F (5, 21) = 27.18	TG-sham vs. WT-sham >0.9999<br>WT-TBI vs. WT-sham = 0.0001<br>TG-TBI vs. TG-sham = 0.0994<br>TG-TBI vs. WT-TBI = 0.0127<br>WT-TBI+CQ vs. WT-TBI >0.9999<br>TG-TBI+CQ vs. TG-TBI =0.0111<br>TG-TBI+CQ vs. WT-TBI+CQ >0.9999
S11C. HIP	WT-sham = 4<br>TG-sham = 4<br>WT-TBI = 5<br>TG-TBI = 5<br>WT-TBI+CQ = 5<br>TG-TBI+CQ = 5	Normal distribution	One-way RM ANOVA, Bonferroni post hoc	F (5, 20) = 4.397	TG-sham vs. WT-sham >0.9999<br>WT-TBI vs. WT-sham = 0.0346<br>TG-TBI vs. TG-sham >0.9999<br>TG-TBI vs. WT-TBI = 0.3261<br>WT-TBI+CQ vs. WT-TBI >0.9999<br>TG-TBI+CQ vs. TG-TBI >0.9999<br>TG-TBI+CQ vs. WT-TBI+CQ >0.9999
S11C. STR	WT-sham = 4<br>TG-sham = 4<br>WT-TBI = 5<br>TG-TBI = 5<br>WT-TBI+CQ = 5<br>TG-TBI+CQ = 5	Normal distribution	One-way RM ANOVA, Bonferroni post hoc	F (5, 22) = 8.596	TG-sham vs. WT-sham >0.9999<br>WT-TBI vs. WT-sham = 0.1534<br>TG-TBI vs. TG-sham >0.9999<br>TG-TBI vs. WT-TBI = 0.6715<br>WT-TBI+CQ vs. WT-TBI =0.2012<br>TG-TBI+CQ vs. TG-TBI =0.0122<br>TG-TBI+CQ vs. WT-TBI+CQ >0.9999

S12A	WT-TBI =15<br>TG-TBI = 17<br>WT-TBI+CQ = 10<br>TG-TBI+CQ = 10	Normal distribution	Two-way RM ANOVA, Bonferroni post hoc	F (3, 48) = 1.200	WT-TBI vs. TG-TBI >0.9999<br>WT-TBI vs. WT-TBI+CQ = 0.7507<br>TG-TBI vs. TG-TBI +CQ >0.9999<br>TG-TBI+CQ vs. WT-TBI+CQ >0.9999
S12B	5	Normal distribution	One-way RM ANOVA, Bonferroni post hoc	F (5, 24) = 3.645	WT-sham vs. TG-sham >0.9999<br>WT-sham vs. WT-TBI >0.9999<br>WT-sham vs. WT-TBI+CQ =0.1427<br>TG-sham vs. TG-TBI >0.9999<br>TG-sham vs. TG-TBI+CQ >0.9999<br>WT-TBI vs. TG-TBI >0.9999<br>WT-TBI vs. WT-TBI+CQ = 0.6408<br>TG-TBI vs. TG-TBI+CQ >0.9999<br>WT-TBI+CQ vs. TG-TBI+CQ >0.9999
S12C. CC	5	Normal distribution	One-way RM ANOVA, Bonferroni post hoc	F (3, 16) = 2.381	WT-TBI vs. TG-TBI = 0.3464<br>WT-TBI vs. WT-TBI+CQ >0.9999<br>TG-TBI vs. TG-TBI +CQ = 0.2115<br>TG-TBI+CQ vs. WT-TBI+CQ >0.9999
S12C. FN	5	Normal distribution	One-way RM ANOVA, Bonferroni post hoc	F (3, 16) = 9.919	WT-TBI vs. TG-TBI = 0.0232<br>WT-TBI vs. WT-TBI+CQ = 0.4559<br>TG-TBI vs. TG-TBI +CQ = 0.0095<br>TG-TBI+CQ vs. WT-TBI+CQ = 0.9565
S12D. CC	5	Normal distribution	One-way RM ANOVA, Bonferroni post hoc	F (2, 12) = 2.481	WT-TBI vs. TG-TBI = 0.1432<br>WT-TBI vs. TG-TBI+Delayed = 0.5801<br>TG-TBI vs. TG-TBI+Delayed >0.9999
S12D. FN	5	Normal distribution	One-way RM ANOVA, Bonferroni post hoc	F (2, 12) = 4.606	WT-TBI vs. TG-TBI = 0.0495<br>WT-TBI vs. TG-TBI+Delayed >0.9999<br>TG-TBI vs. TG-TBI+Delayed =0.0940
